# B(C_6_F_5_)_3_ Co-Catalyst Promotes Unconventional Halide Abstraction from Grubbs I to Enhance Reactivity and Limit Decomposition

**DOI:** 10.1021/acs.organomet.4c00178

**Published:** 2024-10-23

**Authors:** Austin W. Medley, Diya Patel, Calvin Utne, Trandon A. Bender

**Affiliations:** Department of Chemistry and Biochemistry, Old Dominion University, 4501 Elkhorn Avenue, Norfolk, Virginia 23529, United States

## Abstract

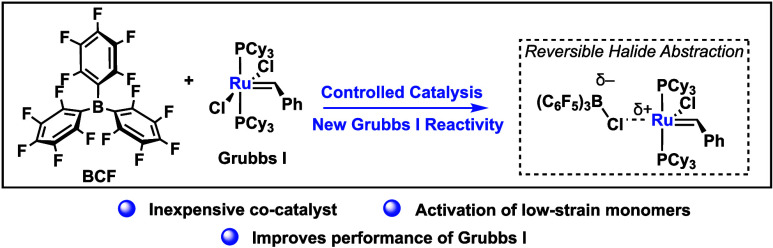

Ruthenium based Grubbs metathesis has become a commonplace reaction for synthetic chemists. Development of new generations of catalysts evolving from Grubbs I (GI) have led to greater stability, functional group compatibility, and superior reactivities. However, these advancements lead to increased costs. To this end, we report here how the addition of the commercially available tris(pentafluorophenyl)borane Lewis acid, which has become a common place catalyst in its own right, leads to enhanced reactivity of GI. Moreover, the increased reactivity arises via halide abstraction rather than traditional phosphine dissociation, providing ring-opening metathesis polymerization products that are divergent from those synthesized without the Lewis acid cocatalyst.

## Introduction

Over recent decades, ruthenium-catalyzed olefin metathesis has gained significant interest within the chemistry community. Due to its versatility in rapidly constructing carbon–carbon double bonds through reactions such as acyclic homometathesis, cross-metathesis (CM), ring-closing metathesis, and ring-opening metathesis polymerization (ROMP), there is continued effort to develop robust catalysts of this nature.^1−6^

The first-generation ruthenium metathesis catalyst (PCy_3_)_2_Cl_2_Ru=CHPh (Grubbs I, GI), was initially found to be active, though it was prone to decomposition processes.^7,8^ Efforts to prevent phosphine reinsertion and subsequent decomposition led to the development of catalysts with improved ligands and alkylidene precursors, resulting in more reactive variants. Early attempts to enhance CM catalyst reactivity involved the addition of stoichiometric or substoichiometric cofactors such as alkyl or metal chlorides, including R_4_Sn or EtAlCl_2_.^9^^,^^10^ However, these approaches were quickly surpassed by advancements in ligand design, which improved catalyst robustness.^11−14^

**Figure 1:**
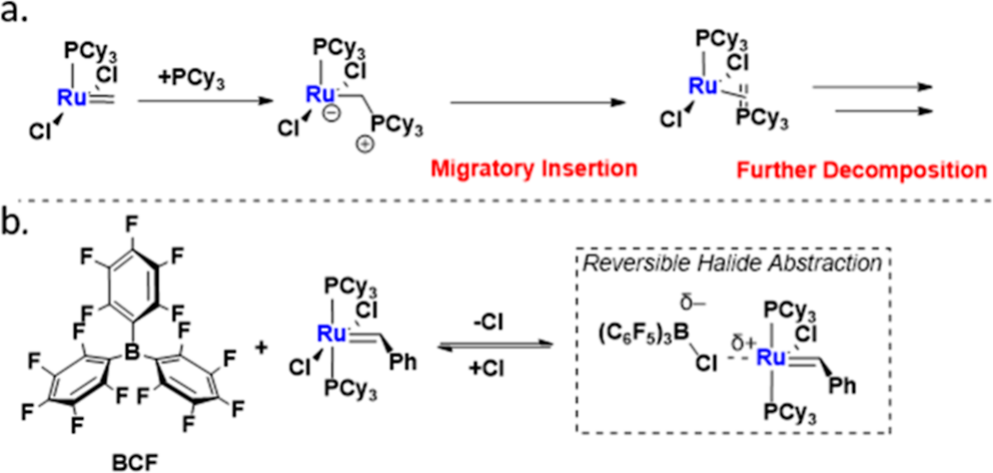
(a) Generalized phosphine mediated GI decomposition pathway. (b) Activation of GI via a reversible chloride abstraction pathway.

Consequently, the original GI catalyst evolved over time, with enhanced ligand scaffolds that minimized decomposition and improved initiation.^4,11,15−18^ These improvements reduced catalyst decomposition, which remains the primary metric for evaluating efficacy in olefin metathesis reactions.^19,20,27,28^ A conventional decomposition pathway for ruthenium-alkylidene metathesis complexes involves phosphine reinsertion into the alkylidene, leading to phosphine-ylide elimination and subsequent olefin isomerization (Figure 1a).^21−24^

The dissociation of PCy_3_ from the ruthenium center is necessary for catalyst activation, but also contributes to phosphine reinsertion and catalyst decomposition. To overcome this, new ligand sets have been developed, which have significantly increased costs when compared to the earlier GI generation. An alternative approach involves the use of additives, such as copper salts and boron Lewis acids, which coordinate to phosphines. As shown by Simocko and Wagener, these additives not only enhance metathesis reactivity but also reduce isomerization.^25^

Despite the progress made with these additives, highly electrophilic perfluorinated boron Lewis acids, such as B(C_6_F_5_)_3_ (BCF), have not yet been explored in combination with Grubbs catalysts. Given the well-documented frustrated Lewis pair behavior of borane–phosphine adducts, we hypothesized that new reaction pathways might be viable (Figure 1b).^25,26,30^

In this work, we report an unexpected finding where BCF, instead of promoting phosphine dissociation and preventing reinsertion decomposition pathways, abstracts a halide from the Ru metal center in GI. This halide abstraction provides enhanced reactivity compared to GI alone. Herein, we disclose our initial mechanistic elucidation of this pathway, as well as its improvement in the efficiency of GI in olefin metathesis and ROMP.

## Results and Discussion

### Optimization and Olefin Metathesis

Our studies began by exploring various stoichiometries of commercially available BCF and GI for the metathesis of decene to determine how the combination impacts reactivity. We found that the ratio of BCF to GI significantly influenced both the conversion of starting material and the extent to which products isomerized (Table 1).

**Table 1:**
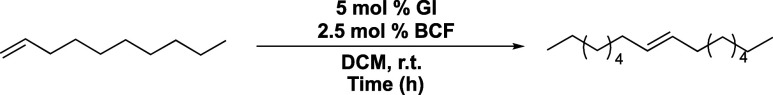
Initial Screening and Optimization of Metathesis Reactions

entry	deviation from standard conditions	time (h)	conversion (%)^a^	isomerization (%)^a^
1	none	0.5	99	6
2	0 mol % BCF	20	65	13
3	5 mol % BCF	4	77	49
4	10 mol % BCF	4	99	ND^b^
5	0 mol % GI	4	NR	NR
6	CDCI_3_ as solvent	4	ND^c^	ND^c^
7	2.5 mol % NaBArF	4	70	35
8	2.5 mol % AgOTs	4	52	77
9	5 mol % B(OH)_3_	4	55	0

a Analyzed by GC–MS with toluene as an internal standard.

b Large amount of isomerization observed by GC/MS. (Supporting Information).

c Conventional slow metathesis observed, not further quantified.

The optimal ratio of GI/BCF, 1:0.5, achieved full conversion in 30 min, with only 6% isomerization (Table 1, entry 1). In contrast, the control reaction using only GI under identical conditions required 20 h to reach 65% conversion, with 13% isomerization (Table 1, entry 2). When we increased the GI ratio to 1:1, conversion rose to 77%, while a 1:2 ratio achieved full conversion in 4 h but with significantly higher isomerization (Table 1, entries 3 and 4).^28−31^ Interestingly, the enhanced reactivity was only observed in CH_2_Cl_2_, despite screening various solvents (Table 1, entry 6). The preference for CH_2_Cl_2_ likely stems from the ability of high-dielectric solvents to facilitate ligand dissociation. Previous studies on BCF catalysis also suggest that solvent dielectric constants influence the ionization of reactive intermediates.^32^

In contrast, standard halide abstractors were less effective at promoting metathesis under the optimized conditions, producing highly isomerized products with yields comparable to that of GI alone (Table 1, entries 7 and 8). Simocko and Wagener previously reported a reduction in isomerization during olefin metathesis with GI using boranes like boronic acid, even in superstoichiometric amounts. When we applied B(OH)_3_ under our conditions (which differed from their report), the additive reduced isomerization but yielded only 55% conversion (Table 1, entry 9).^25^ Given the significant improvement in performance with BCF over B(OH)_3_, and its divergent reactivity from other halide abstractors, we sought to further understand the mechanism by which BCF activates GI.

### Mechanistic Considerations

**Figure 2:**
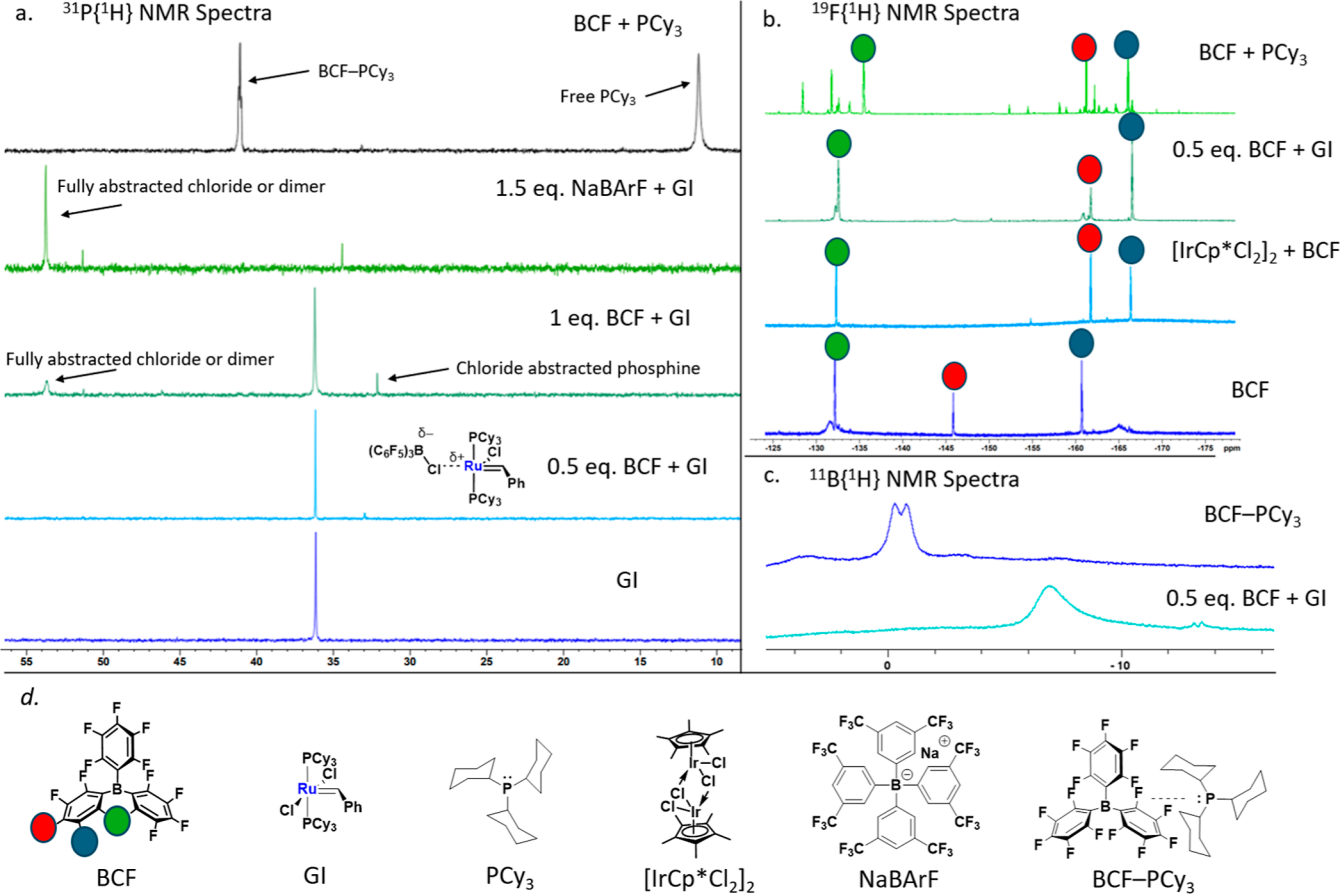
In situ NMR speciation studies conducted. a. Stacked ^31^P{^1^H} NMR spectra. b. Stacked ^19^F{^1^H} NMR spectra. c. Stacked ^11^B{^1^H} NMR spectra. d. Legend denoting all species examined in situ.

We investigated the activation of GI by BCF using in situ NMR studies. By ^19^F{^1^H} NMR, we found that a 1:0.5 ratio of GI and BCF resulted in shifts consistent with the formation of a BCF anion ([BCF][X]) rather than a BCF–PR_3_ FLP-like complex. Specifically, the para-fluorine on the aromatic ring exhibited a significant upfield shift from −146 to −162 ppm, indicating the presence of an anion. We propose that this shift arises from chloride abstraction from GI, forming a [BCF][Cl] anion (Figure 2a, see Supporting Information S30–S35).^33^ Further support for this assignment came from synthesizing an authentic [BCF][Cl] anion by combining [IrCp*Cl_2_]_2_ with BCF. The^19^F{^1^H} NMR of this [IrCp*Cl_2_]_2_/BCF mixture closely matched the GI/BCF spectrum (Figure 2b, see S34).

**Figure 3:**
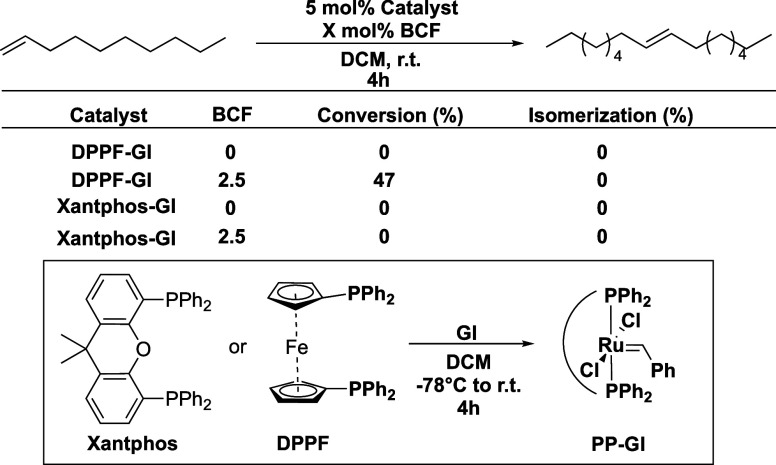
Chelated-GI activation with BCF to perform metathesis of decene through a chloride abstraction mechanism. Xantphos-GI is unreactive, while DPPF-GI with BCF remains active for CM of decene.

The^31^P{^1^H} NMR spectrum, however, was more challenging to interpret. In the GI/BCF mixture, the primary resonance observed was the unperturbed GI phosphine signal at 36 ppm, with a minor peak at 32 ppm. Authentic BCF-PCy_3_ has a chemical shift of 42 ppm in CD_2_Cl_2_, while free PCy_3_ appears at 11 ppm. Since neither of these shifts was observed with the GI/BCF mixture, we propose that the 32 ppm peak corresponds to a transiently abstracted chloride forming a [BCF][Cl] adduct with GI (Figure 3a, see Supporting Information S27–S29). Additionally, the ^11^B{^1^H} NMR spectrum of the GI/BCF mixture displayed a broad singlet at −7 ppm, a typical range for a borate anion. In contrast, the BCF–PCy_3_ showed a phosphine-coupled doublet at 0 ppm (Figure 2c, see S37).^34^

Given these findings, we sought to explore whether modifying the strength of the Lewis acid would lead to different reactivity compared to BCF. We tested two readily available analogs of BCF: H–B(C_6_F_5_)_2_ (Piers’ borane) and cyclopentyl–B(C_6_F_5_)_2_. In situ NMR studies revealed markedly different results for each borane derivative.^35−38^ When cyclopentyl–B(C_6_F_5_)_2_ was added to a solution of GI, no changes were observed in the ^19^F{^1^H} or ^31^P{^1^H} NMR spectra (see Supporting Information S28, S34), indicating that neither chloride nor phosphine abstraction was occurring. Conversely, when H–B(C_6_F_5_)_2_ was used, an anionic [H–B(C_6_F_5_)_2_][Cl] complex was observed by ^19^F{^1^H} NMR (see Supporting Information S32). However, the speciation of the GI/H–B(C_6_F_5_)_2_ mixture was more complex than that of GI/BCF. For example, a 1:1 ratio of the [H–B(C_6_F_5_)_2_][Cl] anion and the H–B(C_6_F_5_)_2_–PCy_3_ adduct was observed by ^19^F{^1^H} NMR, verified by authentic synthesis of H–B(C_6_F_5_)_2_–PCy_3_ (see Supporting Information S24 and S25). The formation of the H–B(C_6_F_5_)_2_–PCy_3_ adduct is likely due to reduced steric hindrance in H–B(C_6_F_5_)_2_ compared to BCF.

These results can be rationalized by considering the Lewis acidity of each borane.^39^ The hydride anions of BCF and H–B(C_6_F_5_)_2_ have reported Δ*G* values of 64.95 and 61.49 kcal/mol, respectively, while the cyclohexyl derivative (used as a surrogate for cyclopentyl–B(C_6_F_5_)_2_) has a Δ*G* of 53.43 kcal/mol. Thus, BCF and H–B(C_6_F_5_)_2_ are more Lewis acidic because they lack alkyl groups, which could donate back into the boron center. These boranes can abstract and stabilize a chloride anion intermediate, which is inaccessible to cyclopentyl–B(C_6_F_5_)_2_. In contrast, B(OH)_3_ has a much lower hydride Δ*G* of 7.61 kcal/mol, explaining its inability to stabilize a chloride-abstracted adduct under our conditions (Table 1, entry 9).^43^ From these results, we infer that a minimum Δ*G* must exist between H–B(C_6_F_5_)_2_ and cyclopentyl–B(C_6_F_5_)_2_ for successful halide abstraction from GI to occur.

To further support this chloride abstraction mechanism, we searched for analogous examples in the literature. Chen et al. reported the use of sodium tetrakis[3,5-bis(trifluoromethyl)phenyl]borate (NaBArF) as a halide abstractor to facilitate the copolymerization of electron-deficient comonomers with Hoveyda–Grubbs I.^40^ However, unlike our findings, their system involved irreversible halide abstraction, ultimately favoring metal dimer formation via chloride bridging. This key difference may explain why NaBArF results in poorer conversion and greater isomerization compared to BCF in decene metathesis (Table 1, entry 7).^38^

By comparing the ^31^P{^1^H} NMR spectra of NaBArF and BCF in combination with GI, we confirmed that these two additives do not form the same products. NaBArF gives a major chemical shift at 53 ppm, while BCF produces a shift at 32 ppm (Figure 2a, see S27 and S28). However, given the similar reactivity trends between NaBArF and the 1:1 BCF/GI reaction, we proposed that a similar active catalyst might be present in both cases where full abstraction occurs (Table 1, entry 3,7). This hypothesis was confirmed by combining GI/BCF (1:1) and observing the same 53 ppm shift as NaBArF/GI by ^31^P{^1^H} NMR (Figure 2a). We propose that this species represents either a fully chloride-abstracted product with solvent in the open coordination site or a dimer analogous to that reported by Chen et al. (Figure 2a).^40^

Despite these findings, examples of chloride abstraction mechanisms in Grubbs catalysts remain sparse. One notable instance is a report by Stephan et al., who used a Ru-dithiolate derivative of GI to perform metathesis via BCl_3_-mediated chloride abstraction.^41^ However, drawing parallels between this example and our work is challenging due to major differences in the catalyst structures. We sought further confirmation of the chloride abstraction mechanism by preparing a modified Grubbs catalyst with a chelating bidentate ligand. Chelating ligands are generally more resistant to dissociation from the metal center, making them ideal candidates to probe halide abstraction in the absence of phosphine dissociation.

To investigate whether chelating ligands affect the proposed chloride abstraction mechanism, we prepared a modified Grubbs catalyst with the large bite angle ligand Xantphos (Figure 3). However, Xantphos-GI proved inactive, both with and without the addition of BCF. Spek et al. had previously shown that Xantphos-GI has a noninnocent interaction between the bridging oxygen and the ruthenium center.^42^ It is proposed that this interaction prevents substrate binding, even when halide abstraction occurs making this catalyst inactive. Alternatively, the same group found that 1,1′-bis(diphenylphosphino)ferrocene (DPPF) ligated Grubbs catalysts had significantly reduced activity, even for ROMP of norbornene.

In our studies, DPPF-GI displayed no reactivity for the CM of decene, even after several days. However, when we used a 1:0.5 DPPF-GI cocatalytic mixture, 47% conversion was achieved after 4 h. Although reactivity was diminished, this reduction could be attributed to the ligand change from PCy_3_ to DPPF (Figure 3). Further speciation studies were performed on the DPPF-GI/BCF mixture. Both the ^19^F{^1^H} and ^11^B{^1^H} spectra confirmed the formation of the [BCF][Cl] adduct (see Supporting Information S35 and S38). These findings align with our optimized GI/BCF system, supporting the hypothesis that halide abstraction by BCF is the predominant pathway leading to the accelerated metathesis and limited isomerization of decene.

### Ring-Opening Metathesis Polymerization

With the discovery that borane cocatalysts enhance the reactivity of GI while reducing isomerization, we applied this approach to ROMP reactions. We hypothesized that, similar to CM, the cocatalyst would increase ROMP reactivity, potentially enabling more efficient ROMP, or reactivity with less strained cycloalkenes.^43^

We selected cyclooctene and cyclopentene as monomers for these ROMP studies. ROMP is generally efficient for cyclooctene, so we were particularly interested in the reactivity differences with cyclopentene, which is known to polymerize poorly with GI, yielding low conversions and requiring long reaction times. We predicted that the increased reactivity observed in our GI/BCF cocatalytic system would lead to more efficient ROMP, even with this recalcitrant monomer.

As shown in Table 2, the borane cocatalysts slightly increased the yield and number-average molecular weight (*M*_n_) of polymers in the ROMP of *cis*-cyclooctene, compared to reactions using only GI. Additionally, the dispersity (*D̵*) was lower for all polymers resulting from borane cocatalysis, compared to the control reactions. While conversions and yields were not significantly different, the cocatalyst clearly influenced the polymer properties.

**Table 2:**
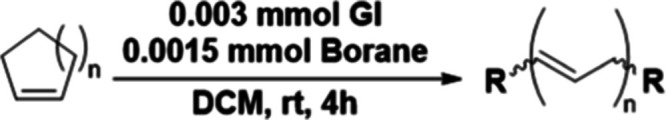

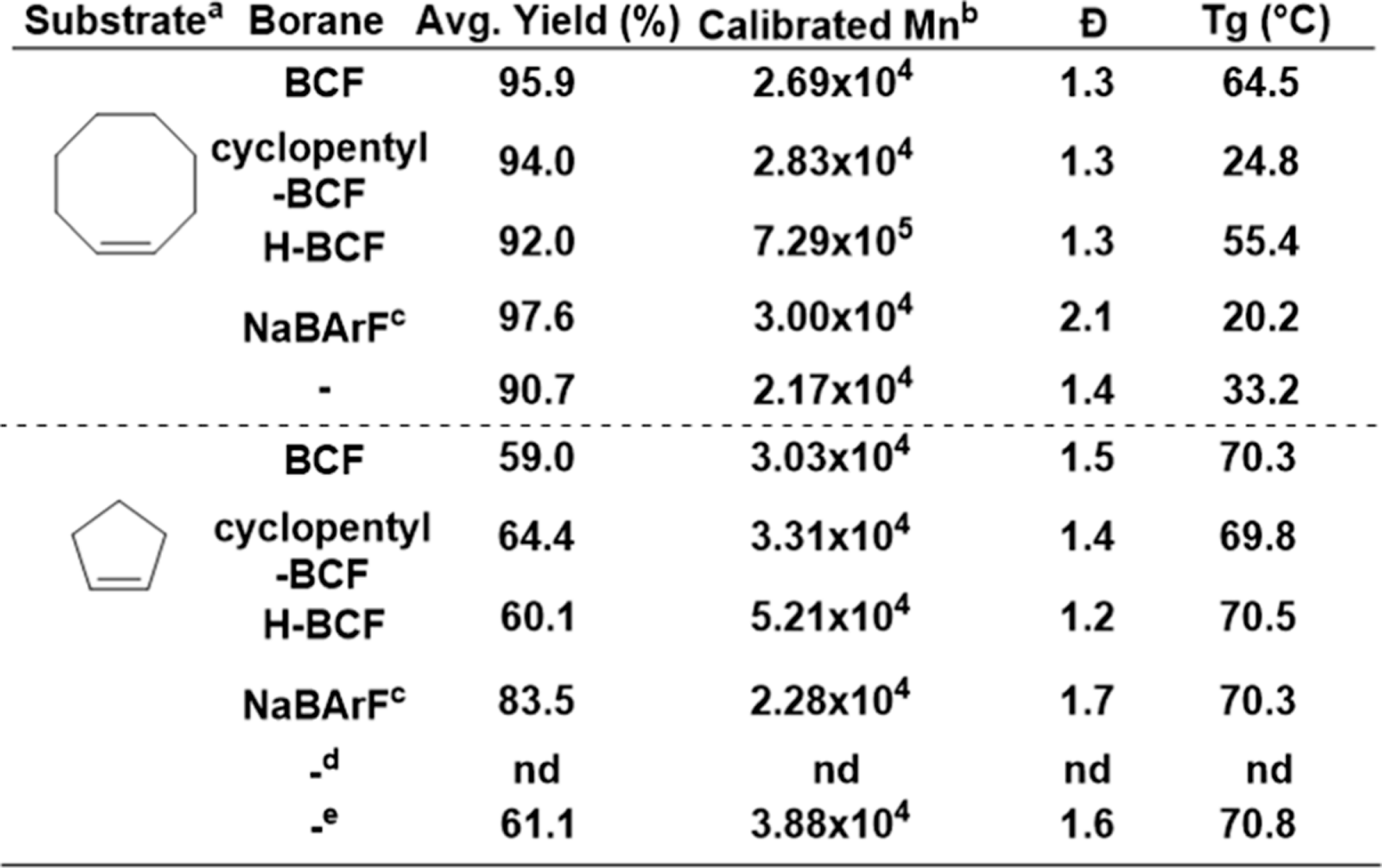
Comparative Yields/Mn/*D̵* of Various Co-catalyst Addition to GI in ROMP of COE and Cp

a 1.8 mmol of substrate (720 equiv).

b Calibration curve of polystyrene standards used to determine *M*_n_, reported as g/mol.

c 1.8 mmol substrate with 0.0025 mmol of G1 and NaBARF.

d Not determined, formed oligomeric material under std. cond.

e Reaction time was 48 h.

Cyclopentene provided a better test for the enhanced reactivity of the GI-borane system. After reacting for 4 h, all borane cocatalysts resulted in over 50% conversion (Table 2). By comparison, the GI control reaction produced no polymer under the same conditions. It was only upon extending the reaction time of GI to 48 h that 61% conversion of polycyclopentene was afforded. Furthermore, all borane cocatalyzed reactions produced polymers with a significantly higher *M*_n_. H–B(C_6_F_5_)_2_ provided the highest molecular weight polymer, while cyclopentyl–B(C_6_F_5_)_2_ and BCF also produced high molecular weights in moderate yields. In addition, the polymers produced in the cocatalyzed reactions exhibited lower dispersity, indicating more uniform polymer formation compared to the GI control. This stark contrast between the cocatalyzed ROMP reactions and those using only GI highlights the significant advantages of using boranes to activate GI in the polymerization of unstrained monomers.

As previously demonstrated, NaBArF in ROMP was found to be effective by proceeding through a halide abstraction mechanism. However, when NaBArF was used in place of BCF, the dispersity of the polymer formed was significantly higher, while the *M*_n_ was lower than most of the boron Lewis acid products. Moreover, the GPC trace showed a nonsymmetric peak and what appeared to be the presence of oligomers in addition to the polymeric material (see Supporting Information S59 and S72). This indicated that our system achieves a higher degree of control in ROMP, where controlled propagation steps yield more uniform polymers. Ultimately, the less-controlled nature of NaBArF as a cocatalyst in ROMP is consistent with the initial CM data obtained when screening with decene in Table 1.

### Relative ROMP Reactivity Studies

**Figure 4:**
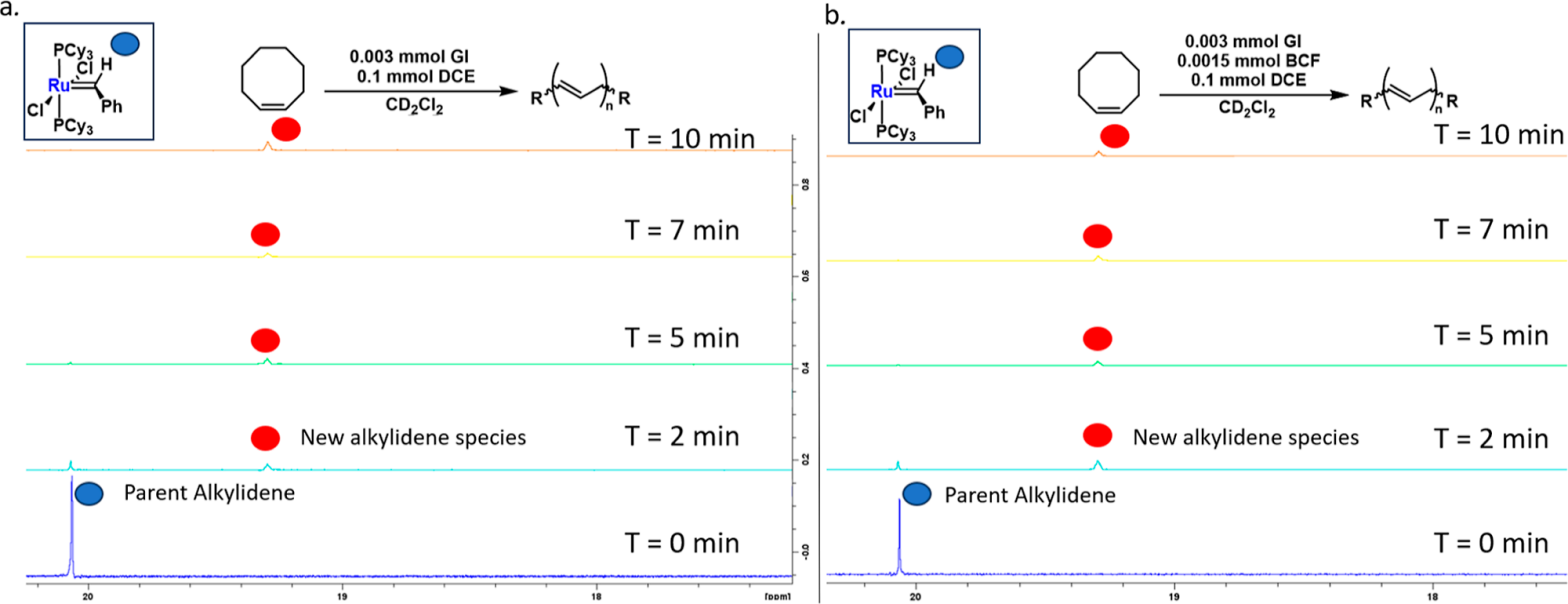
Alkylidene consumption studies and analysis of catalyst resting states (S41). (a) Stacked ^1^H NMR spectra of control reaction zoomed at the alkylidene region. (b) Stacked ^1^H NMR spectra of the reaction mixture zoomed at the alkylidene region.

To probe whether the enhanced reactivity observed in ROMP is due to differences in catalyst initiation or propagation, we conducted resting state studies using 0.1 mmol of cyclooctene (COE) as the substrate. ^1^H NMR spectra of in situ reactions were collected to compare GI and GI/BCF reaction mixtures. In both cases, nearly full conversion of the starting alkylidene to the catalyst resting state was observed (Figure 4). This rapid conversion suggests that both catalytic systems initiate at similar rates.

**Figure 5:**
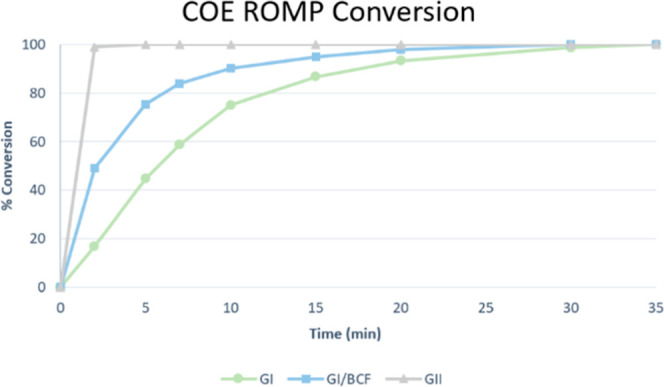
Comparative rate of conversion plot for the COE of ROMP using various catalytic systems. Experiments were conducted at 25 °C using 70 equiv of COE relative to catalyst loading in 0.5 mL of CD_2_Cl_2_ and integrated with respect to dichloroethane (DCE) as an internal standard.

Since initiation rates appeared identical, we focused on propagation. Time-course studies of COE conversion revealed that the optimized GI/BCF system propagated 55% faster than GI alone (Figure 5, see Supporting Information S42 and S43). This finding explains why polycyclopentene is formed within 4 h under cocatalytic conditions, while GI alone requires 48 h.

Although the optimized cocatalyst conditions provided a marked increase in reactivity over GI, we wanted to compare this result with Grubbs II (GII) to further define the bounds of improvement in ROMP reactivity. When the same COE ROMP conditions were applied, it was found that GII was still more reactive (Figure 5). As a point of comparison, in 2 min, GII provided 99% conversion, GI/BCF yielded 49% conversion, and GI alone achieved 16.7% conversion. While GI/BCF is not as reactive as GII, it does offer significant improvement over GI alone. Moreover, this system achieves these results without requiring ligand modifications or extensive revisions to the Ru metal center synthesis.

### Material Properties of ROMP Products

To further understand the influence that this divergent activation mode had on the polymeric product, differential scanning calorimetry was performed. As shown in Table 2, the resulting polymers glass transition point (*T*_g_) (°C) for those activated with boranes were higher than the control reactions, in the range of 20–30 °C for the cyclooctene suite. The propensity of a polymer to undergo uniform packing is directly proportional to its *T*_g,_ wherein more densely packed polymers yield higher glass transition points. In the context of our results, the polymers synthesized through the borane activation pathway are more uniformly packed, as illustrated by their higher glass transition points. Moreover, NMR studies of these polymers reveal predominantly *trans*-olefin polymeric product in an approximate 5:1 ratio, whereas the GI control reaction has a ratio of 3:1. However, the process or reasoning for this selectivity is not known.

While the cyclopentene ROMP product did not form under standard reaction conditions, we were able to compare properties from the sample made with elongated reaction times. It was found the *T*_g_ of the borane derived ROMP materials agreed with the conventional polycyclopentene product, being in the range of 69–71 °C. Ultimately, this result indicated that the borane activated ROMP conditions increased reactivity toward cyclopentene, though it yielded a similar material to an authentically made standard.

## Summary

In summary, it was discovered that Lewis-acidic boranes, in combination with GI, result in increased reactivity for CM and ROMP. Speciation studies indicated that the mechanism of activation proceeded via dynamic halide abstraction rather than a conventional phosphine dissociation pathway—a phenomenon exclusive to highly Lewis-acidic boranes. Moreover, the transient abstraction of the halide, rather than one driven by precipitation, facilitated a more controlled metathesis.

Following this discovery, the application to ROMP was explored. With this GI/borane system, polymer products were found to have higher molecular weights and more uniform dispersity compared to those produced with GI alone. This result indicated a more controlled and uniform propagation of the catalyst. In the activation of unstrained monomers, the GI/borane systems outperformed GI alone, yielding higher molecular weight polymers and greater yields. Cyclopentene was activated by the GI-borane system, improving the yield by over 50% compared to GI alone, while also increasing the Mn of the polymer formed.

Preliminary kinetic studies of the ROMP of cyclooctene revealed that the GI-borane cocatalyst system was 55% more efficient than GI alone. Of further interest is how these Lewis-acidic boranes modulate the reactivity of other Grubbs catalysts, and whether their potential for nonconventional chemical reactivity can be realized. This synthetic approach highlights the utility of highly Lewis-acidic boranes in cocatalyzed metathesis reactions and provides a pathway for other at-metal modifications of transition metal catalysts.

## Experimental Section

### General Methods

All reactions unless otherwise stated were carried out in oven (130 °C) dried glassware under an inert atmosphere using standard Schlenk techniques. Unless otherwise specified, all reactions were conducted at ambient temperature (25 °C, rt).

#### Piers-Borane (H–BCF) Synthetic Procedure

Piers-Borane was prepared according to known literature procedure. To a 20 mL scintillation vial charged with a stir bar was added tris(pentafluoro)phenylborane (500 mg, 0.98 mmol) and this was dissolved in benzene (10 mL). To this triethylsilane (0.12 g, 0.99 mmol) was added. The vial was capped, brought to 60 °C, and allowed to stir for 3 days. The solution was quickly filtered over a frit and the supernatant collected. The material began crashing out of solution and was collected on by filtration. Volatiles were removed to yield a white solid, 0.22 g, 68% in pure form. Spectra matched reported.^38^

#### Cp-BCF Synthetic Procedure

To a 20 mL scintillation vial charged with a stir bar was added 100 mg H-BCF (0.29 mmol, 1 equiv) and 0.300 L of cyclopentene (2.86 mmol, 9.9 equiv). The mixture was diluted to 5 mL with THF and stirred for 4 h at room temperature. Solvent and excess cyclopentene were removed in vacuo to afford 81 mg of Cp-BCF in 68% yield, without the need of further purification.

#### DPPF-GI Synthetic Procedure

GI-DPPF was prepared according to literature procedure.^42^ A solution of 100 mg GI (0.12 mmol, 1 equiv) in 15 mL of DCM cooled to −78 °C. To this solution was added 67 mg of DPPF (0.12 mmol, 1 equiv) in 5 mL DCM. The reaction was stirred at −78 °C for 20 min and then allowed to warm to room temperature. After another hour, the reaction volume was reduced by half under reduced atmosphere. Fifteen mL of pentane was then added to the reaction mixture to precipitate a green solid. The solid was isolated and reprecipitated twice out of DCM/pentane and dried in vacuo to yield green-brown product GI-DPPF in 40% yield (39 mg, 0.048 mmol). Spectra matched that which was previously reported in the chemical literature.

#### Xantphos-GI Synthetic Procedure

GI-xantphos was prepared according to literature procedure.^42^ A solution of 100 mg GI (0.12 mmol, 1 equiv) in 15 mL of DCM cooled to −78 °C. To this solution was added 69 mg of DPPF (0.12 mmol, 1 equiv) in 5 mL DCM. The reaction was stirred at −78 °C for 20 min and then allowed to warm to room temperature. After another hour, the reaction volume was reduced by half under reduced atmosphere. Fifteen mL of pentane was then added to the reaction mixture to precipitate a green solid. The solid was isolated and reprecipitated twice out of DCM/pentane and dried in vacuo to yield green-brown product GI-DPPF in 40% yield (40 mg, 0.048 mmol). Spectra matched that which was previously reported in the chemical literature.

### General Experimental Procedures

#### General Synthetic Procedure for Control Metathesis Reactions

Reactions were performed within an inert atmosphere glovebox in 1 dram screw capped vials. To those vials, affixed with screw-capped pressure septa, was added 4.1 mg of Grubbs 1 (0.005 mmol) and 500 μL of DCM. Dry and degassed decene (0.1 mmol, 18.9 μL) were injected via μL syringe and allowed to stir at room temperature for 20 h. Reactions were quenched by exposing them to air, concentrating under reduced atmosphere, followed by addition of CDCl_3_. 7.91 μL of toluene was then added to the reaction as an internal standard for GC/MS and NMR characterization.

#### General Synthetic Procedure for CM Reactions

Reactions were performed within an inert atmosphere glovebox in 1 dram screw capped vials. To those vials, affixed with screw-capped pressure septa, was added 4.1 mg Grubbs 1 (0.005 mmol, 0.005 equiv), 1.3 mg tris(pentafluoro)phenylborane (BCF) (0.0025 mmol, 0.0025 equiv), and 500 μL of DCM. After 15 min to allow for abstraction to occur, dry and degassed decene was added via μL syringe (0.1 mmol, 18.9 μL) and allowed to stir at room temperature for 4 h. Reactions were quenched by exposing them to air, concentration under reduced atmosphere followed by addition of CDCl_3_ for characterization. 7.91 μL of toluene was then added to the reaction as an internal standard for GC/MS and NMR characterization.

#### General Synthetic Procedure for ROMP Reactions

To a 1-dram screw capped vial, affixed with screw-capped pressure septa, was added 2.4 mg GI (0.003 mmol, 1 equiv) and 0.7 mg tris(pentafluoro)phenylborane (BCF) (0.0015 mmol, 0.5 equiv) and 500 μL of DCM. After 15 min to allow for abstraction to occur, to these solutions were added their respective monomer (1.8 mmol, 600 equiv). The reaction was allowed to react for 20 h time, at which point the solvent was removed under reduced atmosphere. These polymers were weighed directly, and GPC samples were directly prepared from these materials.

#### General Synthetic Procedure for Cl Abstraction Studies

To a 1-dram vial was added 4.4 mg GI (0.005 mmol, 0.005 equiv) and 0.7 mg AgOTs (0.0025 mmol, 0.0025 equiv). The contents were diluted with 500 μL of CH_2_Cl_2_. After 15 min, to allow for abstraction to occur to this solution was added 18.9 μL of decene (0.1 mmol, 1 equiv) via syringe. The solution changed color from purple to dark brown rapidly and formed large amounts of precipitate. After 4 h, an aliquot was removed for GC/MS analysis.

#### General Synthetic Procedure for NaBArF Isomerization Study

To a 1-dram vial was added 4.1 mg of GI (0.005 mmol, 0.05 equiv) and 2.2 mg NaBArF (0.0025 mmol, 0.025 equiv). The contents were diluted with 500 μL of CH_2_Cl_2_. After 15 min to allow for abstraction to occur to this solution was added 18.9 μL of decene, (0.1 mmol, 1 equiv) via syringe. The solution was allowed to stir for 4 h at which time an aliquot was removed and diluted to 1 mL in hexanes for GC/MS analysis.

#### General Synthetic Procedure for NaBArF ROMP Reaction of COE

To a 1-dram vial was added 2.4 mg GI (0.003 mmol, 1 equiv) and 2.7 mg NaBArF (0.003 mmol, 1 equiv). This solution was diluted to 500 μL with CH_2_Cl_2_. After 15 min to allow for abstraction to occur, to this sample was injected 0.023 mL of cyclooctene (1.8 mmol). The reaction was allowed to stir for 4 h at which point 2 mL of ethyl vinyl ether was added to quench it. The reaction was concentrated under reduced atmosphere and filtered prior to analysis. These polymers were weighed directly, and GPC samples were directly prepared from these materials.

#### General Synthetic Procedure for NaBArF ROMP Reaction of Cp

To a 1-dram vial was added 2.4 mg GI (0.003 mmol, 1 equiv) and 2.7 mg NaBArF (0.003 mmol, 1 equiv). This solution was diluted to 500 μL with CH_2_Cl_2_. After 15 min to allow for abstraction to occur, to this sample was injected 0.09 mL of cyclopentene (1.8 mmol). The reaction was allowed to stir for 4 h at which point 2 mL of ethyl vinyl ether was added to quench it. The reaction was concentrated under reduced atmosphere and filtered prior to analysis. These polymers were weighed directly, and GPC samples were directly prepared from these materials.

#### General Synthetic Procedure for ^31^P{^1^H} Speciation Studies

To a 1-dram vial was added either 2.4 mg of GI (0.003 mmol, 1 equiv) and 0.7 mg BCF (0.0015 mmol, 0.5 equiv) or 0.7 mg BCF (0.0015 mmol, 1 equiv) and 0.4 mg P(Cy)_3_ (0.0015 mmol, 1 equiv). The contents of each were diluted with 500 μL of CD_2_Cl_2_ and transferred to NMR tubes. The tubes were then capped with rubber NMR septa and wrapped with parafilm. Spectra were taken without delay and compared to an authentic sample of Grubbs 1 in CD_2_Cl_2_.

#### General Synthetic Procedure for ^19^F{^1^H} Speciation Studies

To a 1-dram vial was added either 2.4 mg of GI (0.003 mmol, 1 equiv) and 0.7 mg BCF (0.0015 mmol, 0.5 equiv) or 0.7 mg BCF (0.0015 mmol, 1 equiv) and 0.4 mg P(Cy)_3_ (0.0015 mmol, 1 equiv). The contents of each were diluted with 500 μL of CD_2_Cl_2_ and transferred to NMR tubes. The tubes were then capped with rubber NMR septum and wrapped with parafilm. Spectra were taken without further delay and compared to an authentic sample of Grubbs 1 in CD_2_Cl_2_.

#### General Control Procedure for Grubbs 1 Alkylidene Consumption Study

To a 1-dram vial was added 2.4 mg GI (0.003 mmol, 1 equiv) and 7.91 μL of DCE (0.1 mmol). This solution was diluted to 500 μL with CD_2_Cl_2_, at which time it was transferred to an NMR tube, capped with a rubber septum, and wrapped with parafilm. The sample was injected into a 600 MHz NMR to examine the alkylidene shift. Once the parent spectra were obtained, the sample was re-ejected from the probe and 23.3 μL of cyclooctene (1.8 mmol) was added via syringe through the septum. The sample was quickly reinjected into the probe and NMR time points were taken. Alkylidene consumption was observed qualitatively, as such further propagation kinetic studies were performed.

#### General Synthetic Procedure for GI Alkylidene Consumption Study

To a 1-dram vial was added 2.4 mg GI (0.003 mmol, 1 equiv) and 7.91 μL of DCE (0.1 mmol). This solution was diluted to 500 μL with CD_2_Cl_2_, at which time it was transferred to an NMR tube, capped with a rubber septum, and wrapped with parafilm. The sample was injected into a 600 MHz NMR to examine the alkylidene shift. Once the parent spectra were obtained, the sample was re-ejected from the probe and 23.3 μL of cyclooctene (1.8 mmol) was added via syringe through the septum. The sample was quickly reinjected into the probe and NMR time points were taken. Alkylidene consumption was observed qualitatively to be the same in both the control and the patent experiment, as such further propagation kinetic studies were performed.

#### General Control Procedure for GI Kinetic Study

To a 1-dram vial was added 2.4 mg Grubbs 1 (0.003 mmol, 1 equiv) and 7.91 μL of DCE (0.1 mmol). This solution was diluted to 500 μL with CD_2_Cl_2_, at which time it was transferred to an NMR tube, capped with a rubber septum, and wrapped with parafilm. The sample was injected into a 600 MHz NMR to examine the alkylidene shift. Once the parent spectra were obtained, the sample was re-ejected from the probe and 23.3 μL of cyclooctene (1.8 mmol) was added via syringe through the septum. The sample was quickly reinjected into the probe and NMR time points were taken. Substrate consumption was integrated with respect to DCE as an internal standard.

#### General Synthetic Procedure for GI/BCF Kinetic Study

To a 1-dram vial was added 2.4 mg Grubbs 1 (0.003 mmol, 1 equiv), 0.7 mg BCF (0.0015 mmol, 0.5 equiv) and 7.91 μL of DCE (0.1 mmol). This solution was diluted to 500 μL with CD_2_Cl_2_, at which time it was transferred to an NMR tube, capped with a rubber septum, and wrapped with parafilm. The sample was injected into a 600 MHz NMR to examine the alkylidene shift. Once the parent spectra were obtained, the sample was re-ejected from the probe and 23.3 μL of cyclooctene (1.8 mmol) was added via syringe through the septum. The sample was quickly reinjected into the probe and NMR time points were taken. Substrate consumption was integrated with respect to DCE as an internal standard.

#### General Synthetic Procedure for GII Kinetic Study

To a 1-dram vial was added 2.5 mg Grubbs IIs (0.003 mmol, 1 equiv) and 7.91 μL of DCE (0.1 mmol). This solution was diluted to 500 μL with CD_2_Cl_2_, at which time it was transferred to an NMR tube, capped with a rubber septum, and wrapped with parafilm. The sample was injected into a 600 mHz NMR. Once the parent spectra were obtained, the sample was re-ejected from the probe and 23.3 μL of cyclooctene (1.8 mmol) was added via syringe through the septum. The sample was quickly reinjected into the probe and NMR time points were taken. Substrate consumption was integrated with respect to DCE as an internal standard.

## Supporting Information Available

General methods and considerations, reaction procedures, GC/MS and NMR spectra (PDF)

## Abbreviations

CM: cross metathesis
ROMP: ring-opening metathesis polymerization.

## References

[ref1] Vougioukalakis G. C.; Grubbs R. H. Ruthenium-Based Heterocyclic Carbene-Coordinated Olefin Metathesis Catalysts. Chem. Rev. 2010, 110 (3), 1746–1787. 10.1021/cr9002424. 20000700

[ref2] Schrock R. R.; Hoveyda A. H. Molybdenum and Tungsten Imido Alkylidene Complexes as Efficient Olefin-Metathesis Catalysts. Angew. Chem., Int. Ed. 2003, 42 (38), 4592–4633. 10.1002/anie.200300576. 14533149

[ref3] Lozano-Vila A. M.; Monsaert S.; Bajek A.; Verpoort F. Ruthenium-Based Olefin Metathesis Catalysts Derived from Alkynes. Chem. Rev. 2010, 110 (8), 4865–4909. 10.1021/cr900346r. 20392041

[ref4] Jiang A. J.; Zhao Y.; Schrock R. R.; Hoveyda A. H. Highly Z-Selective Metathesis Homocoupling of Terminal Olefins. J. Am. Chem. Soc. 2009, 131 (46), 16630–16631. 10.1021/ja908098t. 19919135 PMC2788204

[ref5] Diver S. T.; Giessert A. J. Enyne Metathesis (Enyne Bond Reorganization). Chem. Rev. 2004, 104 (3), 1317–1382. 10.1021/cr020009e. 15008625

[ref6] Hoveyda A. H. Evolution of Catalytic Stereoselective Olefin Metathesis: From Ancillary Transformation to Purveyor of Stereochemical Identity. J. Org. Chem. 2014, 79 (11), 4763–4792. 10.1021/jo500467z. 24720633 PMC4049245

[ref7] Schwab P.; Grubbs R. H.; Ziller J. W. Synthesis and Applications of RuCl_2_(=CHR′)(PR_3_)_2_: The Influence of the Alkylidene Moiety on Metathesis Activity. J. Am. Chem. Soc. 1996, 118 (1), 100–110. 10.1021/ja952676d.

[ref8] Nguyen S. T.; Johnson L. K.; Grubbs R. H.; Ziller J. W. Ring-Opening Metathesis Polymerization (ROMP) of Norbornene by a Group VIII Carbene Complex in Protic Media. J. Am. Chem. Soc. 1992, 114 (10), 3974–3975. 10.1021/ja00036a053.

[ref9] Calderon N.; Ofstead E. A.; Judy W. A. Ring-Opening Polymerization of Unsaturated Alicyclic Compounds. J. Polym. Sci., Part A-1: Polym. Chem. 1967, 5 (9), 2209–2217. 10.1002/pol.1967.150050901.

[ref10] Ivin K. J.; Mol J. C. Olefin Metathesis and Metathesis Polymerization; Elsevier, 1997.

[ref11] Garber S. B.; Kingsbury J. S.; Gray B. L.; Hoveyda A. H. Efficient and Recyclable Monomeric and Dendritic Ru-Based Metathesis Catalysts. J. Am. Chem. Soc. 2000, 122 (34), 8168–8179. 10.1021/ja001179g.

[ref12] Kośnik W.; Lichosyt D.; Śnieżek M.; Janaszkiewicz A.; Woźniak K.; Malińska M.; Trzaskowski B.; Kajetanowicz A.; Grela K. Ruthenium Olefin Metathesis Catalysts Bearing a Macrocyclic N-Heterocyclic Carbene Ligand: Improved Stability and Activity. Angew. Chem., Int. Ed. 2022, 61 (24), e202201472 10.1002/anie.202201472. PMC9322543 35347824

[ref13] Ogba O. M.; Warner N. C.; O’Leary D. J.; Grubbs R. H. Recent Advances in Ruthenium-Based Olefin Metathesis. Chem. Soc. Rev. 2018, 47 (12), 4510–4544. 10.1039/C8CS00027A. 29714397 PMC6107346

[ref14] Kumandin P. A.; Antonova A. S.; Novikov R. A.; Vasilyev K. A.; Vinokurova M. A.; Grigoriev M. S.; Novikov A. P.; Polianskaia D. K.; Polyanskii K. B.; Zubkov F. I. Properties and Catalytic Activity of Hoveyda–Grubbs-Type Catalysts with an S → Ru Coordination Bond in a Six-Membered Chelate Ring. Organometallics 2023, 42 (3), 218–234. 10.1021/acs.organomet.2c00556.

[ref15] Thiel V.; Hendann M.; Wannowius K.-J.; Plenio H. On the Mechanism of the Initiation Reaction in Grubbs–Hoveyda Complexes. J. Am. Chem. Soc. 2012, 134 (2), 1104–1114. 10.1021/ja208967h. 22188483

[ref16] Ou X.; Occhipinti G.; Boisvert E.-J. Y.; Jensen V. R.; Fogg D. E. Mesomeric Acceleration Counters Slow Initiation of Ruthenium–CAAC Catalysts for Olefin Metathesis (CAAC = Cyclic (Alkyl)(Amino) Carbene). ACS Catal. 2023, 13 (8), 5315–5325. 10.1021/acscatal.2c03828. 37123599 PMC10127214

[ref17] Luo S.-X. L.; Engle K. M.; Dong X.; Hejl A.; Takase M. K.; Henling L. M.; Liu P.; Houk K. N.; Grubbs R. H. An Initiation Kinetics Prediction Model Enables Rational Design of Ruthenium Olefin Metathesis Catalysts Bearing Modified Chelating Benzylidenes. ACS Catal. 2018, 8 (5), 4600–4611. 10.1021/acscatal.8b00843. 32528741 PMC7289044

[ref18] Khan R. K. M.; Torker S.; Hoveyda A. H. Readily Accessible and Easily Modifiable Ru-Based Catalysts for Efficient and Z-Selective Ring-Opening Metathesis Polymerization and Ring-Opening/Cross-Metathesis. J. Am. Chem. Soc. 2013, 135 (28), 10258–10261. 10.1021/ja404208a. 23822154

[ref19] Hong S. H.; Wenzel A. G.; Salguero T. T.; Day M. W.; Grubbs R. H. Decomposition of Ruthenium Olefin Metathesis Catalysts. J. Am. Chem. Soc. 2007, 129 (25), 7961–7968. 10.1021/ja0713577. 17547403

[ref20] Ashworth I. W.; Hillier I. H.; Nelson D. J.; Percy J. M.; Vincent M. A. What Is the Initiation Step of the Grubbs-Hoveyda Olefin Metathesis Catalyst?. Chem. Commun. 2011, 47 (19), 5428–5430. 10.1039/C1CC11230A. 21483970

[ref21] Lummiss J. A. M.; McClennan W. L.; McDonald R.; Fogg D. E. Donor-Induced Decomposition of the Grubbs Catalysts: An Intercepted Intermediate. Organometallics 2014, 33 (23), 6738–6741. 10.1021/om501011y.

[ref22] Occhipinti G.; Nascimento D. L.; Foscato M.; Fogg D. E.; Jensen V. R. The Janus Face of High Trans-Effect Carbenes in Olefin Metathesis: Gateway to Both Productivity and Decomposition. Chem. Sci. 2022, 13 (18), 5107–5117. 10.1039/D2SC00855F. 35655574 PMC9093171

[ref23] McClennan W. L.; Rufh S. A.; Lummiss J. A. M.; Fogg D. E. A General Decomposition Pathway for Phosphine-Stabilized Metathesis Catalysts: Lewis Donors Accelerate Methylidene Abstraction. J. Am. Chem. Soc. 2016, 138 (44), 14668–14677. 10.1021/jacs.6b08372. 27736083

[ref24] Bailey G. A.; Fogg D. E. Acrylate Metathesis via the Second-Generation Grubbs Catalyst: Unexpected Pathways Enabled by a PCy3-Generated Enolate. J. Am. Chem. Soc. 2015, 137 (23), 7318–7321. 10.1021/jacs.5b04524. 26030596

[ref25] Simocko C.; Wagener K. B. Effects of Boron-Containing Lewis Acids on Olefin Metathesis. Organometallics 2013, 32 (9), 2513–2516. 10.1021/om400257b.

[ref26] Stephan D. W. Catalysis, FLPs, and Beyond. Chem 2020, 6 (7), 1520–1526. 10.1016/j.chempr.2020.05.007.

[ref27] Lawson J. R.; Melen R. L. Tris(Pentafluorophenyl)Borane and Beyond: Modern Advances in Borylation Chemistry. Inorg. Chem. 2017, 56 (15), 8627–8643. 10.1021/acs.inorgchem.6b02911. 28157303

[ref28] Higman C. S.; Plais L.; Fogg D. E. Isomerization During Olefin Metathesis: An Assessment of Potential Catalyst Culprits. ChemCatChem 2013, 5 (12), 3548–3551. 10.1002/cctc.201300886.

[ref29] Hong S. H.; Sanders D. P.; Lee C. W.; Grubbs R. H. Prevention of Undesirable Isomerization during Olefin Metathesis. J. Am. Chem. Soc. 2005, 127 (49), 17160–17161. 10.1021/ja052939w. 16332044

[ref30] Perdriau S.; Chang M.-C.; Otten E.; Heeres H. J.; de Vries J. G. Alkene Isomerisation Catalysed by a Ruthenium PNN Pincer Complex. Chem.—Eur. J. 2014, 20 (47), 15434–15442. 10.1002/chem.201403236. 25264262

[ref31] Sanz-Navarro S.; Mon M.; Doménech-Carbó A.; Greco R.; Sánchez-Quesada J.; Espinós-Ferri E.; Leyva-Pérez A. Parts–per–Million of Ruthenium Catalyze the Selective Chain–Walking Reaction of Terminal Alkenes. Nat. Commun. 2022, 13 (1), 2831 10.1038/s41467-022-30320-9. 35595741 PMC9123009

[ref32] Lowe J. M.; Bowers B. E.; Seo Y.; Gagné M. R. Modulating Electrostatic Interactions in Ion Pair Intermediates To Alter Site Selectivity in the C–O Deoxygenation of Sugars. Angew. Chem., Int. Ed. 2020, 59 (39), 17297–17300. 10.1002/anie.202007415. 32521102

[ref33] Normand A. T.; Daniliuc C. G.; Wibbeling B.; Kehr G.; Le Gendre P.; Erker G. Phosphido- and Amidozirconocene Cation-Based Frustrated Lewis Pair Chemistry. J. Am. Chem. Soc. 2015, 137 (33), 10796–10808. 10.1021/jacs.5b06551. 26196212

[ref34] Brown H. C.; Kramer G. W.; Hubbard J. L.; Krishnamurthy S. Addition compounds of alkali metal hydrides : XVIII. Reaction of trialkylboranes with t-butyllithium. A general, convenient method for the preparation of lithium trialkylborohydride. J. Organomet. Chem. 1980, 188 (1), 1–10. 10.1016/S0022-328X(00)83693-1.

[ref35] Piers W. E.; Chivers T. Pentafluorophenylboranes: From Obscurity to Applications. Chem. Soc. Rev. 1997, 26 (5), 345–354. 10.1039/cs9972600345.

[ref36] Patrick E. A.; Piers W. E. Twenty-Five Years of Bis-Pentafluorophenyl Borane: A Versatile Reagent for Catalyst and Materials Synthesis. Chem. Commun. 2020, 56 (6), 841–853. 10.1039/C9CC08338C. 31872836

[ref37] Parks D. J.; von H.; Spence R. E.; Piers W. E. Bis(Pentafluorophenyl)Borane: Synthesis, Properties, and Hydroboration Chemistry of a Highly Electrophilic Borane Reagent. Angew Chem. Int. Ed. Engl. 1995, 34 (7), 809–811. 10.1002/anie.199508091.

[ref38] Parks D. J.; Piers W. E.; Yap G. P. A. Synthesis, Properties, and Hydroboration Activity of the Highly Electrophilic Borane Bis(Pentafluorophenyl)Borane, HB(C_6_F_5_)_2_. Organometallics 1998, 17 (25), 5492–5503. 10.1021/om980673e.

[ref39] Heiden Z. M.; Lathem A. P. Establishing the Hydride Donor Abilities of Main Group Hydrides. Organometallics 2015, 34 (10), 1818–1827. 10.1021/om5011512.

[ref40] Si G.; Tan C.; Chen M.; Chen C. A Cocatalyst Strategy to Enhance Ruthenium-Mediated Metathesis Reactivity towards Electron-Deficient Substrates. Angew. Chem., Int. Ed. 2022, 61 (29), e202203796 10.1002/anie.202203796. 35510712

[ref41] McKinty A. M.; Lund C.; Stephan D. W. A Tridentate-Dithiolate Ruthenium Alkylidene Complex: An Olefin Metathesis Catalyst Activated by BCl_3_. Organometallics 2013, 32 (17), 4730–4732. 10.1021/om400794u.

[ref42] Nieczypor P.; van Leeuwen P. W. N. M.; Mol J. C.; Lutz M.; Spek A. L. Synthesis, Structure, and Metathesis Activity of Ruthenium Carbene Complexes Containing Diphosphines. J. Organomet. Chem. 2001, 625 (1), 58–66. 10.1016/S0022-328X(00)00875-5.

[ref43] Walker R.; Conrad R. M.; Grubbs R. H. The Living ROMP of Trans-Cyclooctene. Macromolecules 2009, 42 (3), 599–605. 10.1021/ma801693q. 20379393 PMC2850575

