# Supplementary material for: B(C6F5)3 Co-Catalyst Promotes Unconventional Halide Abstraction from Grubbs I to Enhance Reactivity and Limit Decomposition
Source: Organometallics. 2024 Oct 23;43(21):2727–35. doi: 10.1021/acs.organomet.4c00178 (PMC11558663; doi:10.1021/acs.organomet.4c00178)
Supplement: Supplementary file 1 — om4c00178_si_001.pdf [file om4c00178_si_001.pdf]

Supporting Information

$B(C_6F_5)_3$ Co-Catalyst Promotes Unconventional Halide Abstraction from Grubbs I to Enhance Reactivity and Limit Decomposition

Austin W. Medley [a], Diya Patel [a‡], Calvin Utne[a‡], and Trandon A. Bender[a*]

a. Department of Chemistry and Biochemistry, Old Dominion University, 4501 Elkhorn Ave, Norfolk, VA 23529, USA.

*Corresponding Author E-mail: tbender@odu.edu

‡These authors contributed equally.

Table of Contents:

General Experimental Details S2

General Procedures S2-9

GI-DPPF ^{1}H NMR / $^{31}P\{^{1}H\}$ NMR Spectra S10-11

GI-Xantphos ^{1}H NMR / $^{31}P\{^{1}H\}$ NMR Spectra S11-12

Cp-BCF ^{1}H NMR / $^{19}F\{^{1}H\}$ NMR Spectra S13-14

Initial Optimization Table S14

- Initial Optimization NMR Spectrum S15
- Initial Optimization GC/MS Traces S15-22
- GI-DPPF GC/MS Traces S23-24
- GI-Xantphos GC/MS Traces S25-26

$^{31}P\{^{1}H\}$ NMR Speciation Spectra S27-29

$^{19}F\{^{1}H\}$ NMR Speciation Spectra S30-35

Analogous precedent BCF-Cl $^{19}F\{^{1}H\}$ NMR spectrum.[3] S36

In Situ $^{11}B\{^{1}H\}$ NMR Speciation Spectra S37-38

In Situ mass spectrometry of reaction mixture. S39

Conventional Catalytic Cycle and Proposed Modified Cycle S40

Kinetic Data S41

- Alkylidene Consumption Studies S41

Propagation / Conversion Studies S42-45

Polymer Characterization Data S46

Cyclopentene Control Data S47-48
Cyclopentene BCF Data S49-51
Cyclopentene H-BCF Data S52-54
Cyclopentene Cp-BCF Data S55-57
Cyclopentene NaBArF Data S58-59

Cyclooctene Control Data S60-62
Cyclooctene BCF Data S63-65
Cyclooctene H-BCF Data S66-67
Cyclooctene Cp-BCF Data S68-70
Cyclooctene NaBArF Data S71-72

Calibration Curve S73

NMR Characterization S74-87

Cyclopentene Control ^{1}H / ^{13}C NMR spectra S74-75
Cyclopentene BCF ^{1}H / ^{13}C NMR spectra S76
Cyclopentene H-BCF ^{1}H / ^{13}C NMR spectra S77
Cyclopentene Cp-BCF ^{1}H / ^{13}C NMR spectra S78-79
Cyclopentene NaBArF ^{1}H / ^{13}C NMR spectra S79-80

Cyclooctene Control Data S ^{1}H / ^{13}C NMR spectra S81-82
Cyclooctene BCF/**6** Data S ^{1}H / ^{13}C NMR spectra S82-83
Cyclooctene H-BCF/**6** Data S ^{1}H / ^{13}C NMR spectra S83-84
Cyclooctene Cp-BCF/**6** Data S ^{1}H / ^{13}C NMR spectra S85-86
Cyclooctene NaBArF/**6** ^{1}H / ^{13}C NMR spectra S87

References S88

General Experimental

General Methods: All reactions, unless otherwise stated, were carried out in oven (130°C) dried glassware under an inert atmosphere using standard Schlenk techniques. Unless otherwise specified, all reactions were conducted at ambient temperature (25°C, rt).

Chemicals: All reagents were used as received from commercial sources without further purification. All solvents, unless otherwise stated, were degassed with nitrogen for 30 minutes prior to use, after being passed through activated alumina columns in a solvent purification system.

NMR: All ^{1}H NMR and ^{13}C spectra were recorded on either a 400 MHz Bruker Avance spectrometer or 600 MHz Bruker Avance spectrometer. All deuterated solvents were used as received from Cambridge Isotope Laboratories, Inc. Chemical shifts are reported in parts

per million (ppm) with the residual solvent protons used as internal calibration standards.[1] The following abbreviations are used in reporting NMR data: s, singlet; d, doublet; t, triplet; q, quartet; quint, quintet; sept, septet; dd, doublet of doublets; dt, doublet of triplets; dq, doublet of quartets; td, triplet of doublets; tt, triplet of triplets; quint d, quintet of doublets; ddd, doublet of doublet of doublets; and m, multiplet.

Mass Spectrometry: High-resolution mass spectrometry data was obtained on a Bruker Daltonics 12T Apex Qe FTICR-MS using an Apollo II positive mode electrospray ionization (ESI) in picometer concentrations using MeCN as the solvent.

Gel Permeation Chromatography: Gel permeation chromatography data was collected using a Shimadzu refractive index detector, RID-20A and Wyatt miniDAWN multi-angle laser light scattering (MALLS) detector using Shodex GPC column KF-805L. Polymers were dissolved in 0.5 mL of THF and injected at a flow rate of 0.7 mL/min. The collected retention times and molecular weights were plotted on a calibration curve using known standards of polystyrene to obtain definitive molecular weights.

GC/MS: Gas chromatograph mass spectrometry data was collected on a Shimadzu GCMS-QP2010SE gas chromatograph mass spectrometer. Samples were diluted in 1.5 mL of hexanes and injected via split injection mode. Mass spectrometry data was collected in 0.2 second scans from 35 m/z to 700 m/z. Reaction conversion was monitored by GC/MS using toluene as an internal standard.

Thermometric Gravity Analysis: Thermometric gravity analysis data were collected on a TGA Q5000, instrument number 957001.901. Platinum TGA pans were first cleaned by blowtorch before they were tared, at which time 5 mg of polymer was loaded onto them.

Differential Scanning Calorimetry: Differential scanning calorimetry data were obtained on a TA Q20 V24.11. The general method included a 30°C/min ramp, beginning at -40°C and ending at 300°C. Glass transition states (Tg) were determined by integrating peaks as a function of heat flow (W/g) and temperature (°C). All samples were checked by thermogravimetric analysis prior to use. Samples were in the mass range between 2-11 mg, with each of which pressed into standard 5.4mm aluminum tzero pans.

Piers-Borane Synthetic Procedure

Piers-Borane was prepared according to known literature procedure. [2] To a 20 mL scintillation vial charged with a stir bar was added tris(pentafluoro)phenylborane (500 mg, 0.98 mmol) and this was dissolved in benzene (10 mL). To this triethylsilane (0.12 g, 0.99 mmol) was added. The vial was capped, brough to 60 ^{o}C, and allowed to stir for 3 days. The solution was quickly filtered over a frit and the supernatant collected. The material began crashing out of solution and was collected on by filtration. Volatiles were removed to yield a white solid, 0.22 g, 68% in pure form. Spectra matched reported.

GI-DPPF Synthetic Procedure

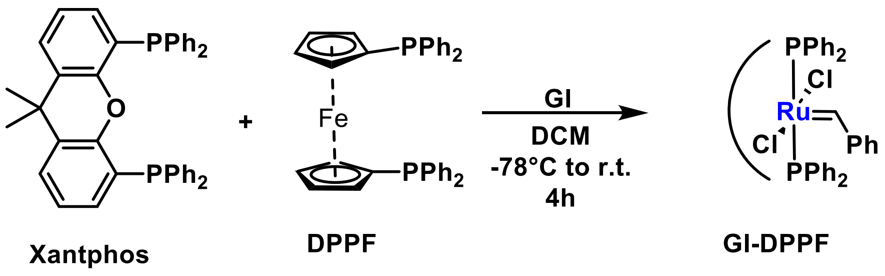

Scheme S1. Synthetic procedure for the synthesis of GI-DPPF.

GI-DPPF was prepared according to literature procedure.[4] A solution of 100 mg GI (0.12 mmol, 1 eq) in 15 mL of DCM cooled to -78°C. To this solution was added 67 mg of DPPF (0.12 mmol, 1 eq) in 5 mL DCM. The reaction was stirred at -78°C for 20 minutes and then allowed to warm to room temperature. After another hour, the reaction volume was reduced by half under reduced atmosphere. 15 mL of pentane was then added to the reaction mixture to precipitate a green solid. The solid was isolated and reprecipitated twice out of DCM/pentane and dried in vacuo to yield green-brown product GI-DPPF in 40% yield (39 mg, 0.048 mmol). Spectra matched that which was previously reported in the chemical literature.[4]

GI-Xantphos Synthetic Procedure

GI-Xantphos was prepared according to literature procedure.[4] A solution of 100 mg GI (0.12 mmol, 1 eq) in 15 mL of DCM cooled to -78°C. To this solution was added 69 mg of DPPF (0.12 mmol, 1 eq) in 5 mL DCM. The reaction was stirred at -78°C for 20 minutes and then allowed to warm to room temperature. After another hour, the reaction volume was reduced by half under reduced atmosphere. 15 mL of pentane was then added to the reaction mixture to precipitate a green solid. The solid was isolated and reprecipitated twice out of DCM/pentane and dried in vacuo to yield green-brown product GI-DPPF in 40% yield (40 mg, 0.048 mmol). Spectra matched that which was previously reported in the chemical literature.[4]

Cp-BCF Synthetic Procedure

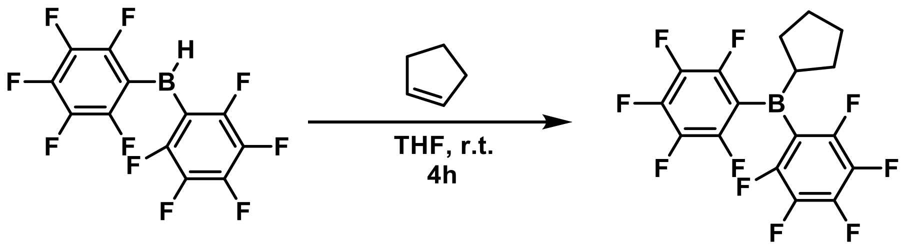

Scheme S2. Synthetic procedure for the synthesis of Cp-BCF.

To a 20 mL scintillation vial charged with a stir bar was added 100 mg H-BCF (0.29 mmol, 1 eq) and 0.300 L of cyclopentene (2.86 mmol, 9.9 eq). The mixture was diluted to 5 mL with THF and stirred for 4 hours at room temperature. Solvent and excess cyclopentene were removed in vacuo to afford 81 mg of Cp-BCF in 68% yield, without the need of further purification.

1H NMR (400 MHz, $CDCl_3$) δ 2.31 (m, J = 7.0 Hz, 1H), 1.83 (q, J = 5.9 Hz, 1H), 1.58 (m, J = 3.5 Hz, 1H), 1.26 (t, J = 8.1 Hz, 1H).

$^{19}F\{^1H\}$ NMR (376 MHz, $CDCl_3$) δ -130.44 (q, J = 10.7 Hz, 2F), -152.39 (d, J = 20.4 Hz, 1F), -161.92 (m, J = 11.2 Hz, 2F).

General Experimental Procedures

General synthetic procedure for control metathesis reactions.

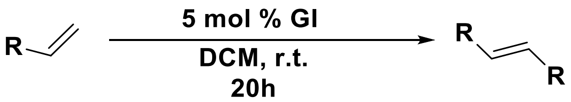

Scheme S3. General control reaction using 5 mol% of Grubbs 1.

Reactions were performed within an inert atmosphere glove box in 1 dram screw capped vials. To those vials, affixed with screw-capped pressure septa, was added 4.1 mg of Grubbs 1 (0.005 mmol) and 500 μL of DCM. Dry and degassed decene (0.1 mmol, 18.9 μL) were injected via μL syringe and allowed to stir at room temperature for 20h. Reactions were quenched by exposing them to air, concentrating under reduced atmosphere, followed by addition of $CDCl_3$. 7.91 μL of toluene was then added to the reaction as an internal standard for GC/MS and NMR characterization.

General synthetic procedure for cross-metathesis reactions.

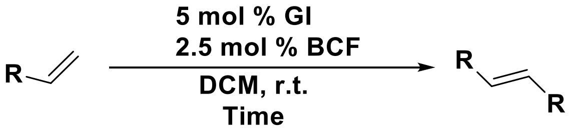

Scheme S4. General reaction using 5 mol% Grubbs 1 and 2.5 mol% BCF

Reactions were performed within an inert atmosphere glove box in 1 dram screw capped vials. To those vials, affixed with screw-capped pressure septa, was added 4.1 mg Grubbs 1 (0.005 mmol, 0.005 eq), 1.3 mg tris(pentafluoro)phenylborane (BCF) (0.0025 mmol, 0.0025 eq), and 500 μL of DCM. After 15 minutes to allow for abstraction to occur, dry and degassed decene was added via μL syringe (0.1 mmol, 18.9 μL) and allowed to stir at room temperature for 4h. Reactions were quenched by exposing them to air, concentration under reduced atmosphere followed by addition of $CDCl_3$ for characterization. 7.91 μL of toluene was then added to the reaction as an internal standard for GC/MS and NMR characterization.

General synthetic procedure for ROMP reactions.

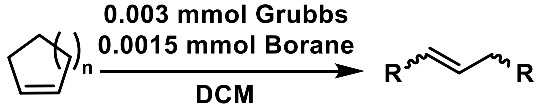

Scheme S5. General ROMP reaction with 0.003 mmol of Grubbs 1 and 0.0015 mmol of borane.

To a 1-dram screw capped vial, affixed with screw-capped pressure septa, was added 2.4 mg Grubbs I (0.003 mmol, 1 eq) and 0.7 mg tris(pentafluoro)phenylborane (BCF) (0.0015 mmol, 0.5 eq) and 500 μL of DCM. After 15 minutes to allow for abstraction to occur, to these solutions were added their respective monomer (1.8 mmol, 600 eq). The reaction was allowed to react for 20h time, at which point the solvent was removed under reduced atmosphere. These polymers were weighed directly, and GPC samples were directly prepared from these materials.

General synthetic procedure for Cl abstraction studies.

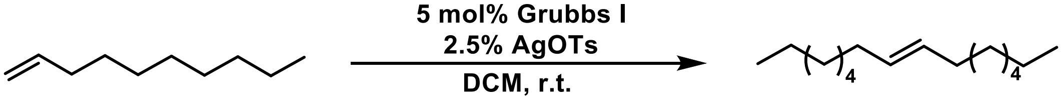

Scheme S6. General reaction for AgOTs halide abstraction activation of Grubbs 1 in cross-metathesis.

To a 1-dram vial was added 4.1 mg Grubbs I (0.005 mmol, 0.005 eq) and 0.7 mg AgOTs (0.0025 mmol, 0.0025 eq). The contents were diluted with 500 μL of CH_2Cl_2. After 15 minutes, to allow for abstraction to occur to this solution

was added 18.9 μL of decene (0.1 mmol, 1 eq) via syringe. The solution changed color from purple to dark brown rapidly and formed large amounts of precipitate. After 4 hours, an aliquot was removed for GC/MS analysis.

General synthetic procedure for NaBArF isomerization study.

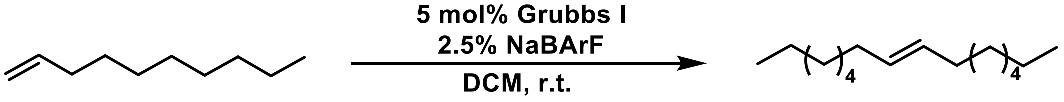

Scheme S7. General reaction for NaBArF activated Grubbs 1 in cross-metathesis.

To a 1-dram vial was added 4.1 mg of Grubbs I (0.005 mmol, 0.05 eq) and 2.2 mg NaBArF (0.0025 mmol, 0.025 eq). The contents were diluted with 500 μL of CH_2Cl_2. After 15 minutes to allow for abstraction to occur to this solution was added 18.9 μL of decene, (0.1 mmol, 1 eq) via syringe. The solution was allowed to stir for 4h at which time an aliquot was removed and diluted to 1 mL in hexanes for GC/MS analysis.

General synthetic procedure for NaBArF ROMP reaction of COE.

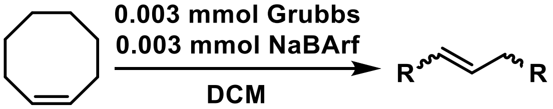

Scheme S8. General ROMP conditions for NaBArF activated Grubbs 1 in reaction with COE.

To a 1-dram vial was added 2.4 mg Grubbs I (0.003 mmol, 1 eq) and 2.7 mg NaBArF (0.003 mmol, 1 eq). This solution was diluted to 500 μL with CH_2Cl_2. After 15 minutes to allow for abstraction to occur, to this sample was injected 0.023 mL of cyclooctene (1.8 mmol). The reaction was allowed to stir for 4 hours at which point 2 mL of ethyl vinyl ether was added to quench it. The reaction was concentrated under reduced atmosphere and filtered prior to analysis. These polymers were weighed directly, and GPC samples were directly prepared from these materials.

General synthetic procedure for NaBArF ROMP reaction of Cp.

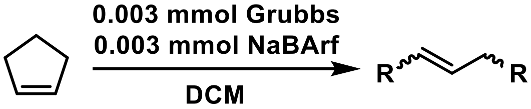

Scheme S9. General ROMP conditions for NaBArF activated Grubbs 1 in reaction with Cp.

To a 1-dram vial was added 2.4 mg Grubbs I (0.003 mmol, 1 eq) and 2.7 mg NaBArF (0.003 mmol, 1 eq). This solution was diluted to 500 μL with CH_2Cl_2. After 15 minutes to allow for abstraction to occur, to this sample was injected 0.09 mL of cyclopentene (1.8 mmol). The reaction was allowed to stir for 4 hours at which point 2 mL of ethyl vinyl ether was added to quench it. The reaction was concentrated under reduced atmosphere and filtered prior to analysis. These polymers were weighed directly, and GPC samples were directly prepared from these materials.

General synthetic procedure for $^{31}P\{^1H\}$ speciation studies.

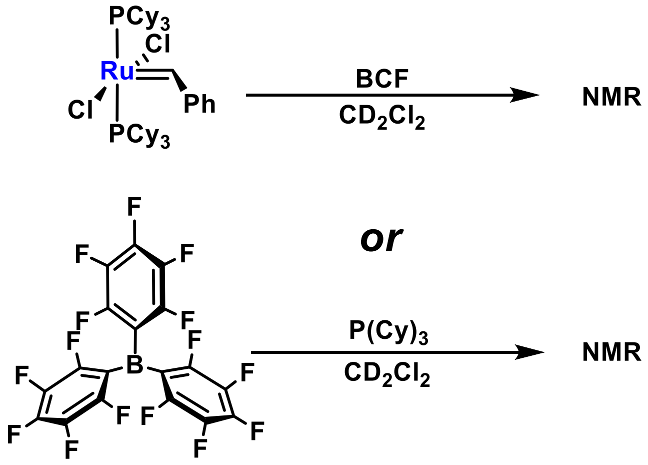

Scheme S10. Figure denoting the two $^{31}P\{^1H\}$ NMR experiments to examine phosphine speciation.

To a 1-dram vial was added either 2.4 mg of Grubbs 1 (0.003 mmol, 1 eq) and 0.7 mg BCF (0.0015 mmol, 0.5 eq) or 0.7 mg BCF (0.0015 mmol, 1 eq) and 0.4 mg $P(Cy)_3$ (0.0015 mmol, 1 eq). The contents of each were diluted with 500 uL of CD_2Cl_2 and transferred to NMR tubes. The tubes were then capped with rubber NMR septa and wrapped with parafilm. Spectra were taken without delay and compared to an authentic sample of Grubbs 1 in CD_2Cl_2.

General synthetic procedure for $^{31}P\{^1H\}$ NMR speciation dimer study.

To a 1-dram vial was added either 2.4 mg of Grubbs I (0.003 mmol, 1 eq) and 0.7 mg BCF (0.0015 mmol, 0.5 eq) or 0.7 mg BCF (0.0015 mmol, 1 eq) and 0.4 mg $P(Cy)_3$ (0.0015 mmol, 1 eq). The contents of each were diluted with 500 μL of CD_2Cl_2 and transferred to NMR tubes. The tubes were then capped with rubber NMR septa and wrapped with parafilm. Spectra were taken without delay and compared to an authentic sample of Grubbs 1 in CD_2Cl_2.

Procedure for $^{31}P\{^1H\}$ NMR quantification.

To a 1-dram vial was added 4.1 mg of Grubbs 1 (0.005 mmol, 1 eq) and a sealed 10 uL capillary of PPh3 in CH_2Cl_2 (130 mg, 0.5 mmol per 500 uL). The contents were diluted with 500 uL of CD_2Cl_2 and transferred to an NMR tube. The tube was then capped with rubber NMR septum and wrapped with parafilm. Spectra were taken without delay.

To a 1-dram vial was added 4.1 mg of Grubbs 1 (0.005 mmol, 1 eq), 1.3 mg tris(pentafluoro)phenylborane (BCF) (0.0025 mmol, 0.05 eq), and a sealed 10 uL capillary of PPh_3 in CH_2Cl_2 (130 mg, 0.5 mmol per 500 uL). The contents were diluted with 500 uL of CD_2Cl_2 and transferred to an NMR tube. The tube was then capped with rubber NMR septum and wrapped with parafilm. Spectra were taken without delay.

General synthetic procedure for ^{19}F speciation studies.

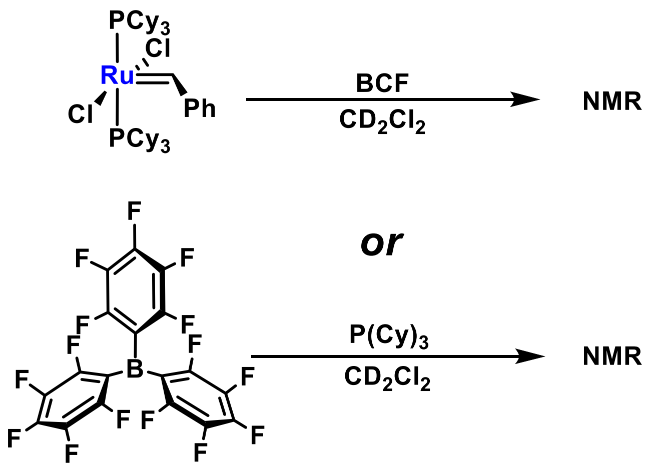

Scheme S11. Figure denoting the two ^{19}F NMR experiments to examine phosphine speciation.

To a 1-dram vial was added either 2.4 mg of Grubbs I (0.003 mmol, 1 eq) and 0.7 mg BCF (0.0015 mmol, 0.5 eq) or 0.7 mg BCF (0.0015 mmol, 1 eq) and 0.4 mg $P(Cy)_3$ (0.0015 mmol, 1 eq). The contents of each were diluted with 500 μL of CD_2Cl_2 and transferred to NMR tubes. The tubes were then capped with rubber NMR septum and wrapped with parafilm. Spectra were taken without further delay and compared to an authentic sample of Grubbs 1 in CD_2Cl_2.

Authentic BCF-Cl $^{19}F\{^1H\}$ NMR Speciation Study.

To a 1-dram vial was added 3.9 mg of $[IrCp^*Cl]_2$ (0.005 mmol, 1 eq) and 1.3 mg tris(pentafluoro)phenylborane (BCF) (0.0025 mmol, 0.5 eq). The reaction was diluted to 500 uL with CD_2Cl_2 and transferred to an NMR tube. The reaction was capped with a rubber septum and wrapped with parafilm. $^{19}F\{^1H\}$ NMR spectra were taken without delay and clean conversion of BCF to its corresponding BCF-Cl species was observed.

General control procedure for Grubbs 1 alkylidene consumption study.

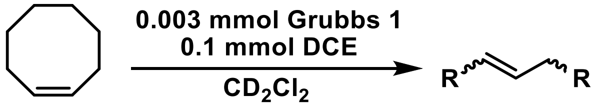

Scheme S12. General NMR control reaction for Grubbs 1 kinetics studies.

To a 1-dram vial was added 2.4 mg Grubbs I (0.003 mmol, 1 eq) and 7.91 μL of dichloroethane (DCE) (0.1 mmol). This solution was diluted to 500 μL with CD_2Cl_2, at which time it was transferred to an NMR tube, capped with a rubber septum, and wrapped with parafilm. The sample was injected into a 600 MHz NMR to examine the alkylidene shift. Once the parent spectra were obtained, the sample was re-ejected from the probe and 23.3 μL of cyclooctene (1.8 mmol) was added via syringe through the septum. The sample was quickly reinjected into the probe and NMR time points were taken. Alkylidene consumption was observed qualitatively, as such further propagation kinetic studies were performed.

General synthetic procedure for Grubbs 1 alkylidene consumption study.

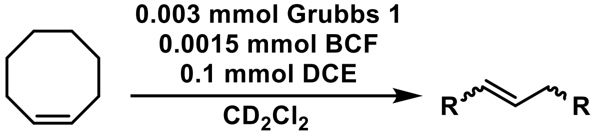

Scheme S13. General NMR control reaction for Grubbs 1 kinetics studies.

To a 1-dram vial was added 2.4 mg Grubbs 1 (0.003 mmol, 1 eq), 0.7 mg BCF (0.0015 mmol, 0.5 eq) and 7.91 μL of DCE (0.1 mmol). This solution was diluted to 500 μL with CD_2Cl_2, at which time it was transferred to an NMR tube, capped with a rubber septum, and wrapped with parafilm. The sample was injected into a 600 MHz NMR to examine the alkylidene shift. Once the parent spectra were obtained, the sample was re-ejected from the probe and 23.3 μL of cyclooctene (1.8 mmol) was added via syringe through the septum. The sample was quickly reinjected into the probe and NMR time points were taken. Substrate consumption was integrated with respect to DCE as an internal standard.

General control procedure for Grubbs 1 kinetic study.

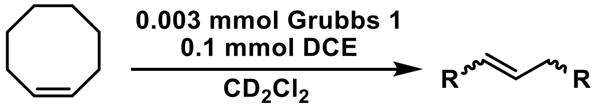

Scheme S14. General NMR control reaction for Grubbs 1 kinetics studies.

To a 1-dram vial was added 2.4 mg Grubbs 1 (0.003 mmol, 1 eq) and 7.91 μL of dichloroethane (DCE) (0.1 mmol). This solution was diluted to 500 μL with CD_2Cl_2, at which time it was transferred to an NMR tube, capped with a rubber septum, and wrapped with parafilm. The sample was injected into a 600 MHz NMR to examine the alkylidene shift. Once the parent spectra were obtained, the sample was re-ejected from the probe and 23.3 μL of cyclooctene (1.8 mmol) was added via syringe through the septum. The sample was quickly reinjected into the probe and NMR time points were taken. Substrate consumption was integrated with respect to DCE as an internal standard.

General synthetic procedure for Grubbs 1/BCF kinetic study.

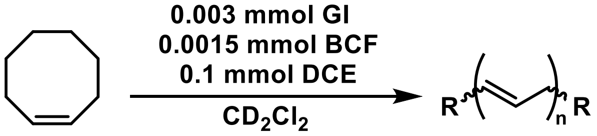

Scheme S15. General NMR reaction for Grubbs 1 kinetics studies.

To a 1-dram vial was added 2.4 mg Grubbs 1 (0.003 mmol, 1 eq), 0.7 mg BCF (0.0015 mmol, 0.5 eq) and 7.91 μL of DCE (0.1 mmol). This solution was diluted to 500 μL with CD_2Cl_2, at which time it was transferred to an NMR tube, capped with a rubber septum, and wrapped with parafilm. The sample was injected into a 600 MHz NMR to examine the alkylidene shift. Once the parent spectra were obtained, the sample was re-ejected from the probe and 23.3 μL of cyclooctene (1.8 mmol) was added via syringe through the septum. The sample was quickly reinjected into the probe and NMR time points were taken. Substrate consumption was integrated with respect to DCE as an internal standard.

General synthetic procedure for Grubbs 2 kinetic study.

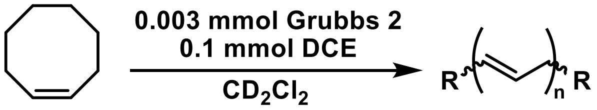

Scheme S16. General NMR control reaction for Grubbs 2 kinetics studies.

To a 1-dram vial was added 2.5 mg Grubbs IIs (0.003 mmol, 1 eq) and 7.91 μL of DCE (0.1 mmol). This solution was diluted to 500 μL with CD_2Cl_2, at which time it was transferred to an NMR tube, capped with a rubber septum, and wrapped with parafilm. The sample was injected into a 600 mHz NMR. Once the parent spectra were obtained, the sample was re-ejected from the probe and 23.3 μL of cyclooctene (1.8 mmol) was added via syringe through the

septum. The sample was quickly reinjected into the probe and NMR time points were taken. Substrate consumption was integrated with respect to DCE as an internal standard.

GI-DPPF ^{1}H NMR Spectra

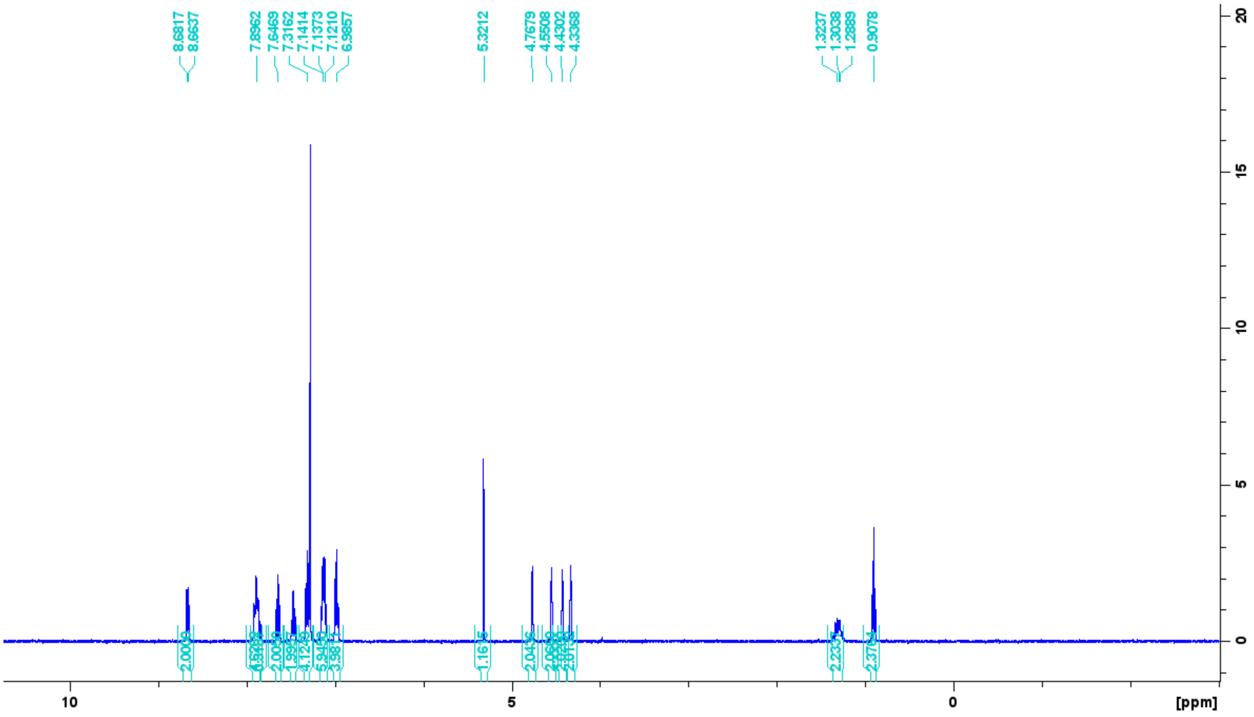

Figure S1. ^{1}H NMR spectrum of GI-DPPF in $CDCl_3$. Spectrum matched literature.[4]

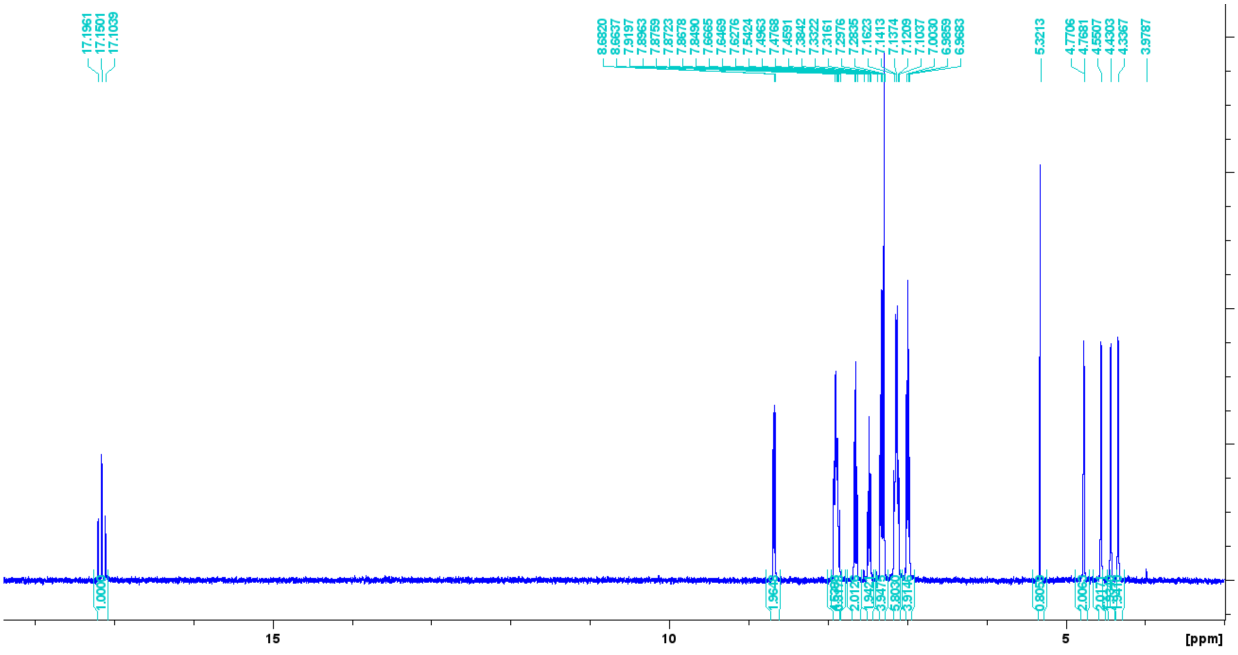

Figure S2. Downfield ^{1}H NMR spectrum of GI-DPPF in $CDCl_3$. Spectrum matched literature.[4]

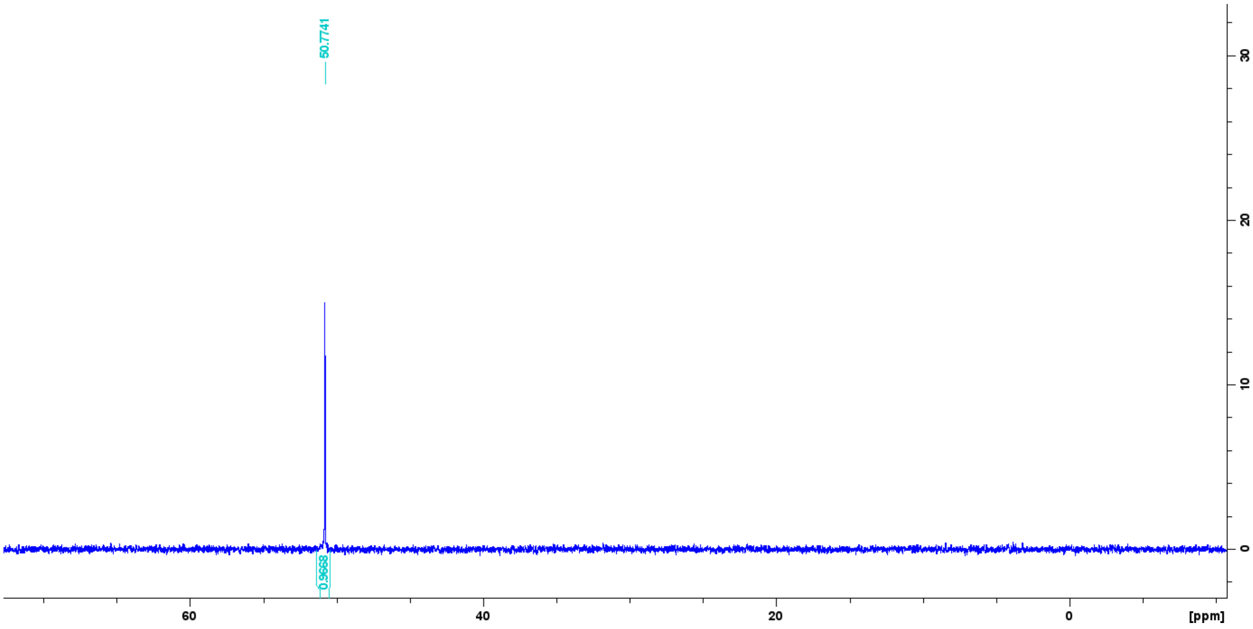

Figure S3. ^{31}P NMR spectrum of GI-DPPF in $CDCl_3$. Spectrum matched literature.[4]

GI-Xantphos ^{1}H NMR Spectra

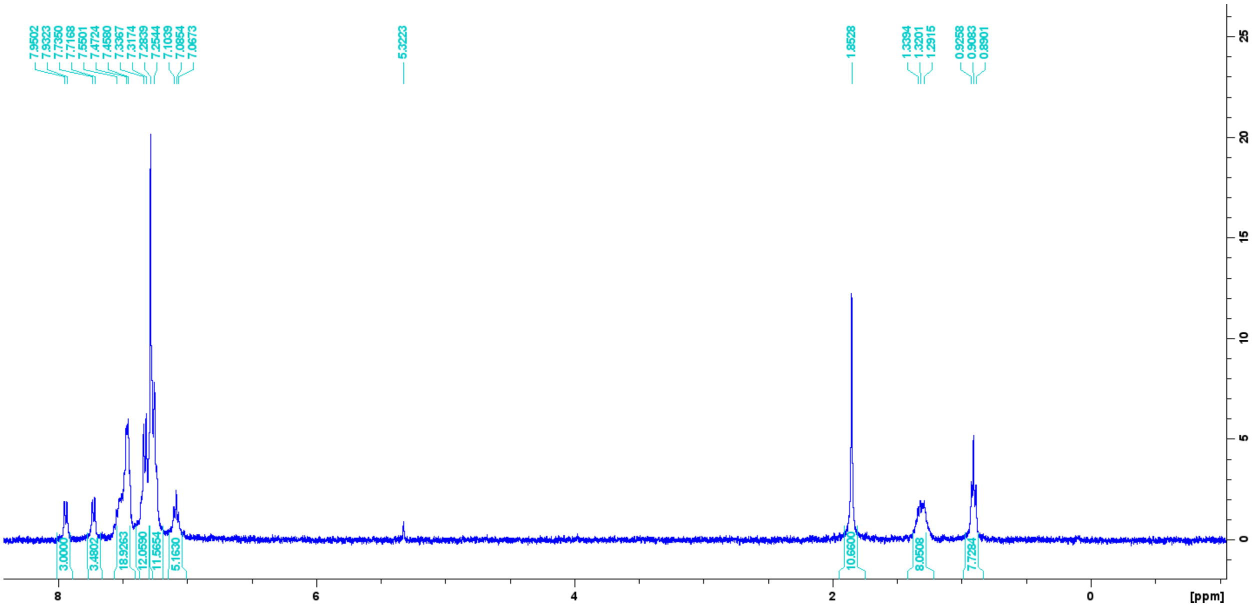

Figure S4. ^{1}H NMR spectrum of GI-DPPF in $CDCl_3$. Spectrum matched literature.[4]

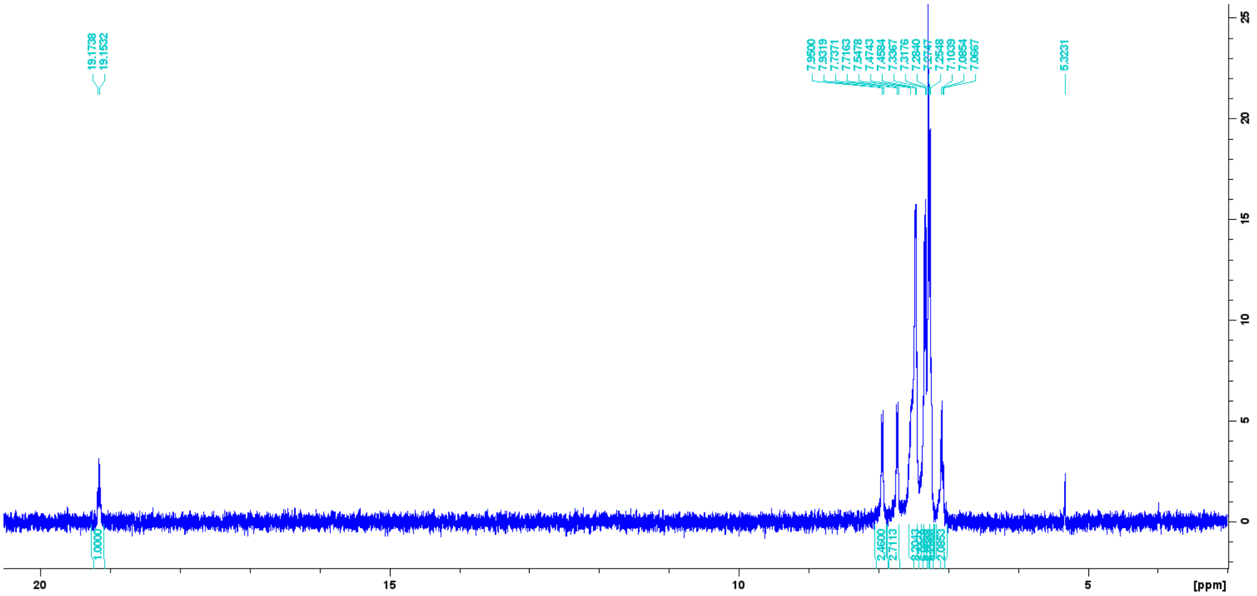

Figure S5. Downfield ^1H NMR spectrum of GI-Xantphos in $CDCl_3$. Spectrum matched literature.[4]

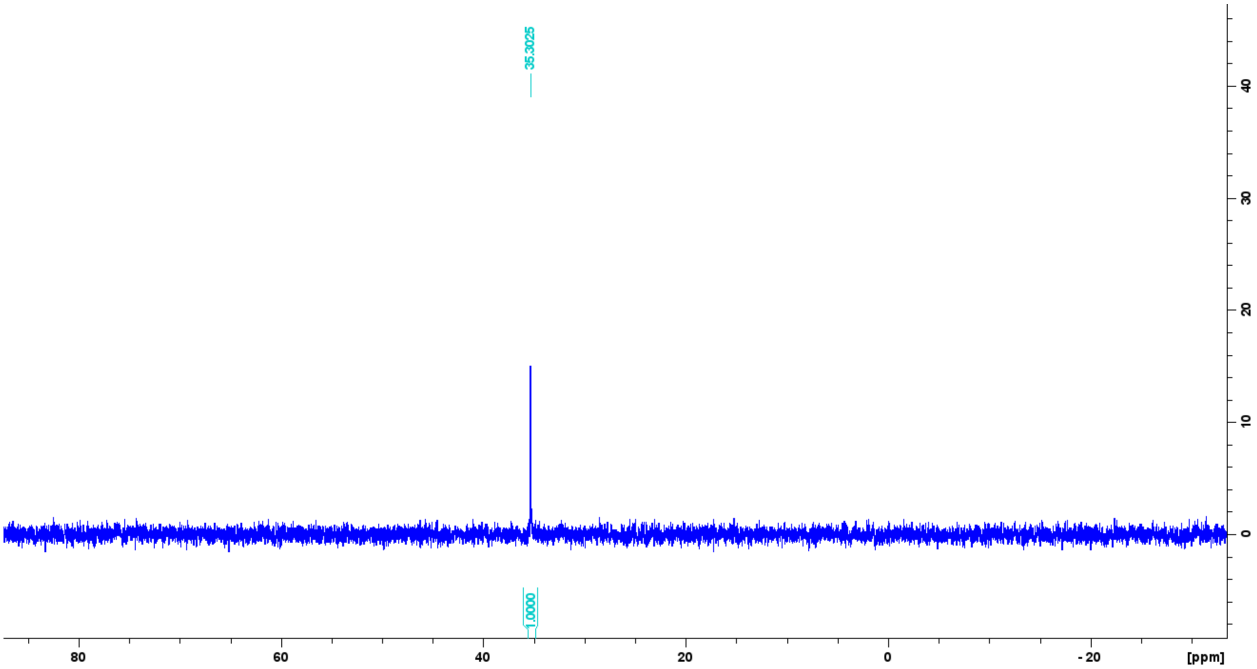

Figure S6. ^{31}P NMR spectrum of GI-Xantphos in $CDCl_3$. Spectrum matched literature.[4]

Cp-BCF ^{1}H NMR / $^{19}F\{^{1}H\}$ NMR spectra

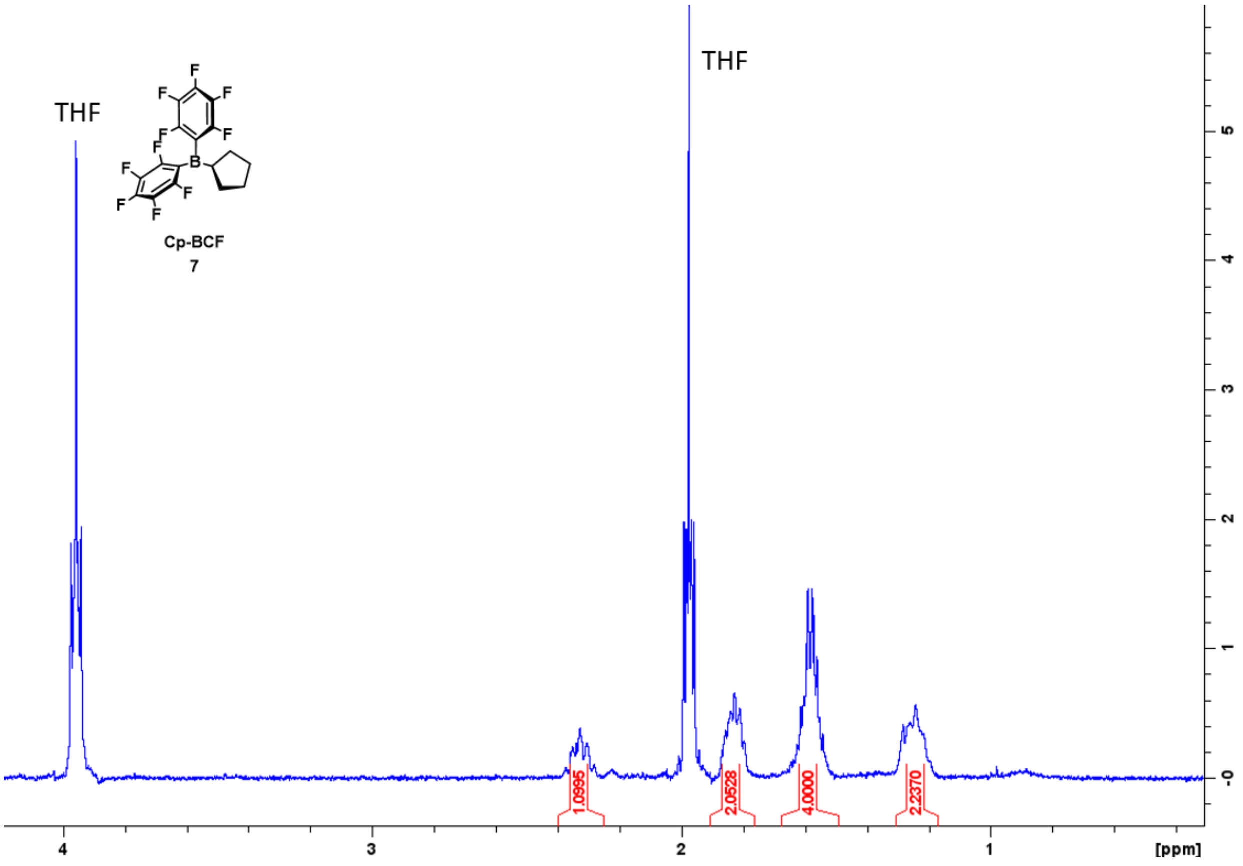

Figure S7. Cp-BCF ^{1}H NMR spectrum in $CDCl_3$.

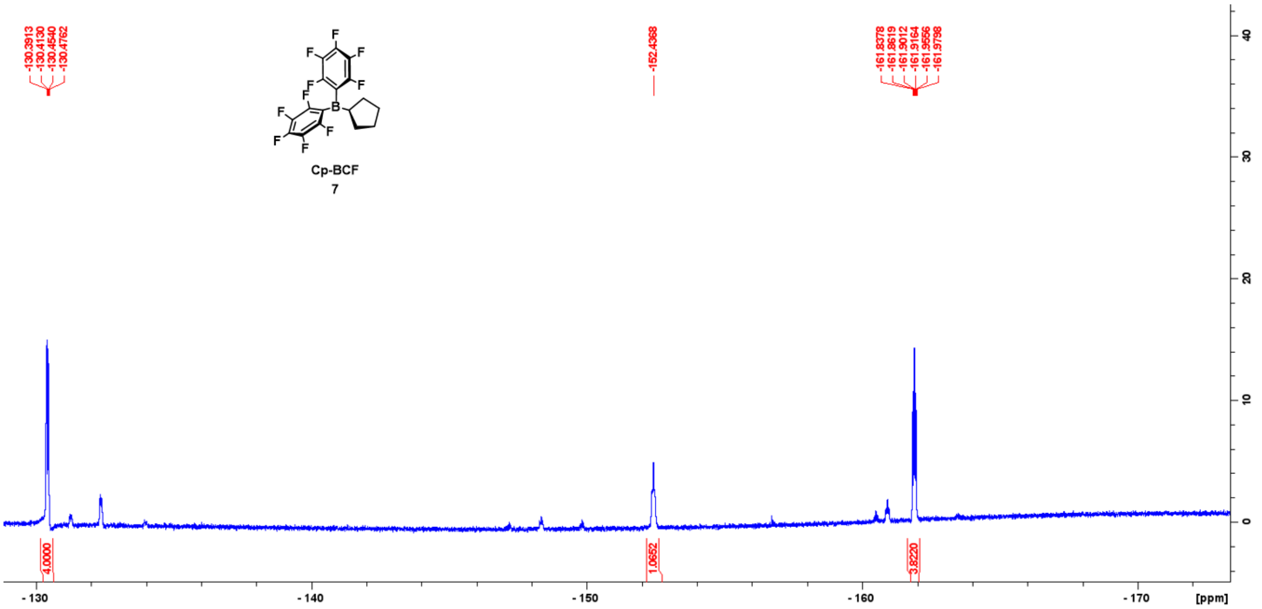

Figure S8. Cp-BCF $^{19}F\{^{1}H\}$ NMR spectrum in $CDCl_3$.

Table S1. Expanded Initial Screening Data

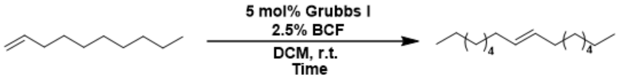

Entry	Substrate	Grubbs (mol%)	BCF (mol%)	Solvent	Additive	Conversion (%)[a]	% Isomerization[a]	Time (h)
1	1-decene	5	0	DCM	N/A	65	13	20
2	1-decene	5	2.5	DCM	N/A	99	6	0.5
3	1-decene	5	2.5	CDCl3	N/A	ND	ND	4
4	1-decene	5	5	DCM	N/A	77	49	4
5	1-decene	5	5	DCM	PCy3	ND	ND	4
6	1-decene	0	2.5	DCM	N/A	NR	NR	4
7	1-decene	5	0	DCM	NaBArF	70	35	4
8	1-decene	5	0	DCM	AgOTs	52	77	4
9	1-decene	5	10	DCM	N/A	99	ND	4
10	1-decene	5	0	DCM	Boric Acid	55	trace	4

Table S1. Initial screening data of reaction optimization with decene. a. Determined by GC-MS analysis with toluene as an internal standard.

Entry 1. ^{1}H NMR Conversion Study (Grubbs 1 only)

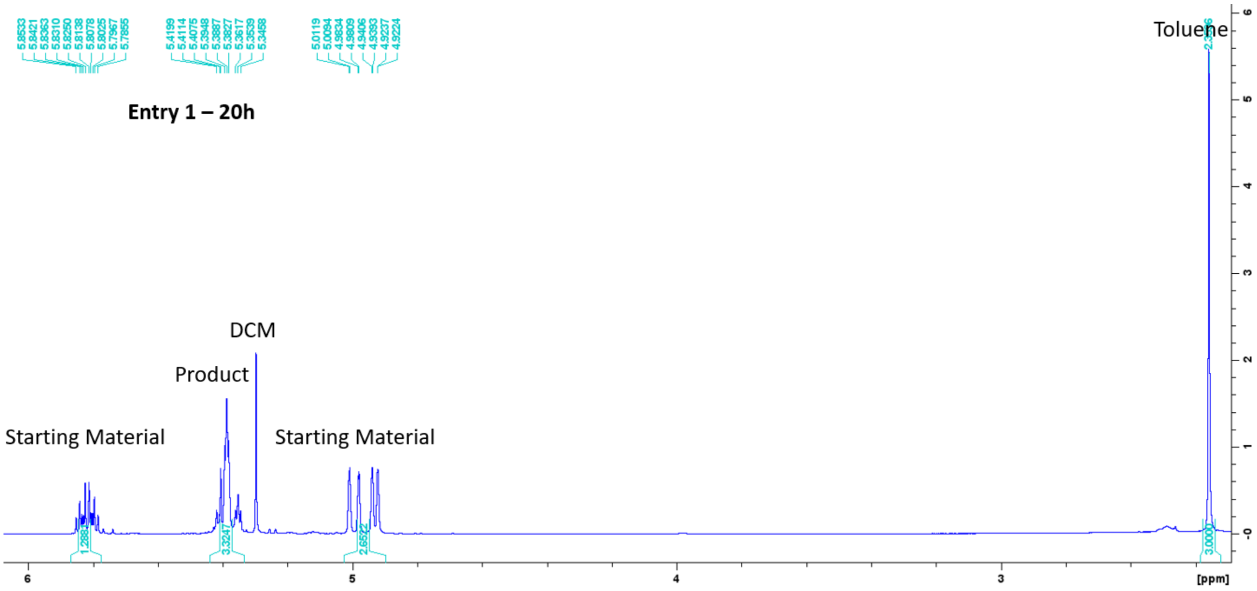

Figure S9. 20h ¹H NMR spectrum conversion of Entry 1 with toluene as an internal standard.

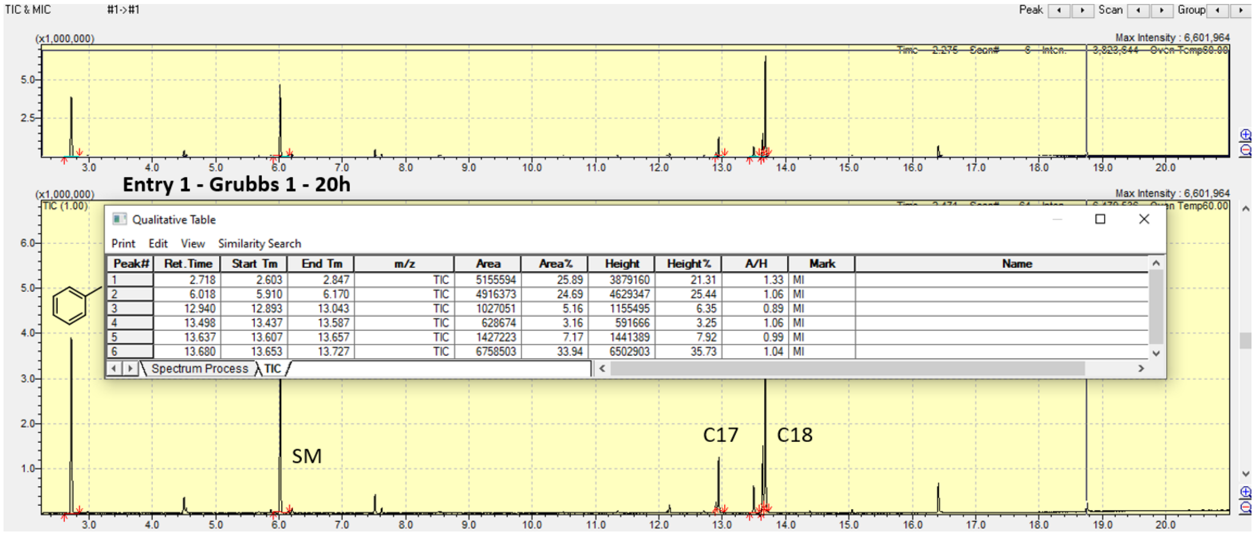

Peak#	Ret.Time	Start Tm	End Tm	m/z	Area	Area%	Height	Height%	A/H	Mark	Name
1	2.718	2.603	2.847	TIC	5155594	25.89	3879160	21.31	1.33	MI	
2	6.018	5.910	6.170	TIC	4916373	24.69	4629347	25.44	1.06	MI	
3	12.940	12.893	13.043	TIC	1027051	5.16	1155495	6.35	0.89	MI	
4	13.498	13.437	13.587	TIC	628674	3.16	591666	3.25	1.06	MI	
5	13.637	13.607	13.657	TIC	1427223	7.17	1441389	7.92	0.99	MI	
6	13.680	13.653	13.727	TIC	6758503	33.94	6502903	35.73	1.04	MI	

Figure S10. 20h GC/MS trace conversion of Entry 1 with toluene as an internal standard.

Entry 2a: (Grubbs 1 / BCF) Optimized GC/MS Trace at 30 Minutes

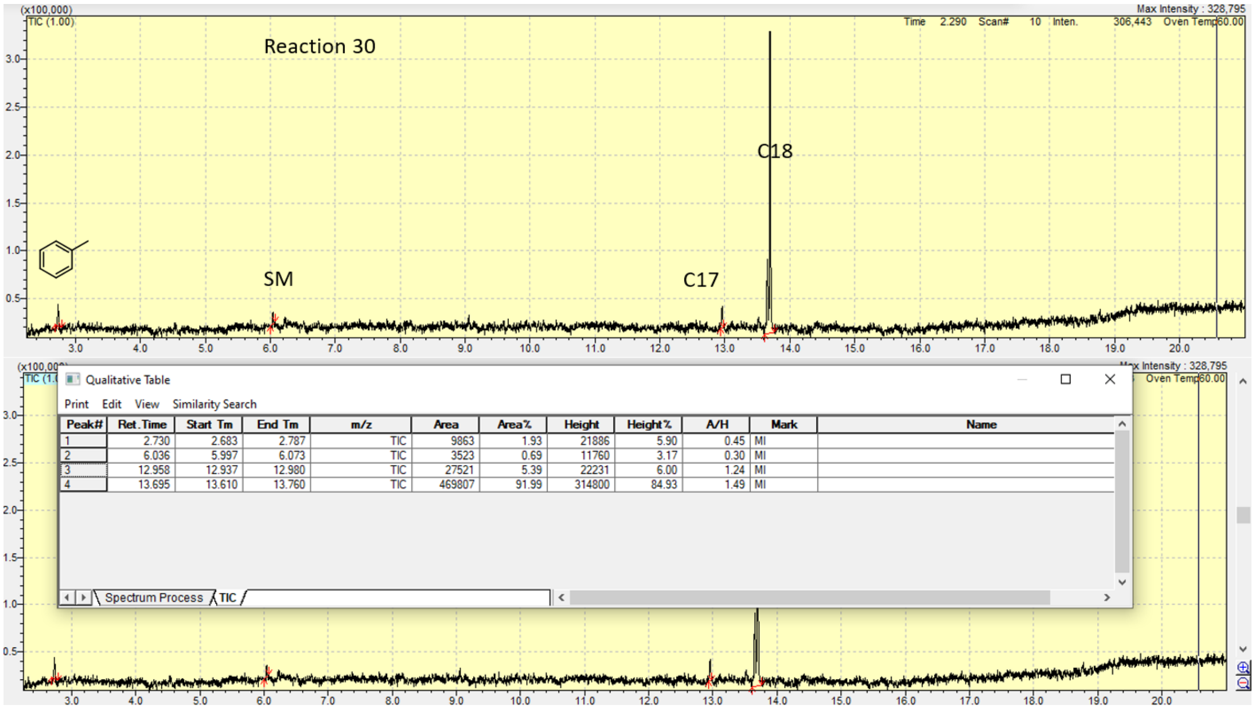

Peak#	Ret.Time	Start Tm	End Tm	m/z	Area	Area%	Height	Height%	A/H	Mark	Name
1	2.730	2.683	2.787	TIC	9863	1.93	21886	5.90	0.45	MI	
2	6.036	5.997	6.073	TIC	3523	0.69	11760	3.17	0.30	MI	
3	12.958	12.937	12.980	TIC	27521	5.39	22231	6.00	1.24	MI	
4	13.695	13.610	13.760	TIC	469807	91.99	314800	84.93	1.49	MI	

Figure S11. 30-minute aliquot of optimized G1/BCF reaction by GC/MS with toluene as an internal standard.

Entry 2b: (Grubbs 1 / BCF) Optimized GC/MS Trace at 60 Minutes

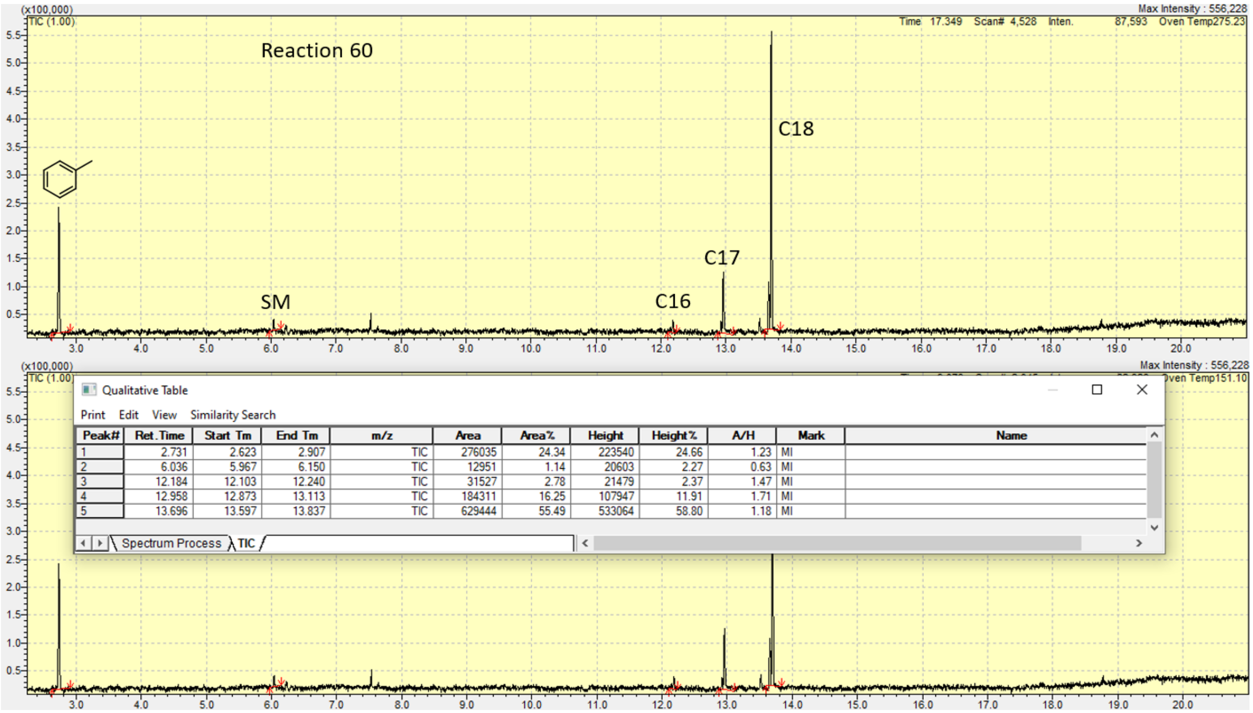

Peak#	Ret.Time	Start Tm	End Tm	m/z	Area	Area%	Height	Height%	A/H	Mark	Name
1	2.731	2.623	2.907	TIC	276035	24.34	223540	24.66	1.23	MI	
2	6.036	5.967	6.150	TIC	12951	1.14	20603	2.27	0.63	MI	
3	12.184	12.103	12.240	TIC	31527	2.78	21479	2.37	1.47	MI	
4	12.958	12.873	13.113	TIC	184311	16.25	107947	11.91	1.71	MI	
5	13.696	13.597	13.837	TIC	629444	55.49	533064	58.80	1.18	MI	

Figure S12. 60-minute aliquot of optimized G1/BCF reaction by GC/MS with toluene as an internal standard.

Entry 2c: (Grubbs 1 / BCF) Optimized GC/MS Trace at 90 Minutes

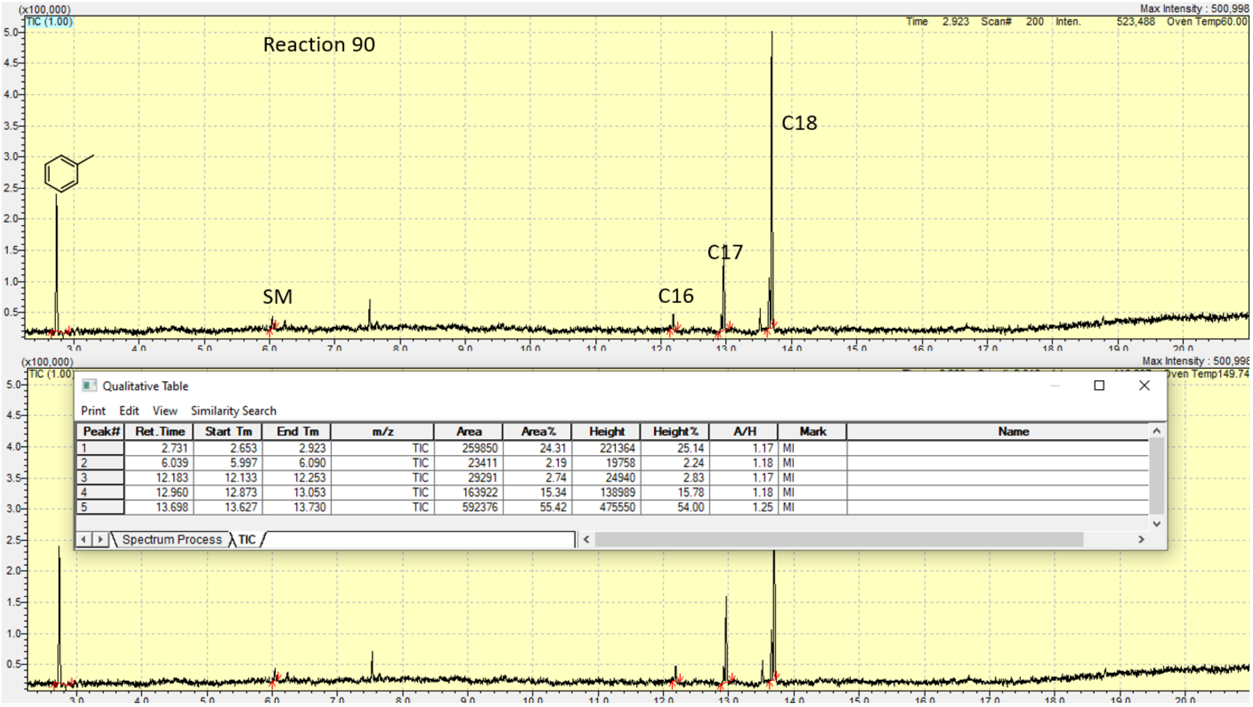

Peak#	Ret.Time	Start Tm	End Tm	m/z	Area	Area%	Height	Height%	A/H	Mark	Name
1	2.731	2.653	2.923	TIC	259850	24.31	221364	25.14	1.17	MI	
2	6.039	5.997	6.090	TIC	23411	2.19	19758	2.24	1.18	MI	
3	12.183	12.133	12.253	TIC	29291	2.74	24940	2.83	1.17	MI	
4	12.960	12.873	13.053	TIC	163922	15.34	138989	15.78	1.18	MI	
5	13.698	13.627	13.730	TIC	592376	55.42	475550	54.00	1.25	MI	

Figure S13. 90-minute aliquot of optimized G1/BCF reaction by GC/MS with toluene as an internal standard.

Entry 2d: (Grubbs 1 / BCF) Optimized GC/MS Trace at 210 Minutes

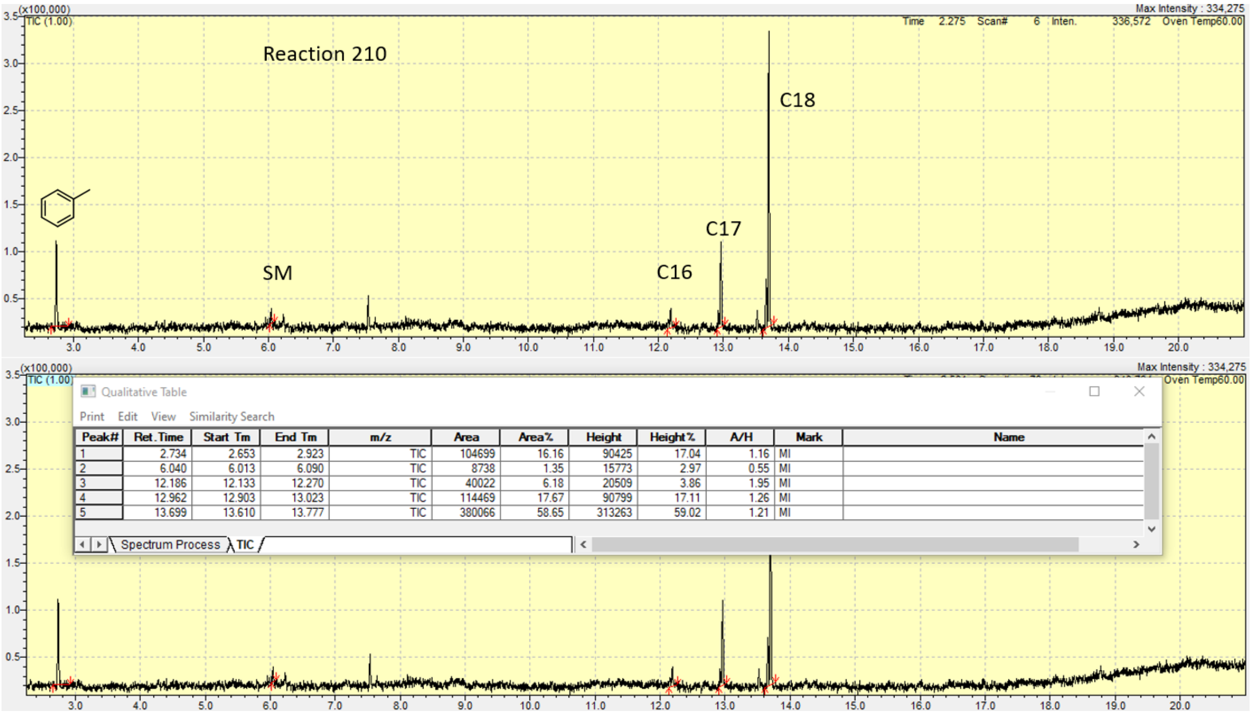

Peak#	Ret.Time	Start Tm	End Tm	m/z	Area	Area%	Height	Height%	A/H	Mark	Name
1	2.734	2.653	2.923	TIC	104699	16.16	90425	17.04	1.16	MI	
2	6.040	6.013	6.090	TIC	8738	1.35	15773	2.97	0.55	MI	
3	12.186	12.133	12.270	TIC	40022	6.18	20509	3.86	1.95	MI	
4	12.962	12.903	13.023	TIC	114469	17.67	90799	17.11	1.26	MI	
5	13.699	13.610	13.777	TIC	380066	58.65	313263	59.02	1.21	MI	

Figure S14. 210-minute aliquot of optimized G1/BCF reaction by GC/MS with toluene as an internal standard.

** As shown, the combination of G1/BCF rapidly converts decene to its corresponding C18 product with little isomerization, but as time progresses will continue to react and do isomerization chemistry.

Entry 3. 1:0.5 Grubbs 1-BCF GC/MS trace with $CDCl_3$ as solvent.

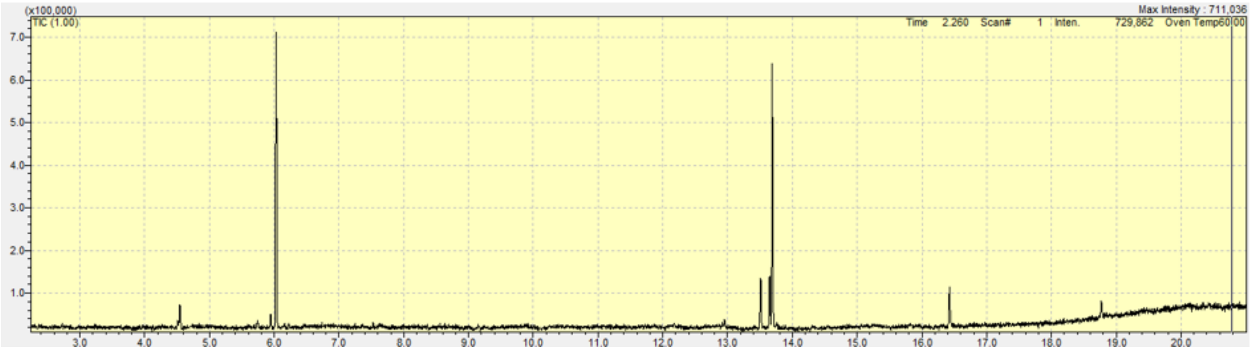

Figure S15. Entry 3. 240 minute aliquot of 1:0.5 GI-BCF in $CDCl_3$.

Entry 4. 1:1 Grubbs-BCF conversion and isomerization GC/MS trace with toluene as an internal standard.

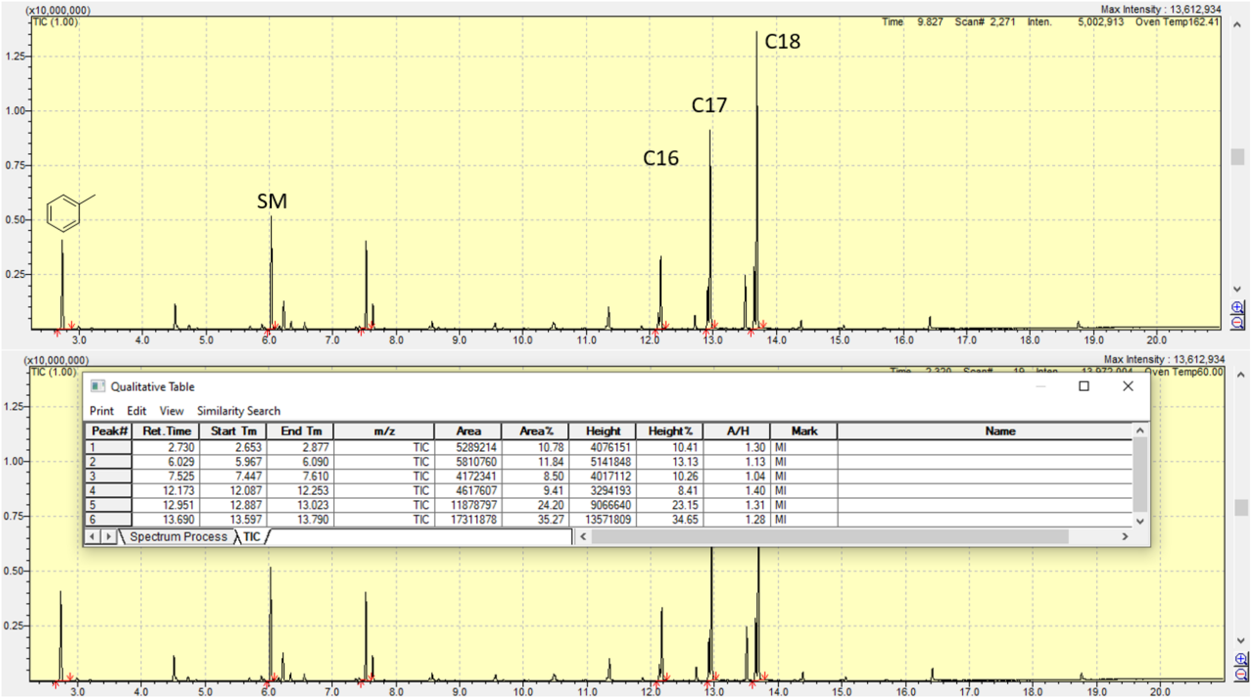

Peak#	Ret.Time	Start Tm	End Tm	m/z	Area	Area%	Height	Height%	A/H	Mark	Name
1	2.730	2.653	2.877	TIC	5289214	10.78	4076151	10.41	1.30	MI	
2	6.029	5.967	6.090	TIC	5810760	11.84	5141848	13.13	1.13	MI	
3	7.525	7.447	7.610	TIC	4172341	8.50	4017112	10.26	1.04	MI	
4	12.173	12.087	12.253	TIC	4617607	9.41	3294193	8.41	1.40	MI	
5	12.951	12.887	13.023	TIC	11878797	24.20	9066640	23.15	1.31	MI	
6	13.690	13.597	13.790	TIC	17311878	35.27	13571809	34.65	1.28	MI	

Figure S16. Entry 4. 240 minute aliquot of 1:1 Grubbs 1/BCF reaction with toluene as an internal standard.

Entry 5. 1:1:1 Grubbs 1/BCF/PCy_3 GC/MS trace.

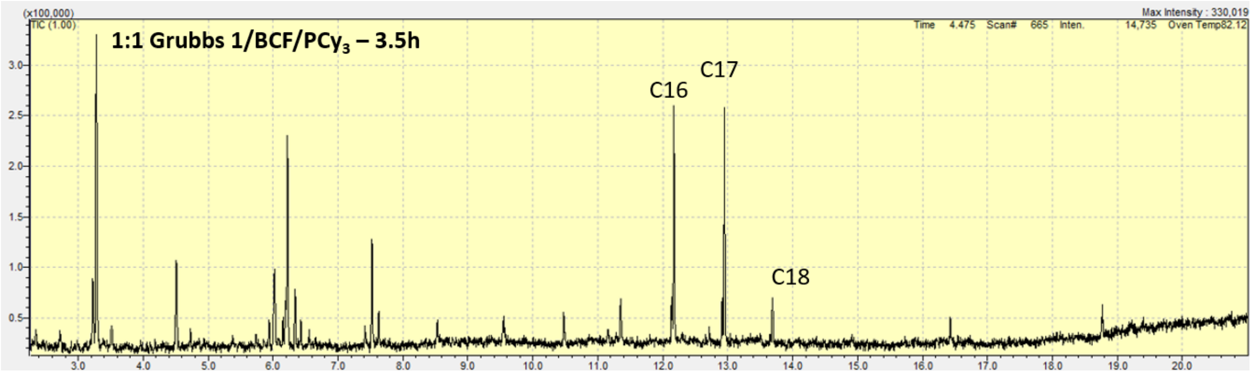

Figure S17. Entry 5. 210-minute aliquot of 1:1:1 Grubbs 1/BCF/PCy3 additive reaction. Predominant isomerization products observed and not further quantified.

Entry 6. BCF only GC/MS trace.

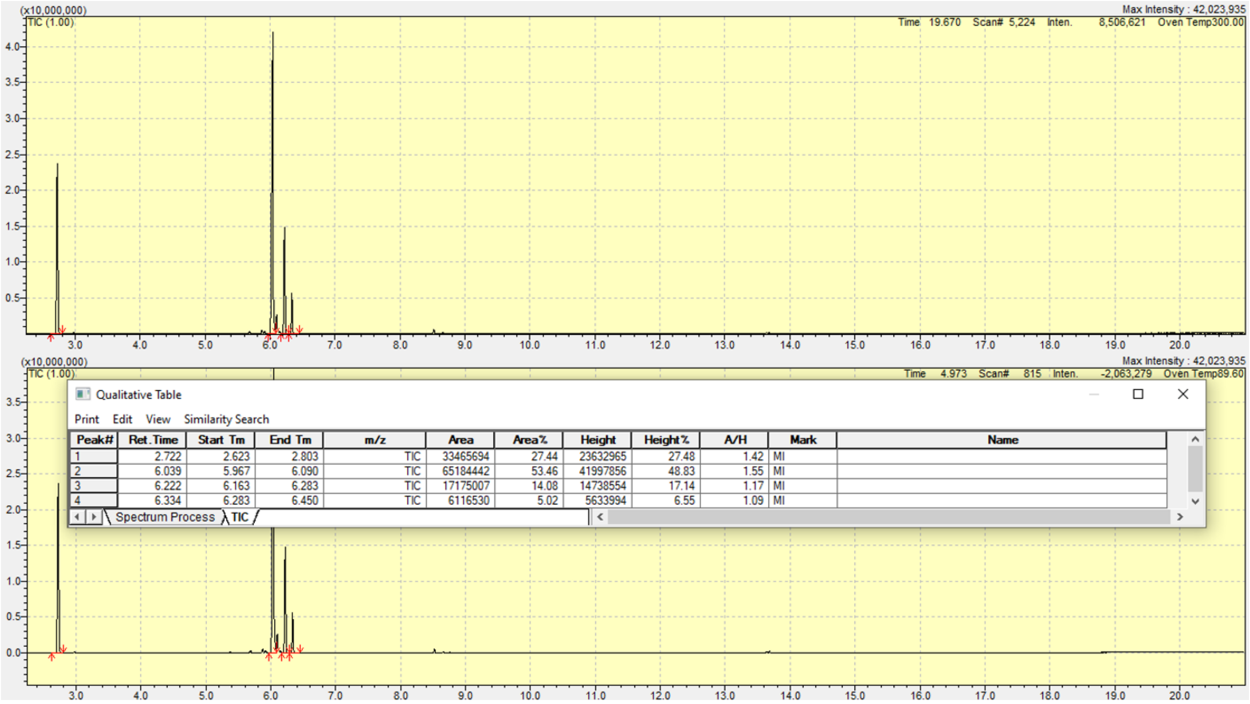

Peak#	Ret.Time	Start Tm	End Tm	m/z	Area	Area%	Height	Height%	A/H	Mark	Name
1	2.722	2.623	2.803	TIC	33465694	27.44	23632965	27.48	1.42	MI	
2	6.039	5.967	6.090	TIC	65184442	53.46	41997856	48.83	1.55	MI	
3	6.222	6.163	6.283	TIC	17175007	14.08	14738554	17.14	1.17	MI	
4	6.334	6.283	6.450	TIC	6116530	5.02	5633994	6.55	1.09	MI	

Figure S18. Entry 6. 240-minute aliquot of BCF + Decene reaction with toluene as an internal standard. No metathesis product observed.

Entry 7. 1:0.5 Grubbs 1/NaBArF GC/MS trace.

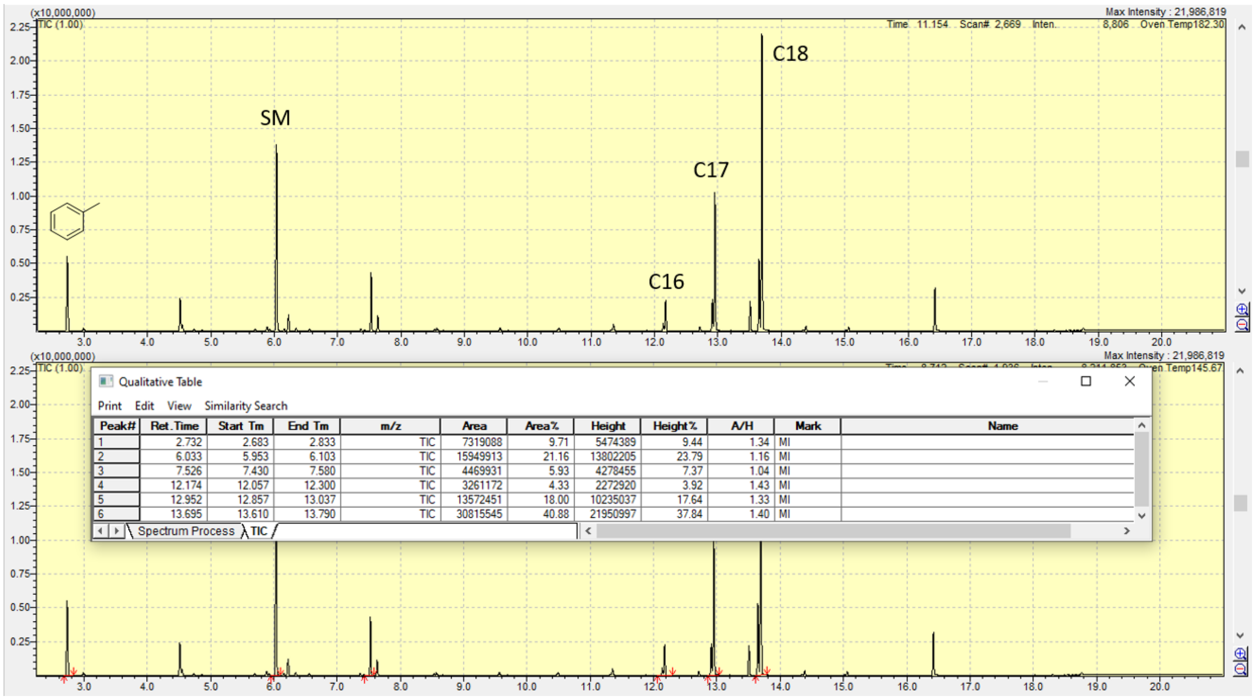

Peak#	Ret.Time	Start Tm	End Tm	m/z	Area	Area%	Height	Height%	A/H	Mark	Name
1	2.732	2.683	2.833	TIC	7319088	9.71	5474389	9.44	1.34	MI	
2	6.033	5.953	6.103	TIC	15949913	21.16	13802205	23.79	1.16	MI	
3	7.526	7.430	7.580	TIC	4469931	5.93	4278455	7.37	1.04	MI	
4	12.174	12.057	12.300	TIC	3261172	4.33	2272920	3.92	1.43	MI	
5	12.952	12.857	13.037	TIC	13572451	18.00	10235037	17.64	1.33	MI	
6	13.695	13.610	13.790	TIC	30815545	40.88	21950997	37.84	1.40	MI	

Figure S19. Entry 7. NaBArF activated Grubbs 1. 4h reaction time with toluene as an internal standard.

Entry 8. 1:0.5 Grubbs 1/AgOTs GC/MS trace.

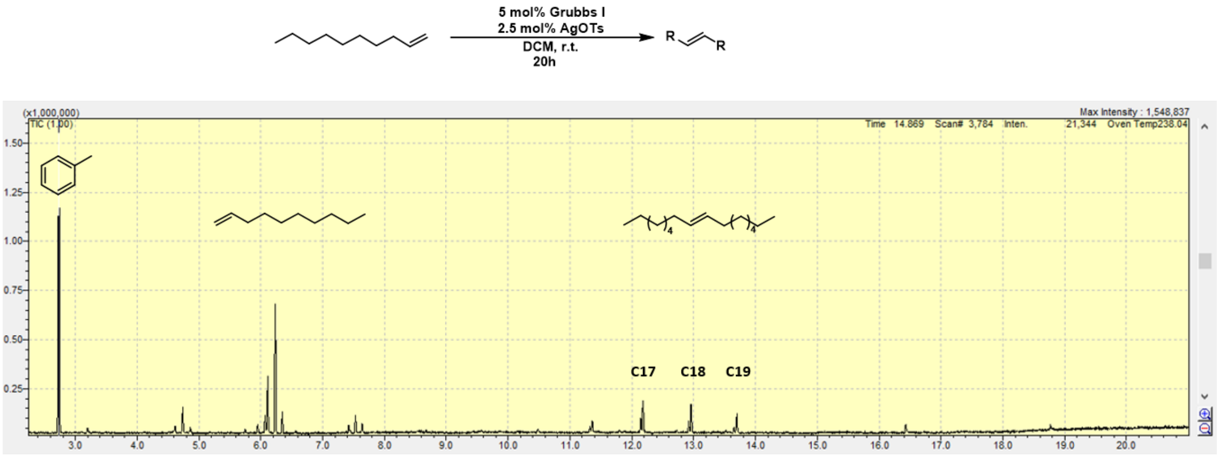

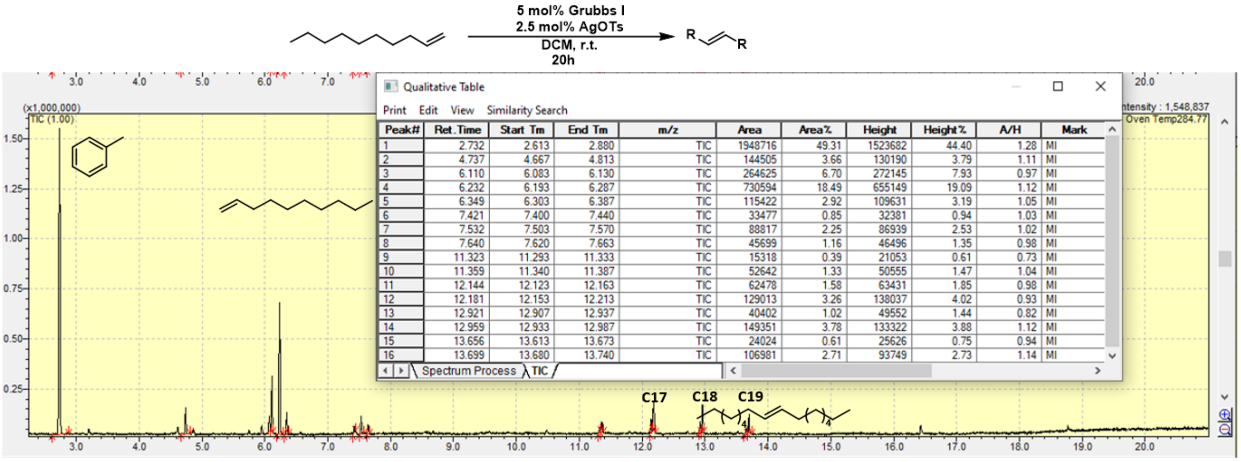

Peak#	Ret.Time	Start Tm	End Tm	m/z	Area	Area%	Height	Height%	A/H	Mark
1	2.732	2.613	2.880	TIC	1948716	49.31	1523682	44.40	1.28	MI
2	4.737	4.667	4.813	TIC	144505	3.66	130190	3.79	1.11	MI
3	6.110	6.083	6.130	TIC	264625	6.70	272145	7.93	0.97	MI
4	6.232	6.193	6.287	TIC	730594	18.49	655149	19.09	1.12	MI
5	6.349	6.303	6.387	TIC	115422	2.92	109631	3.19	1.05	MI
6	7.421	7.400	7.440	TIC	33477	0.85	32381	0.94	1.03	MI
7	7.532	7.503	7.570	TIC	88817	2.25	86939	2.53	1.02	MI
8	7.640	7.620	7.663	TIC	45699	1.16	46496	1.35	0.98	MI
9	11.323	11.293	11.333	TIC	15318	0.39	21053	0.61	0.73	MI
10	11.359	11.340	11.387	TIC	52642	1.33	50555	1.47	1.04	MI
11	12.144	12.123	12.163	TIC	62478	1.58	63431	1.85	0.98	MI
12	12.181	12.153	12.213	TIC	129013	3.26	138037	4.02	0.93	MI
13	12.921	12.907	12.937	TIC	40402	1.02	49552	1.44	0.82	MI
14	12.959	12.933	12.987	TIC	149351	3.78	133322	3.88	1.12	MI
15	13.656	13.613	13.673	TIC	24024	0.61	25626	0.75	0.94	MI
16	13.699	13.680	13.740	TIC	106981	2.71	93749	2.73	1.14	MI

Figure S20. Entry 8. AgOTS + Grubbs 1 reaction with toluene as an internal standard GC/MS aliquot.

Entry 9. 1:2 Grubbs 1/BCF GC/MS trace.

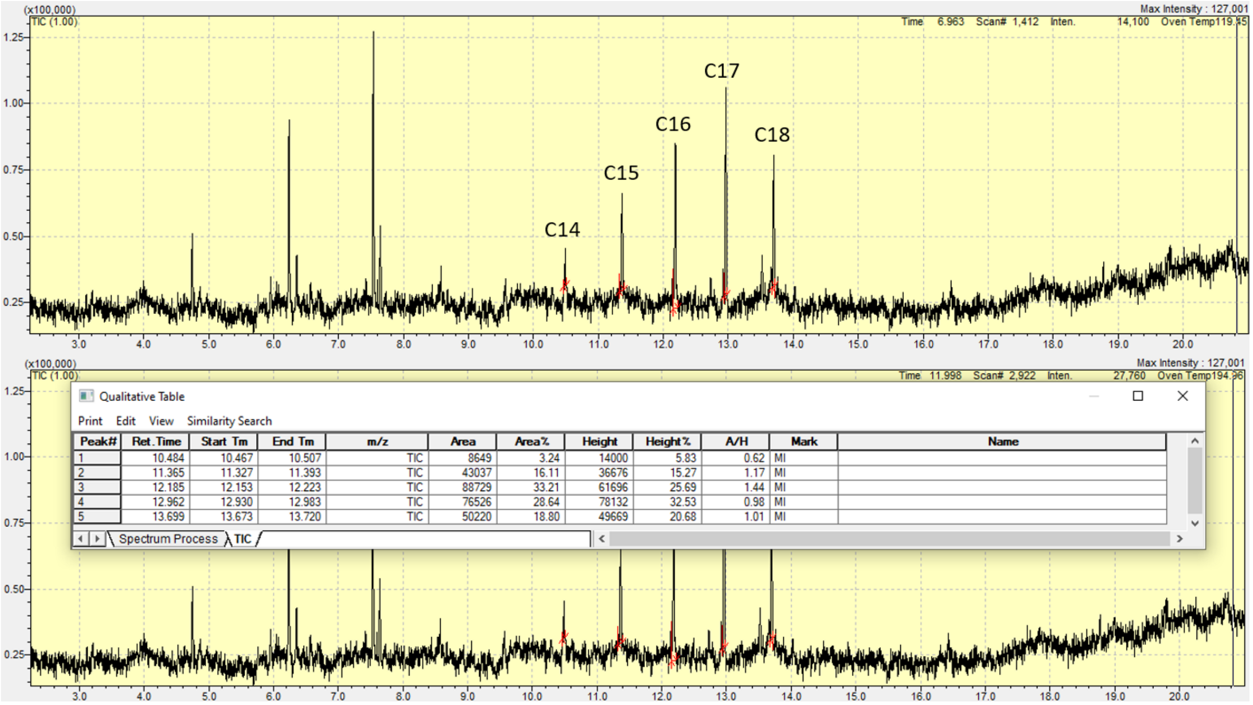

Peak#	Ret.Time	Start Tm	End Tm	m/z	Area	Area%	Height	Height%	A/H	Mark	Name
1	10.484	10.467	10.507	TIC	8649	3.24	14000	5.83	0.62	MI	
2	11.365	11.327	11.393	TIC	43037	16.11	36676	15.27	1.17	MI	
3	12.185	12.153	12.223	TIC	88729	33.21	61696	25.69	1.44	MI	
4	12.962	12.930	12.983	TIC	76526	28.64	78132	32.53	0.98	MI	
5	13.699	13.673	13.720	TIC	50220	18.80	49669	20.68	1.01	MI	

Figure S21. Entry 9. 240-minute aliquot of 1:2 Grubbs 1/BCF reaction. 10 mol % BCF with 5 mol % Grubbs 1. % Isomerization of metathesis products determined relative to C18 peak. Other isomerization products of starting material not included in this determination.

Entry 10. 1:0.5 Grubbs 1/$B(OH)_3$ GC/MS trace.

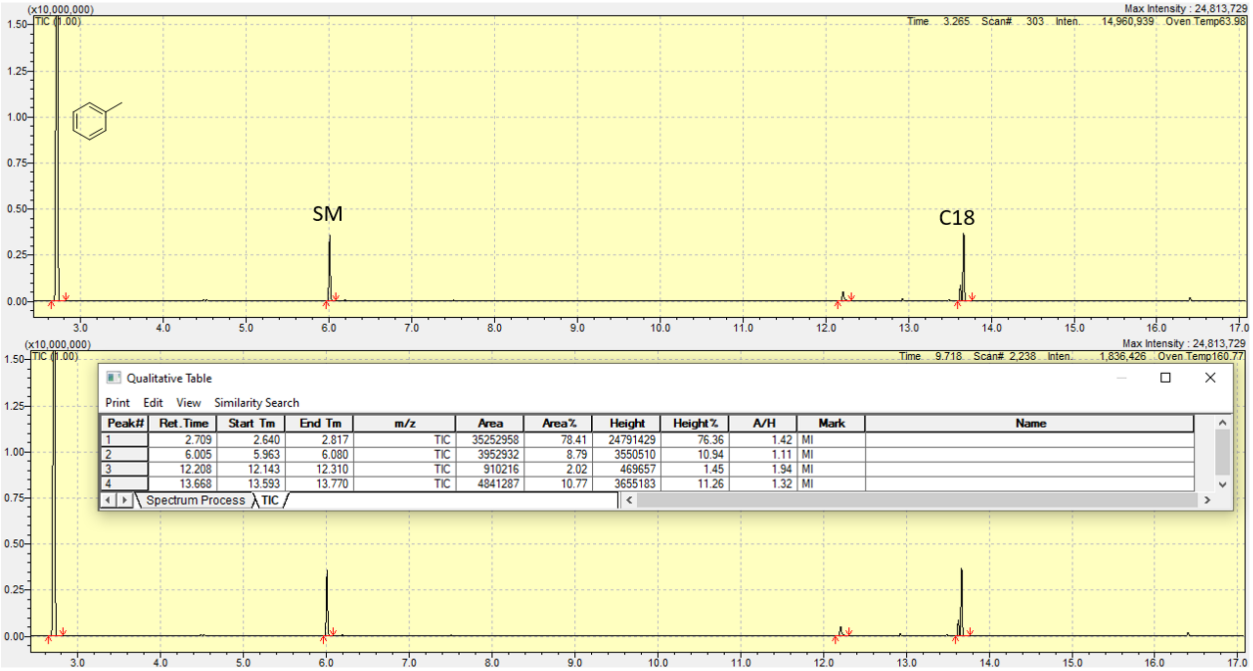

Peak#	Ret.Time	Start Tm	End Tm	m/z	Area	Area%	Height	Height%	A/H	Mark	Name
1	2.709	2.640	2.817	TIC	35252958	78.41	24791429	76.36	1.42	MI	
2	6.005	5.963	6.080	TIC	3952932	8.79	3550510	10.94	1.11	MI	
3	12.208	12.143	12.310	TIC	910216	2.02	469657	1.45	1.94	MI	
4	13.668	13.593	13.770	TIC	4841287	10.77	3655183	11.26	1.32	MI	

Figure S22. Entry 10. 240-minute aliquot of 2.5 mol % boric acid 5 mol % Grubbs 1 reaction. Shouldered peak at 12.2 minutes is a phthalate impurity on the GC/MS column.

Entry 11. 1:0.5 Grubbs-DPPF+BCF GC/MS trace.

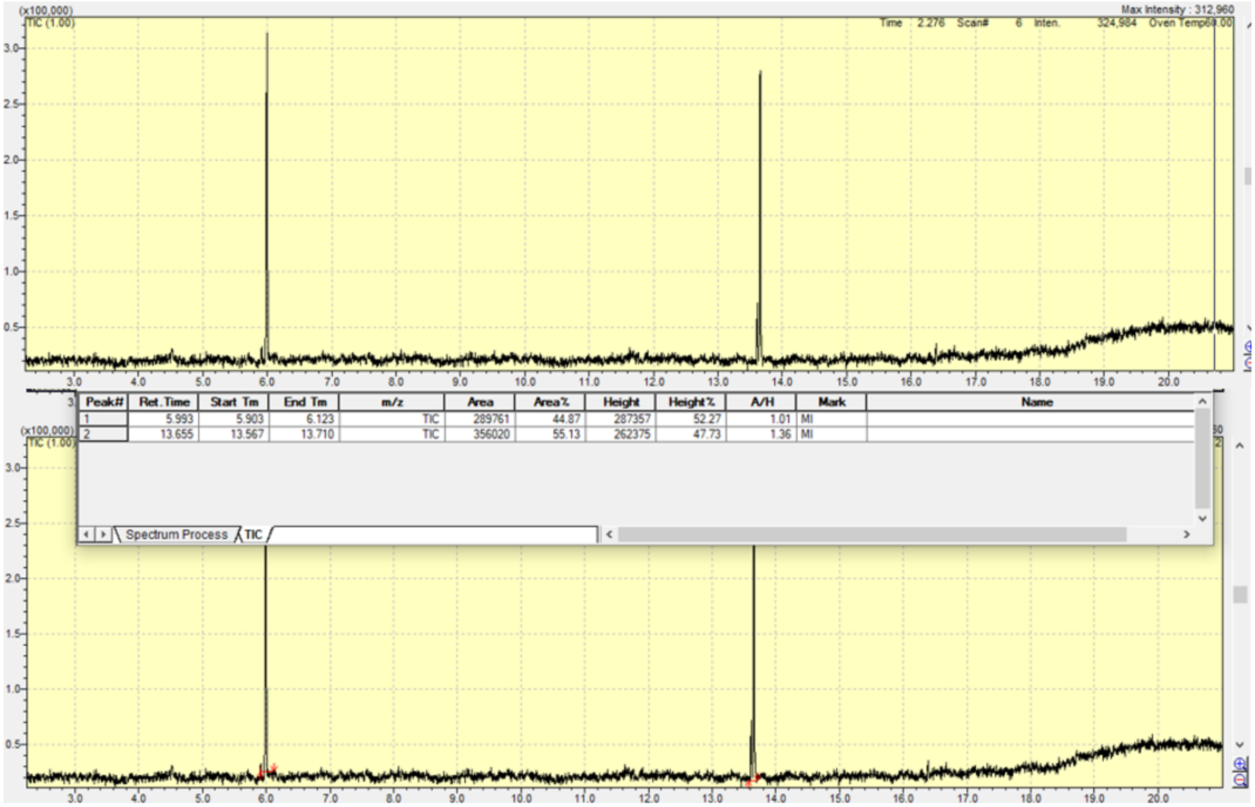

Figure S23. Entry 11. 240-minute aliquot of 2.5 mol % BCF 5 mol % Grubbs-DPPF reaction. 47% conversion of starting material is observed with no isomerization products.

Entry 12. Grubbs-DPPF GC/MS trace.

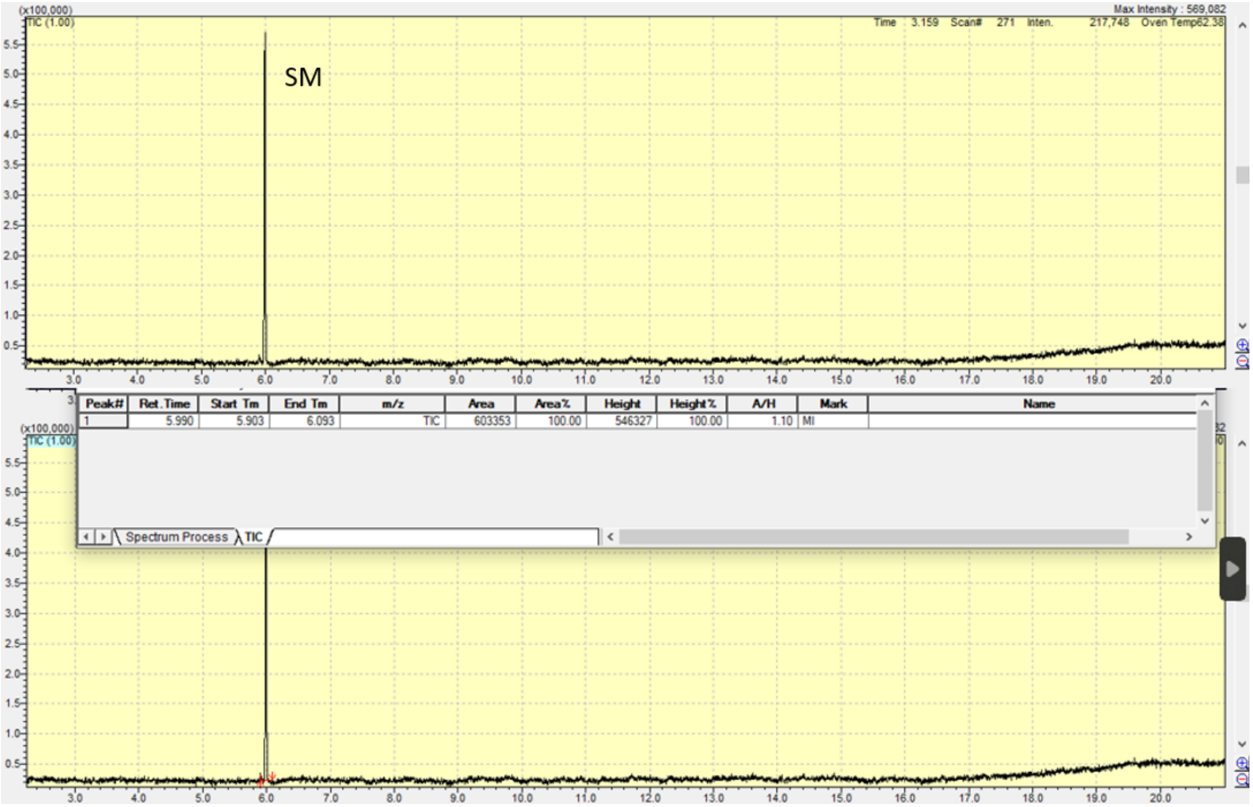

Figure S24. Entry 12. 240-minute aliquot of 5 mol % Grubbs-DPPF control reaction. No conversion of starting material is observed.

Entry 13. 5 mol% Grubbs-Xantphos GC/MS trace.

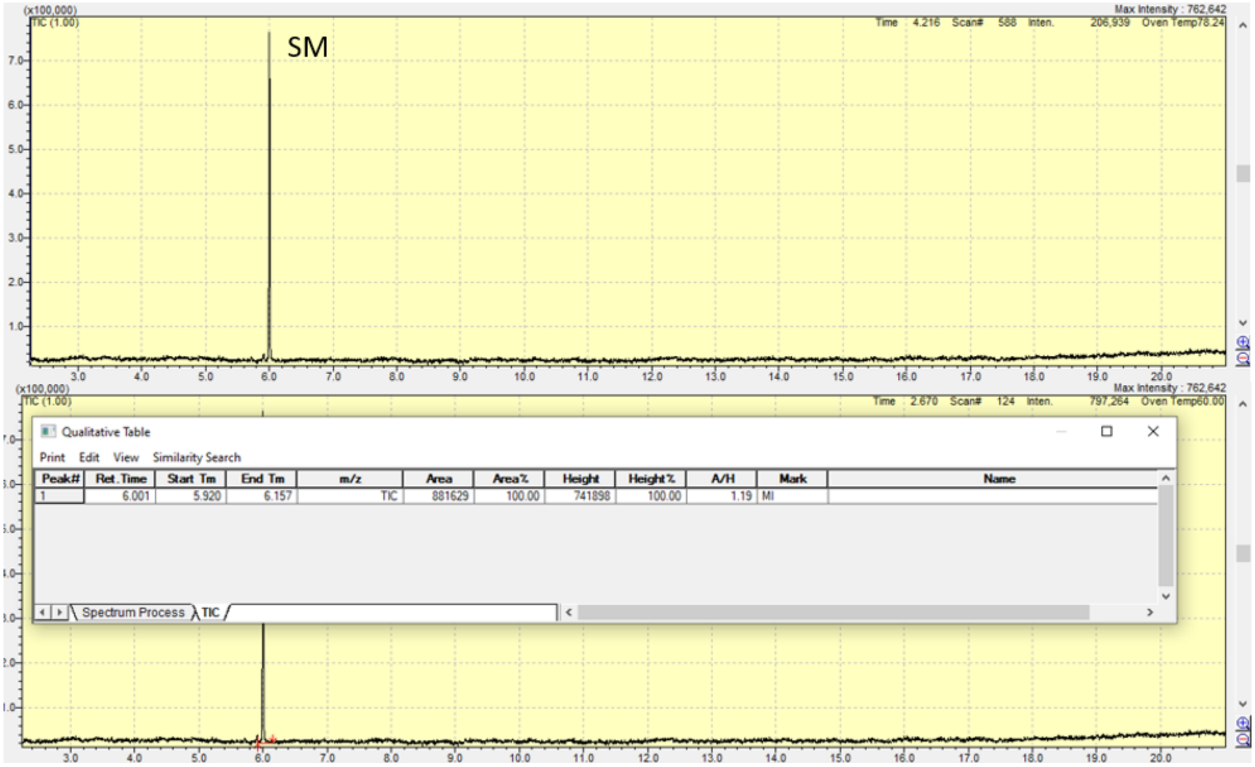

Figure S25. Entry 13. 20h aliquot of 5 mol % Grubbs-Xantphos control reaction. No conversion of starting material is observed.

Entry 14. 5 mol% Grubbs-Xantphos GC/MS trace.

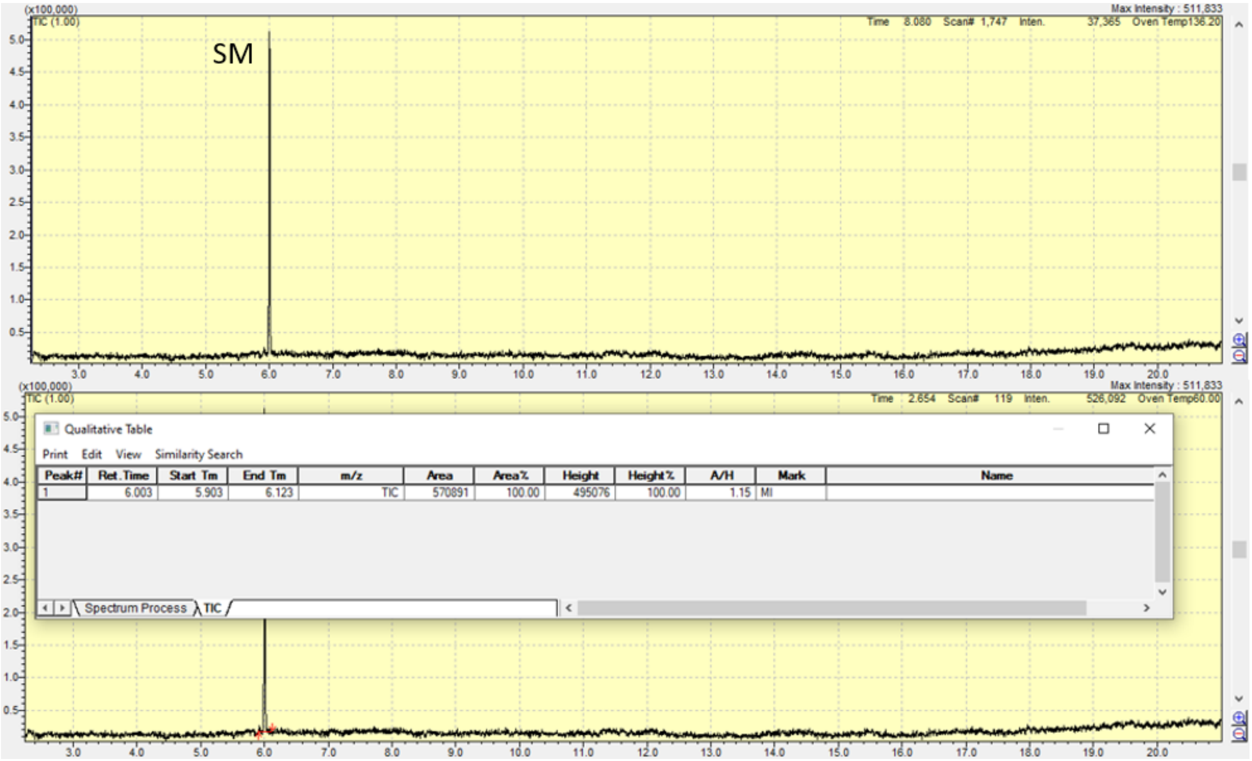

Figure S26. Entry 14. 20h aliquot of 5 mol % Grubbs-Xantphos 2.5 mol % BCF reaction. No conversion of starting material is observed.

$^{31}P\{^1H\}$ NMR spectrum of 0.5:1 BCF – Grubbs 1.

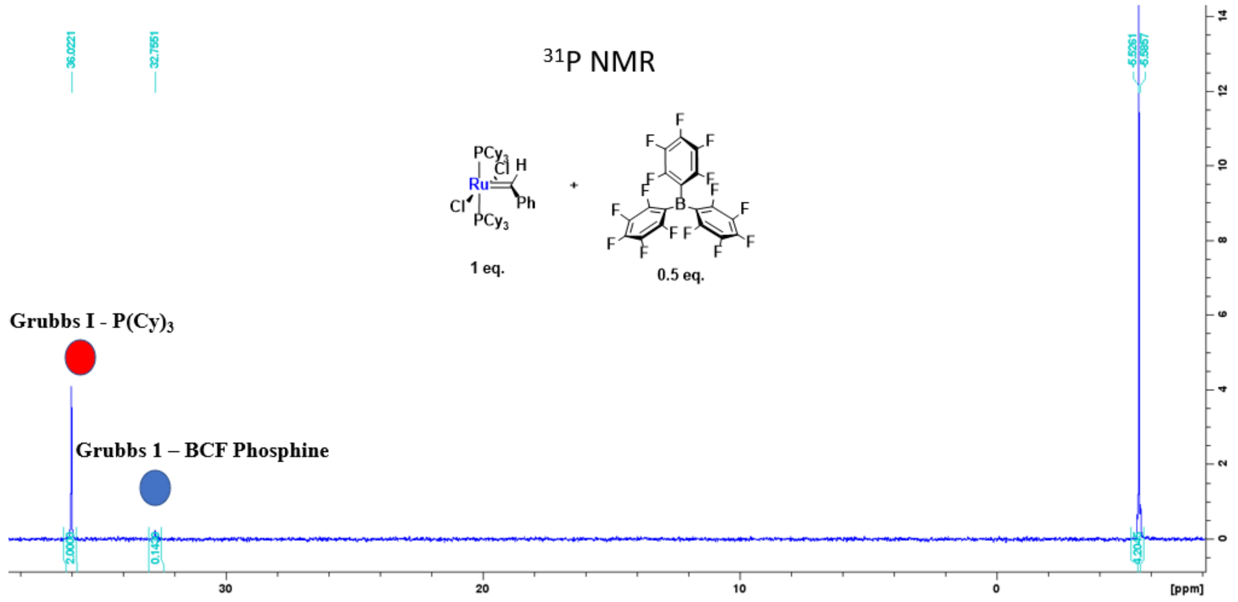

Figure S27. Authentic $^{31}P\{^1H\}$ NMR spectrum of Grubbs 1 and 0.5 eq of BCF with a sealed capillary of PPh_3 as an internal standard in CD_2Cl_2.

$^{31}P\{^1H\}$ NMR spectrum of 0.5:1 H-BCF- Grubbs 1.

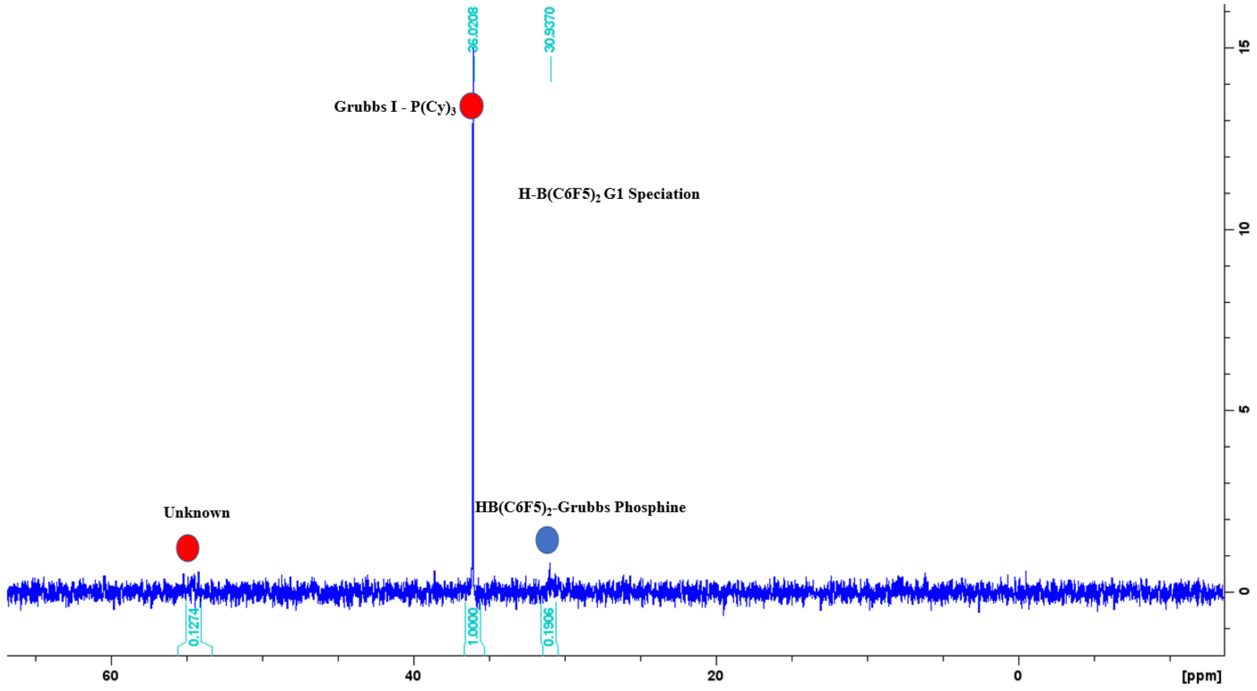

Figure S28. $^{31}P\{^1H\}$ NMR spectrum of 1:0.5 ratio of Grubbs 1/H-BCF speciation in CD_2Cl_2.

$^{31}P\{^1H\}$ NMR spectrum of 0.5:1 Cp-BCF- Grubbs 1.

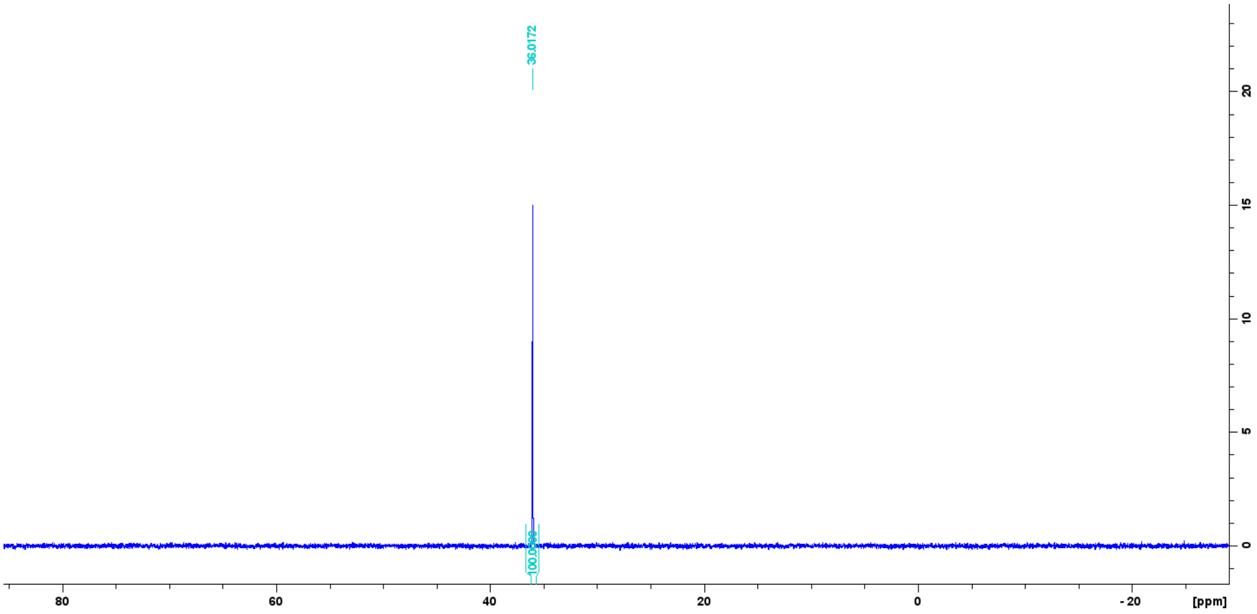

Figure S29. $^{31}P\{^1H\}$ NMR of 1:0.5 Grubbs 1/Cp-BCF adduct in CD_2Cl_2.

Cp-BCF - Grubbs 1 phosphine shift by $^{31}P\{^1H\}$ NMR remains unchanged from parent G1.

$^{31}P\{^1H\}$ NMR spectrum of NaBarF - Grubbs 1.

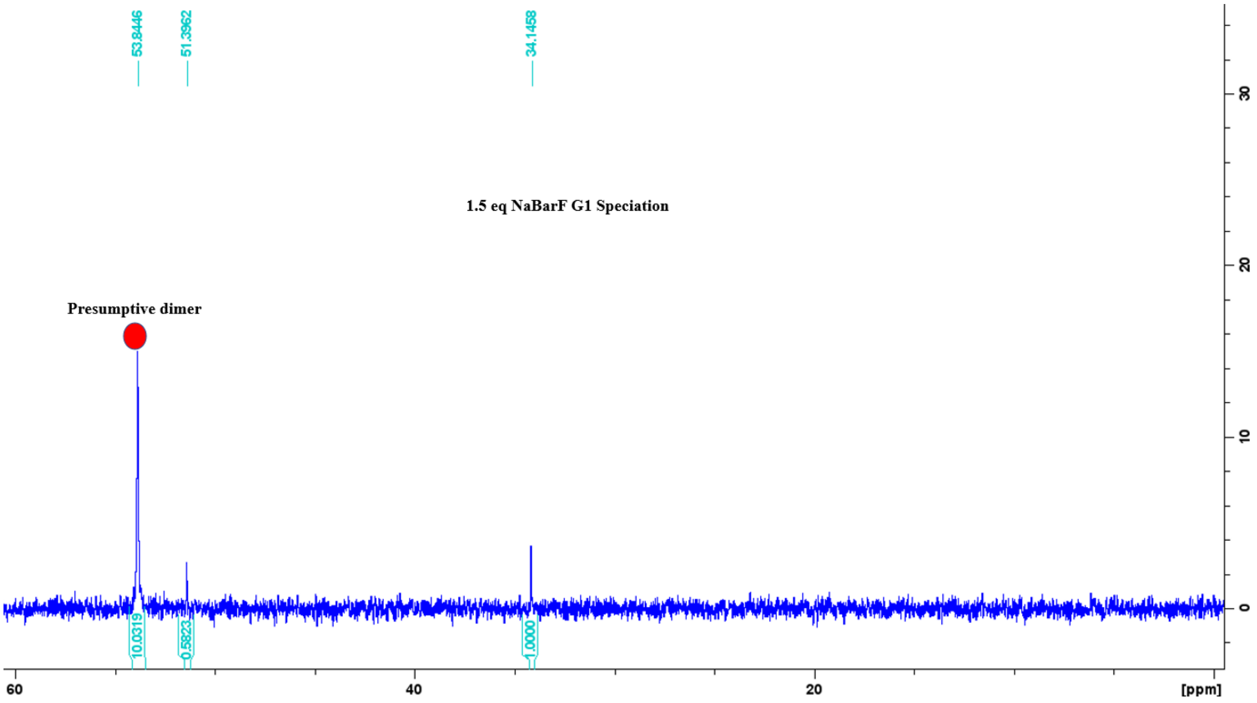

Figure S30. $^{31}P\{^1H\}$ NMR spectrum of 1.5 eq NaBArF to Grubbs 1. Entire phosphine peak converts mostly to 53 ppm phosphine.

$^{31}P\{^{1}H\}$ NMR spectrum of 1:0.5 GI-DPPF + BCF.

.

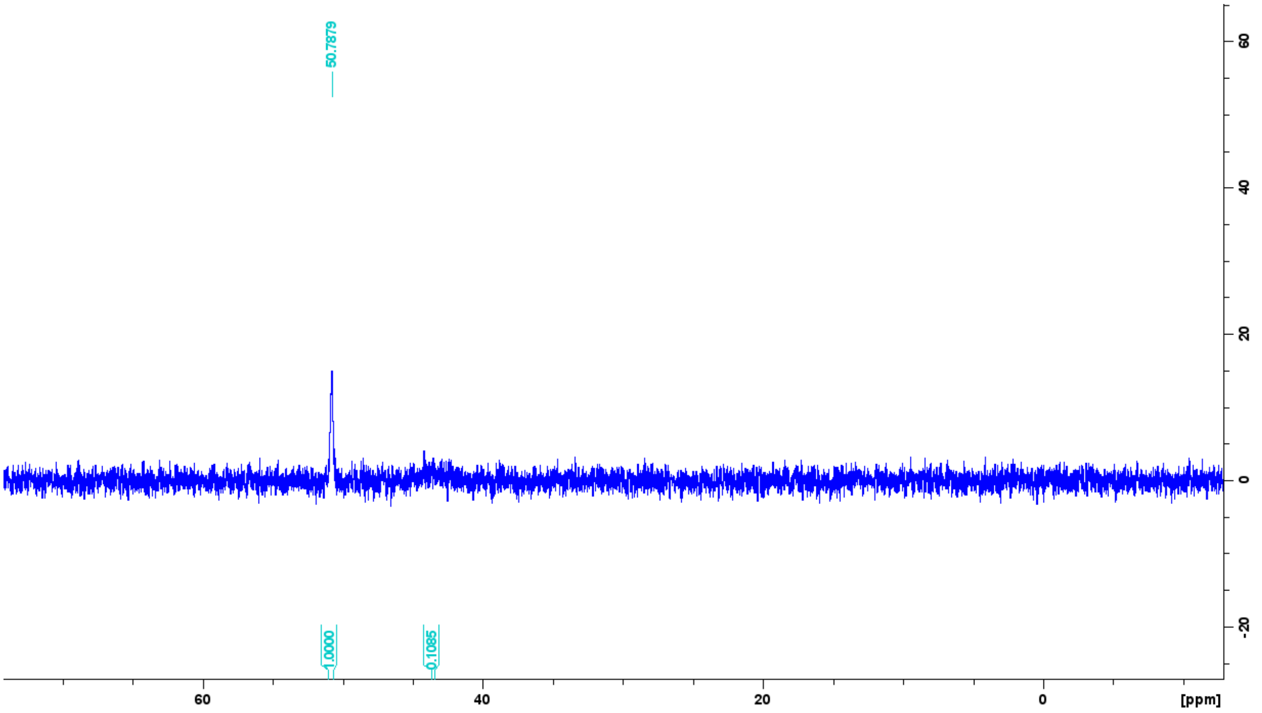

Figure S31. $^{31}P\{^{1}H\}$ NMR spectrum of 0.5 BCF to GI-DPPF in CD_2Cl_2. Parent phosphine peak observed with a minor peak at 43 ppm in a similar ratio of 10:1 to the minor upfield species similarly observed in S17. These integrations are relative, rather than quantitative as observed in S17.

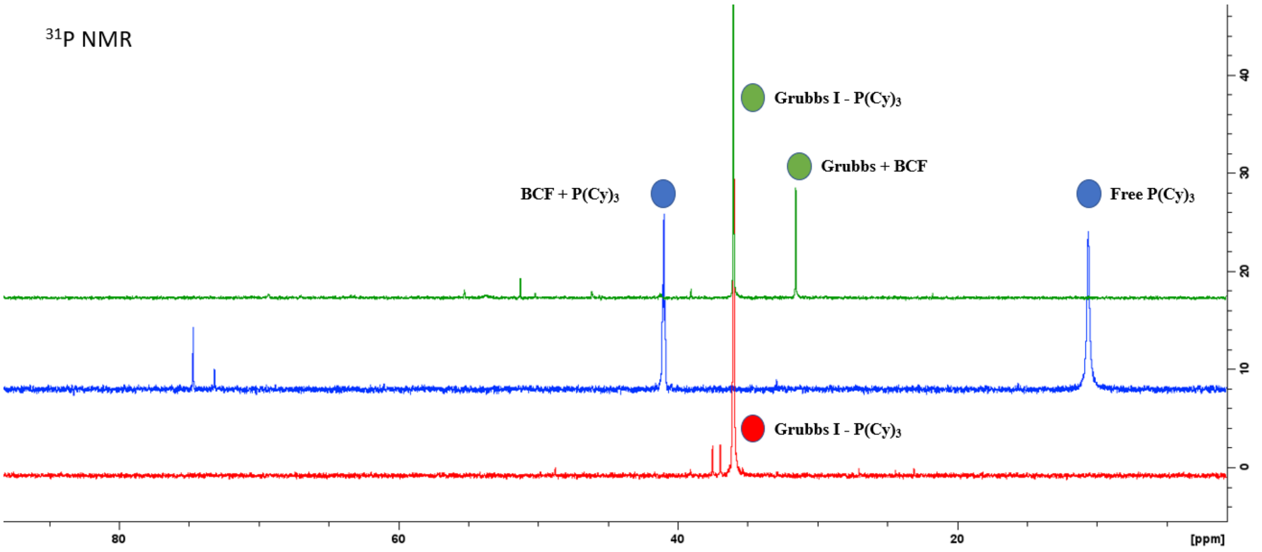

Figure S32. Stacked $^{31}P\{^{1}H\}$ NMR spectra of Grubbs 1, Grubbs 1 + BCF and an authentic BCF + PCy_3 solution in CD_2Cl_2.

The $^{31}P\{^{1}H\}$ speciation indicates the Grubbs 1 + BCF adduct looks neither like free BCF + $P(Cy)_3$ nor free $P(Cy)_3$. The Grubbs 1 + BCF signal does indicate more shielding of the phosphine center, likely due to the surrounding BCF bulk. However, this study demonstrates

there is no BCF-Phosphine adduct formed in the Grubbs 1 sample, as resonances do not match the authentic BCF-P(Cy)3 adduct synthesized in blue.

$^{19}F\{^1H\}$ NMR Speciation

$^{19}F\{^1H\}$ NMR spectrum of 1:0.5 Grubbs 1 + BCF.

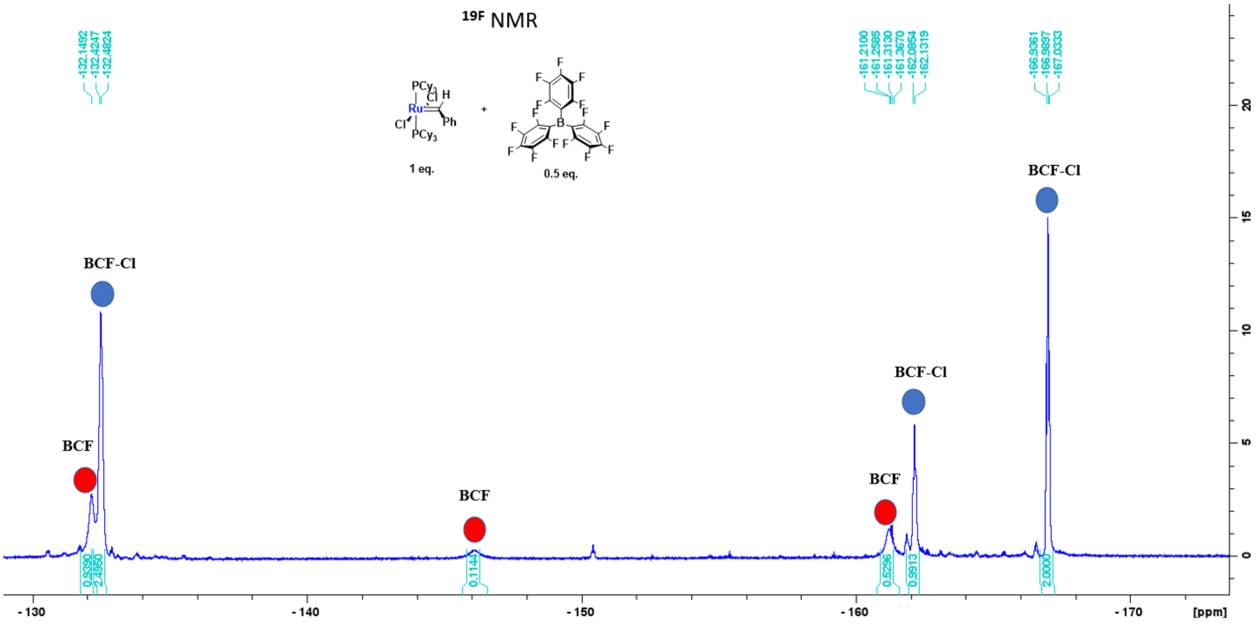

Figure S33.$^{19}F\{^1H\}$ NMR spectrum of 1:0.5 Grubbs 1/BCF speciation in CD_2Cl_2.

$^{19}F\{^1H\}$ NMR spectrum of 1:1 BCF - Grubbs 1.

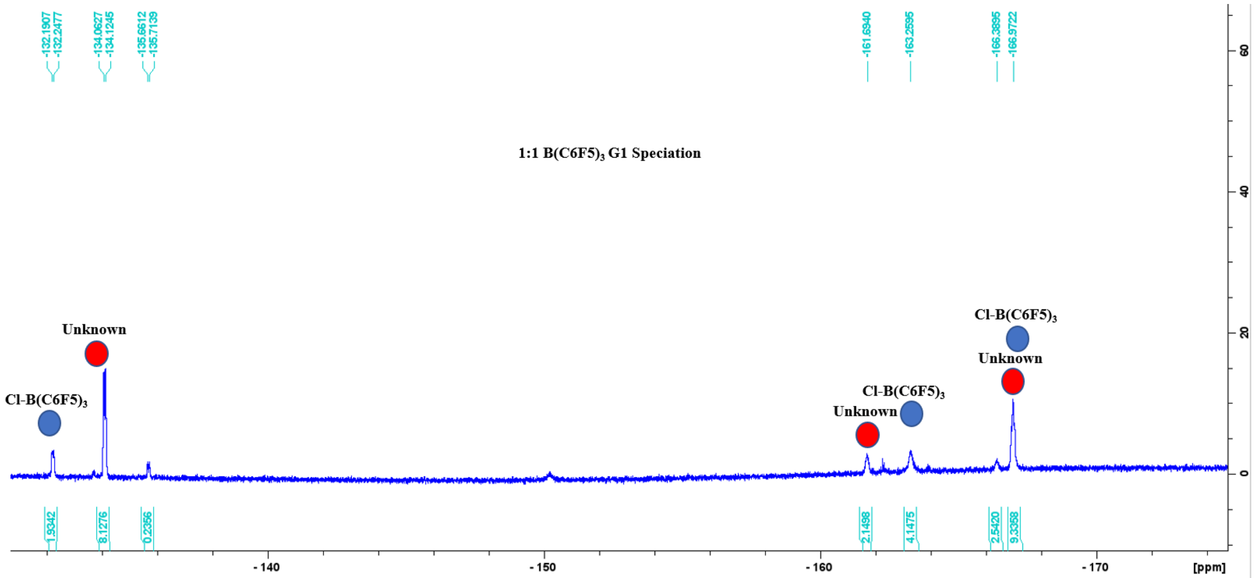

Figure S34. $^{19}F\{^1H\}$ NMR spectrum of 1:1 BCF-Grubbs in situ speciation in CD_2Cl_2.

1:1 Grubbs-BCF adduct almost completely converts to a different species, divergent from parent BCF and from BCF-Cl adducts. This result highlights the significance of the 1:0.5 ratio of Grubbs-BCF in maintaining the purported speciation to achieve controlled metathesis/ROMP.

$^{19}F\{^{1}H\}$ NMR spectrum of BCF-P(Cy)$_3$ adduct.

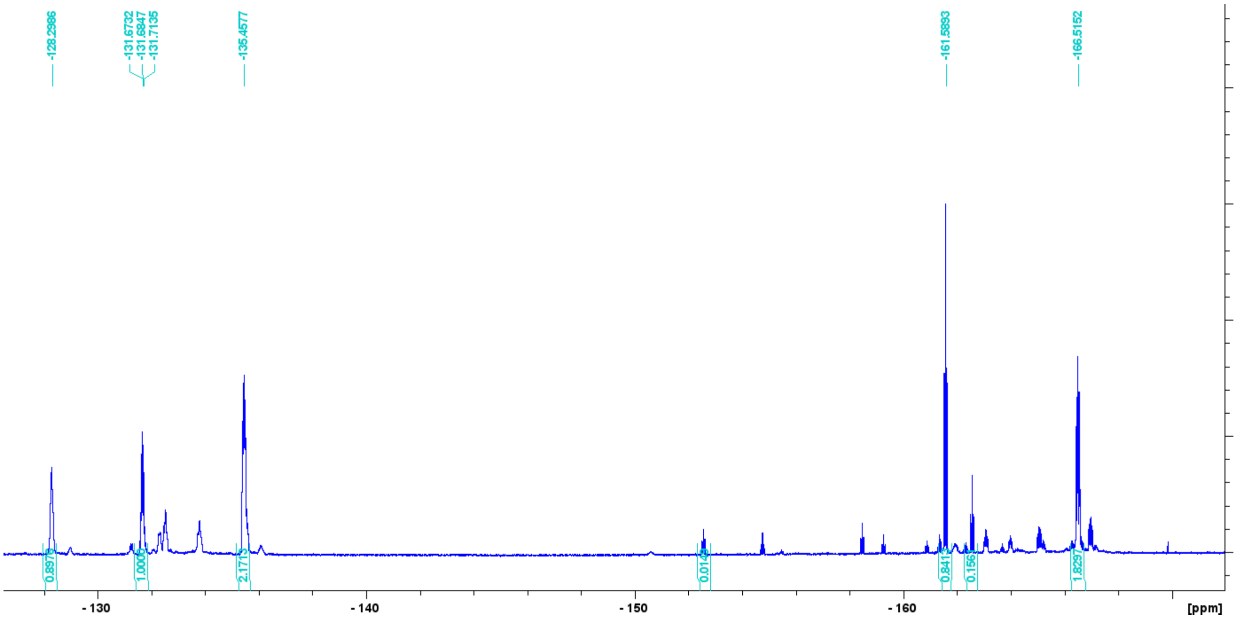

Figure S35. $^{19}F\{^{1}H\}$ NMR of an authentic solution of BCF-PCy$_3$ in CD_2Cl_2.

Stacked $^{19}F\{^{1}H\}$ NMR spectra of Grubbs 1 + BCF and authentic BCF-P(Cy)$_3$ adduct.

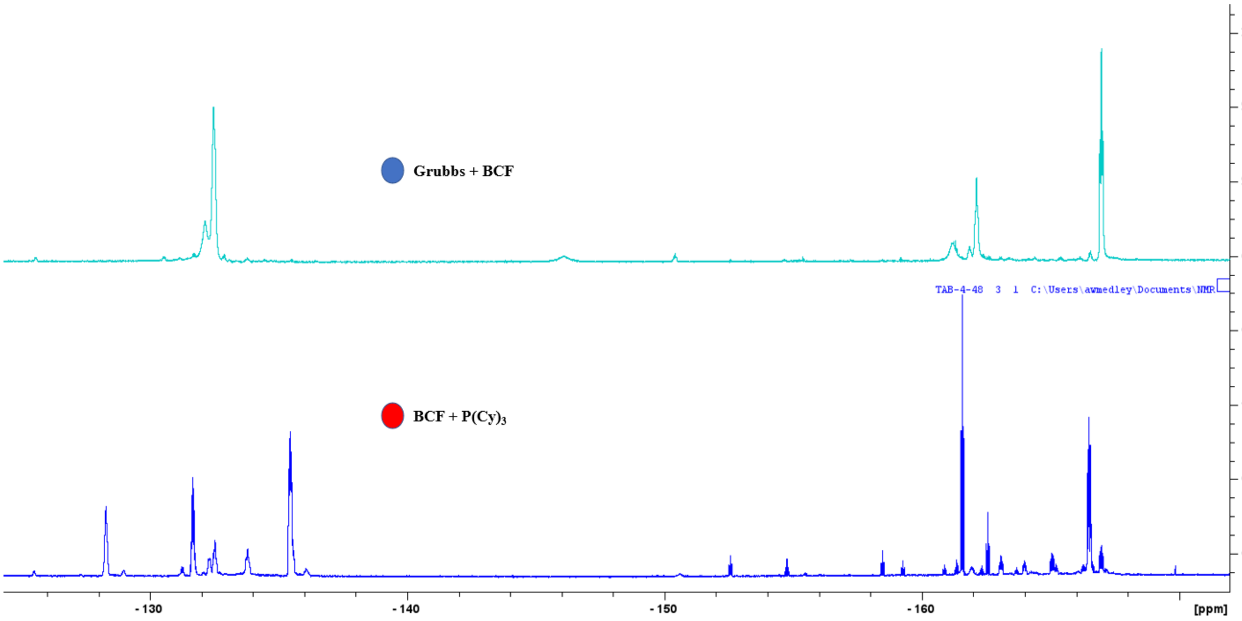

Figure S36. Stacked $^{19}F\{^{1}H\}$ NMR spectrum of Grubbs 1 + BCF and an authentic solution of BCF-PCy$_3$ in CD_2Cl_2.

The $^{19}F\{^{1}H\}$ NMR spectrum of Grubbs + BCF indicates the generation of a BCF-Cl adduct, which this abstraction has been demonstrated in similar organometallics species in the chemical literature. The authentic BCF-P(Cy)$_3$ adduct is complex, and dissimilar to that of the Grubbs 1-BCF by $^{19}F\{^{1}H\}$ NMR, indicating that the phosphine FLP adduct is not formed, but rather a [BCF][Cl] is. Moreover, the $^{19}F\{^{1}H\}$ NMR spectrum of the authentically made [BCF][Cl] anion matches spectroscopically the *in situ* proposed [BCF][Cl] abstracted from G1.

$^{19}F\{^{1}H\}$ NMR spectrum of 0.5:1 H-BCF - Grubbs 1.

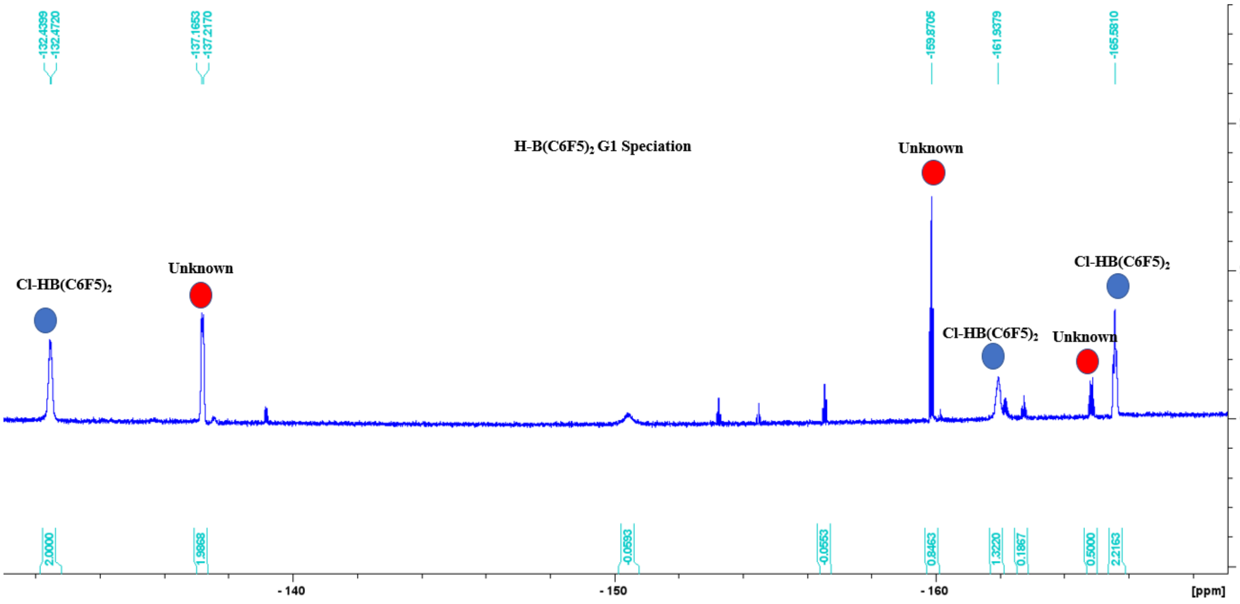

Figure S37. $^{19}F\{^{1}H\}$ NMR spectrum of 1:0.5 ratio of Grubbs 1/H-BCF speciation in in CD_2Cl_2.

$^{19}F\{^{1}H\}$ NMR spectrum of 1:1 H-BCF – PCy_3.

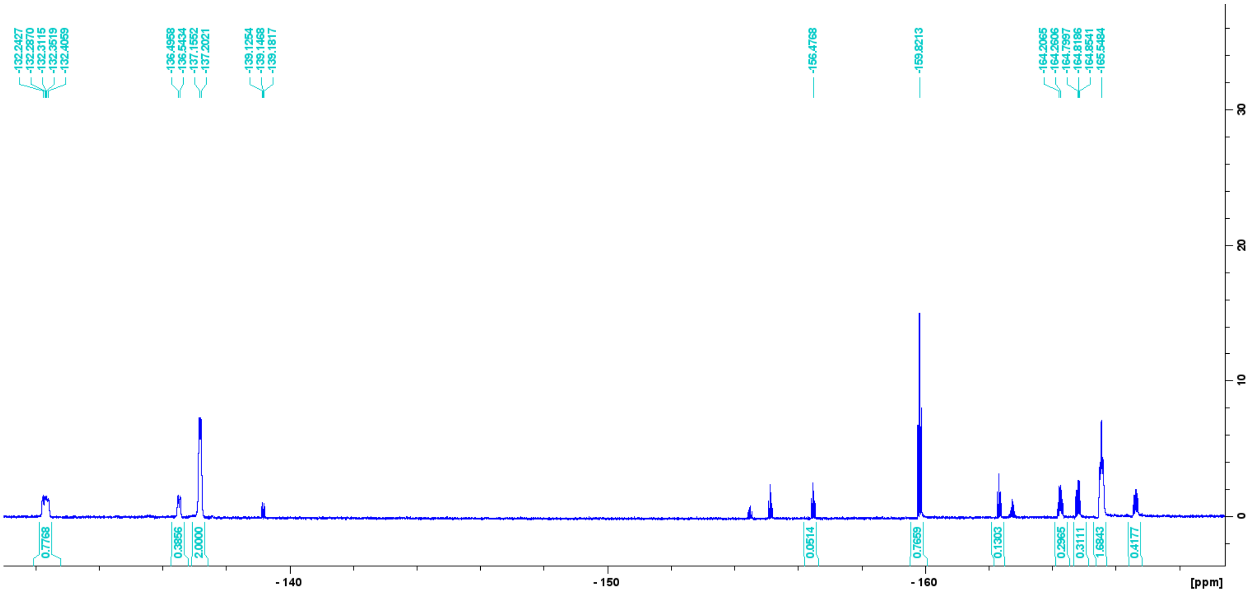

Figure S38. $^{19}F\{^{1}H\}$ NMR spectrum of 1:1 PCy_3/H-BCF speciation in in CD_2Cl_2.

Stacked $^{19}F\{^1H\}$ NMR spectra of Grubbs 1 + H-BCF and authentic H-BCF-P(Cy)$_3$ adduct.

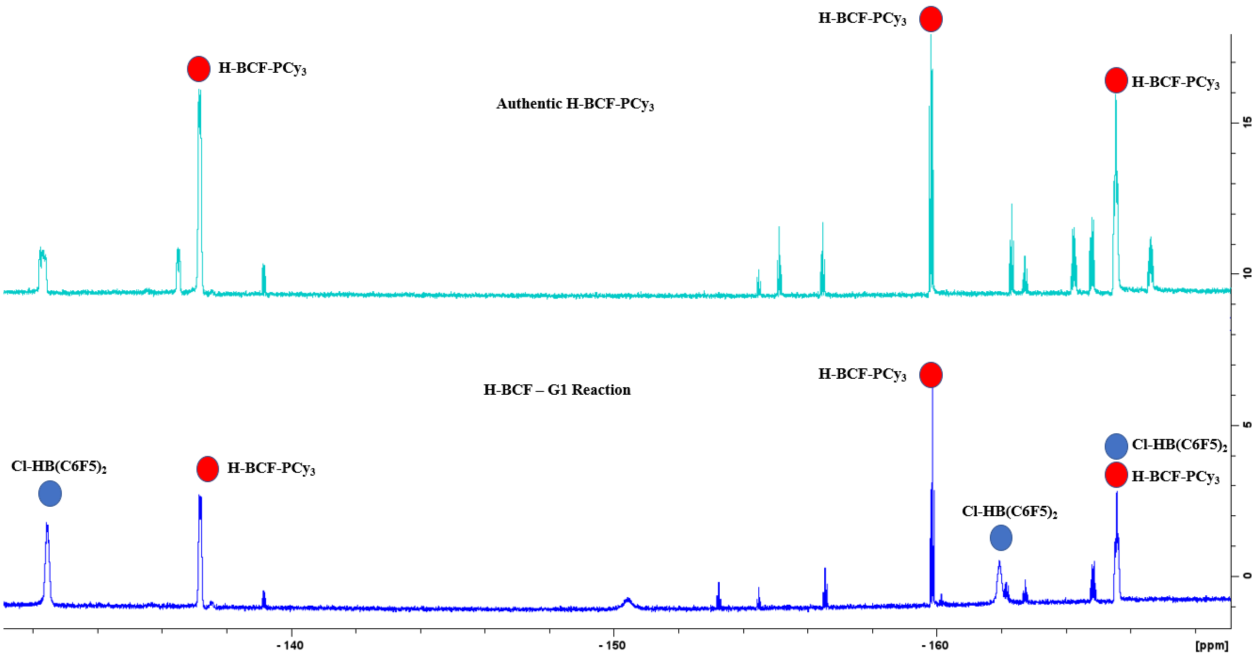

Figure S39. Stacked $^{19}F\{^1H\}$ NMR spectra of authentic H-BCF-PCy$_3$ and H-BCF-G1 in CD_2Cl_2.

When a 0.5:1 ratio of H-BCF:G1 was mixed and examined, the $^{19}F\{^1H\}$ NMR spectrum showed a mixture of products. Identifiable peaks were H-BCF chloride, as well as what appears to be the H-BCF-PCy$_3$ adduct. The broad peak within the reaction mixture appearing at -161 ppm is diagnostic of an anionic borate forming, such as in [H-BCF][Cl]. Overall, this result indicated that H-BCF was indeed the most reactive of the three boranes, as noted in the overall reactivity profiles, likely capable of abstracting chloride and phosphine concomitantly.

$^{19}F\{^1H\}$ NMR spectrum of 0.5:1 Cp-BCF- Grubbs 1.

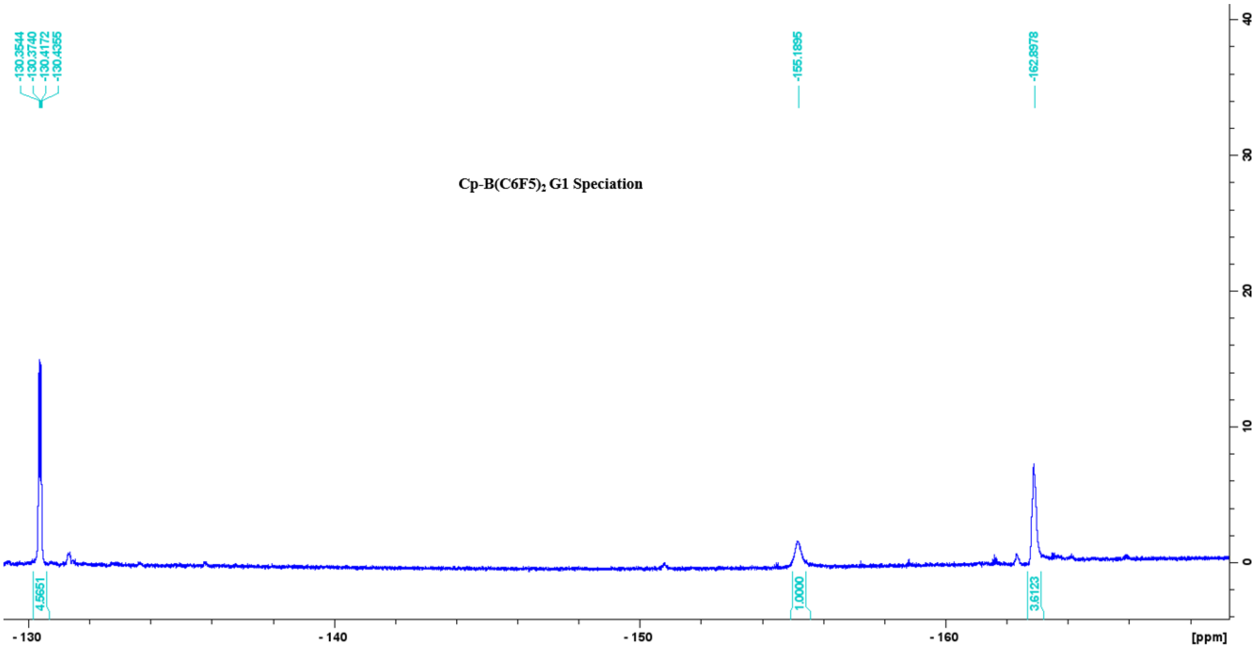

Figure S40. $^{19}F\{^{1}H\}$ NMR speciation of 1:0.5 Grubbs 1/BCF in CD_2Cl_2.

When 0.5/1 Cp-BCF/G1 was mixed and examined the $^{19}F\{^{1}H\}$ NMR spectrum revealed the parent Cp-BCF $^{19}F\{^{1}H\}$ NMR resonances. No further upfield shifted resonances were observed to indicate a [Cp-BCF][Cl] was formed, nor was any complex mixture of products observed, as was the case with H-BCF.

$[IrCp^{*}Cl_2]_2$ + BCF $^{19}F\{^{1}H\}$ NMR spectrum.

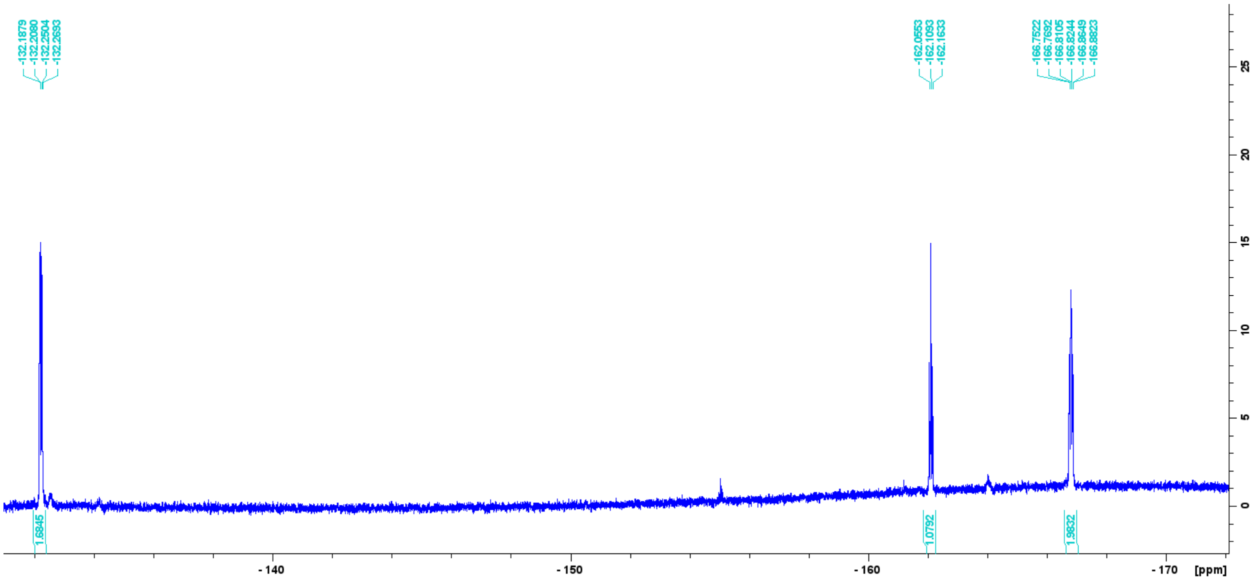

Figure S41. $^{19}F\{^{1}H\}$ NMR spectrum of authentically made BCF-Cl from the addition of $[IrCp^{*}Cl_2]_2$ to BCF in CD_2Cl_2.

GI-DPPF + 0.5 eq BCF $^{19}F\{^{1}H\}$ NMR spectrum.

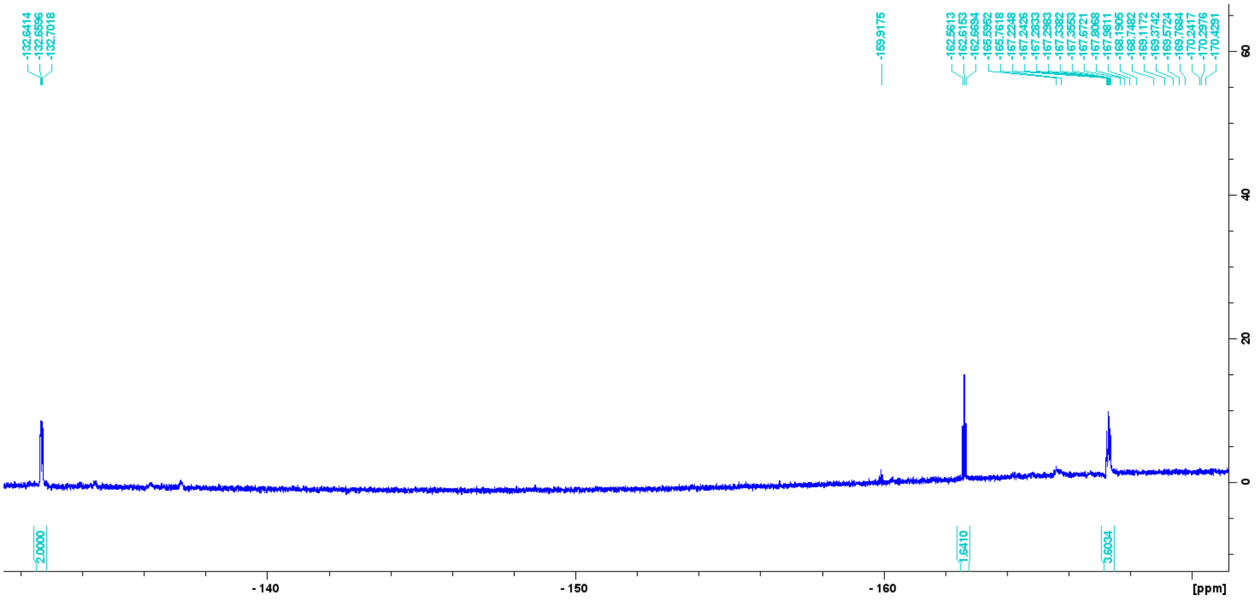

Figure S42. $^{19}F\{^{1}H\}$ NMR spectrum of GI-DPPF + 0.5 eq BCF. Shifts and integrations demonstrate full conversion to the [BCF][Cl] anion observed in the other speciation studies.

Analogous BCF-Cl ^{19}F NMR precedent.[3]

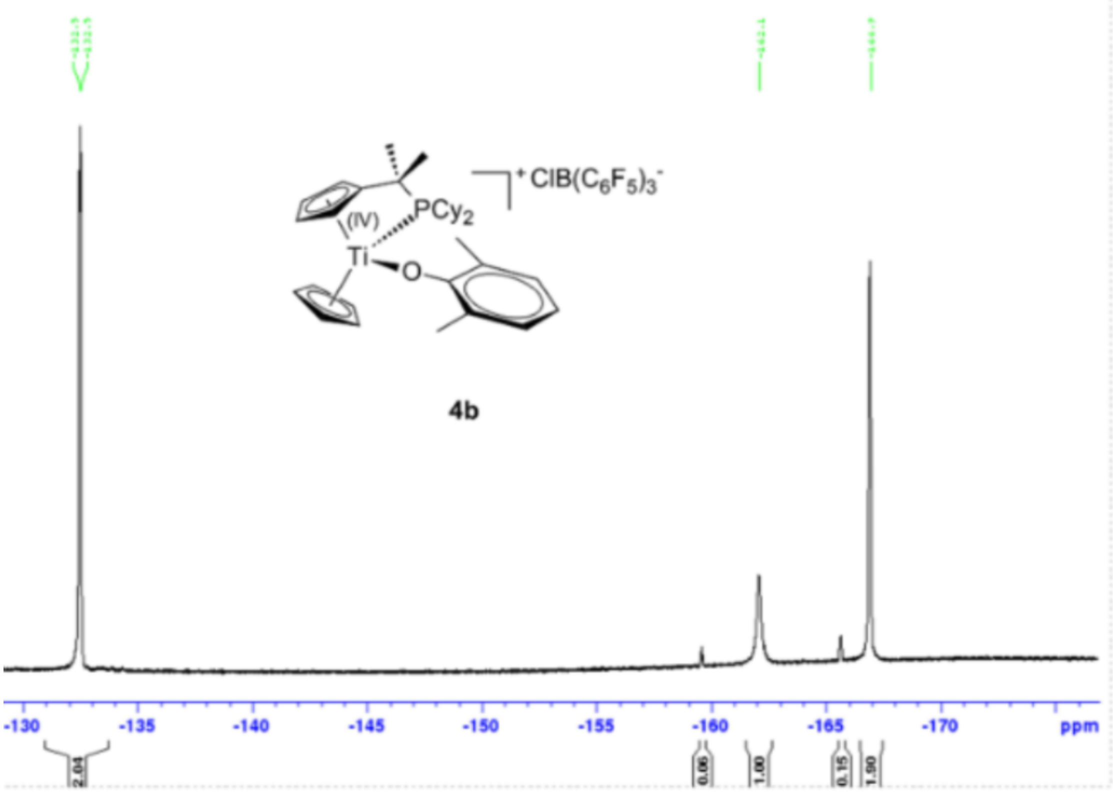

Figure S43. Analogous BCF-Cl $^{19}F\{^{1}H\}$ NMR spectrum reported in the chemical literature.[3]

In Situ $^{11}B\{^{1}H\}$ NMR spectrum of 1:0.5 Grubbs 1 - BCF.

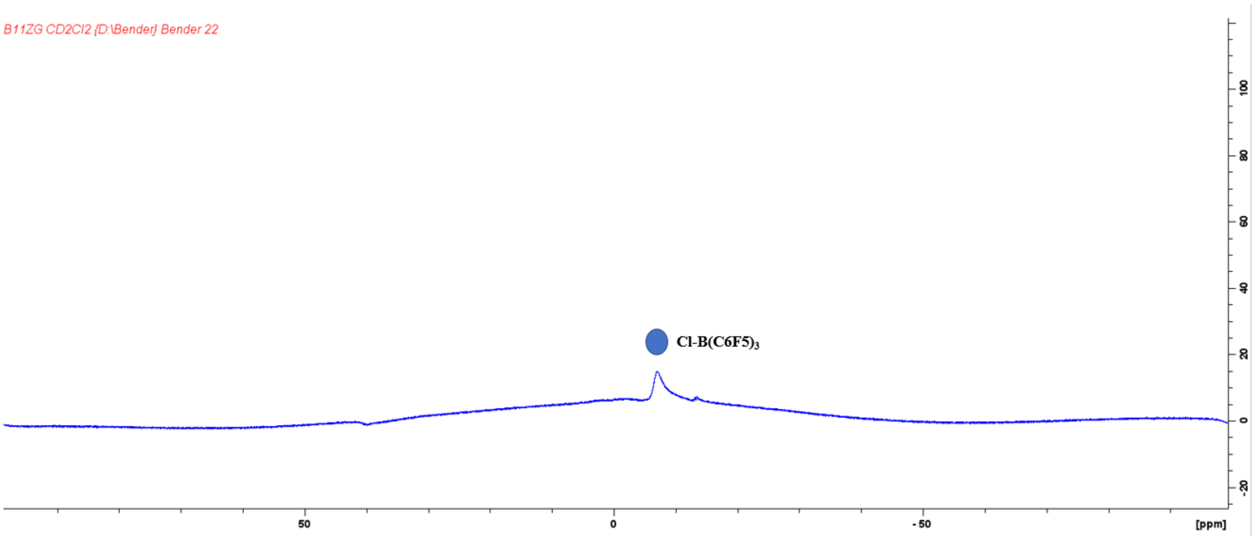

Figure S44. In Situ $^{11}B\{^{1}H\}$ NMR spectrum of Grubbs 1 + BCF in CD_2Cl_2.

The spectrum reveals a singlet by $^{11}B\{^{1}H\}$ NMR rather than a conventional P coupled doublet. Moreover, the diagnostic BCF peak at 59 ppm is consumed.

In Situ $^{11}B\{^{1}H\}$ NMR spectrum of the authentic 1:1 BCF + PCy_3 adduct.

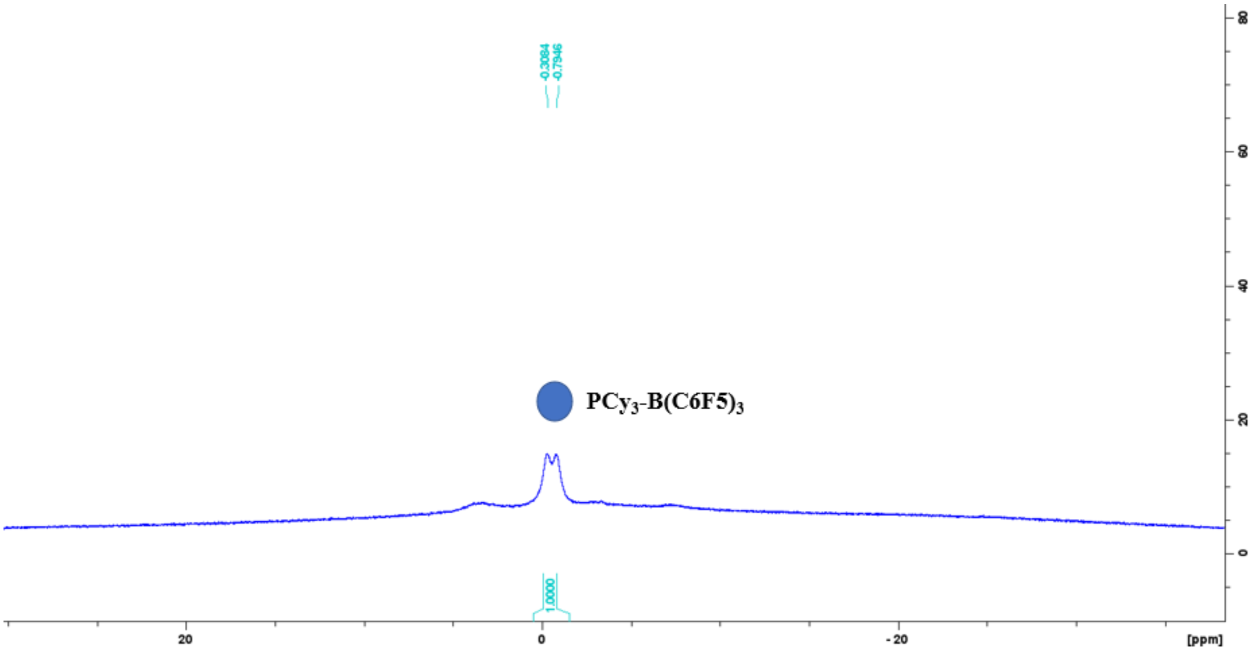

Figure S45. In Situ $^{11}B\{^{1}H\}$ NMR spectrum of the authentic BCF + PCy_3 in CD_2Cl_2.

The spectrum of the authentic PCy_3-BCF adduct shows a clear P coupled doublet by $^{11}B\{^{1}H\}$ NMR. This doublet is quite diagnostic in identifying FLP-type activity between borane and phosphines. The absence of this doublet in the BCF-G1 spectrum provides further insight into its speciation.

In Situ $^{11}B\{^{1}H\}$ NMR spectrum of the authentic 1:1 H-BCF + PCy_3 adduct.

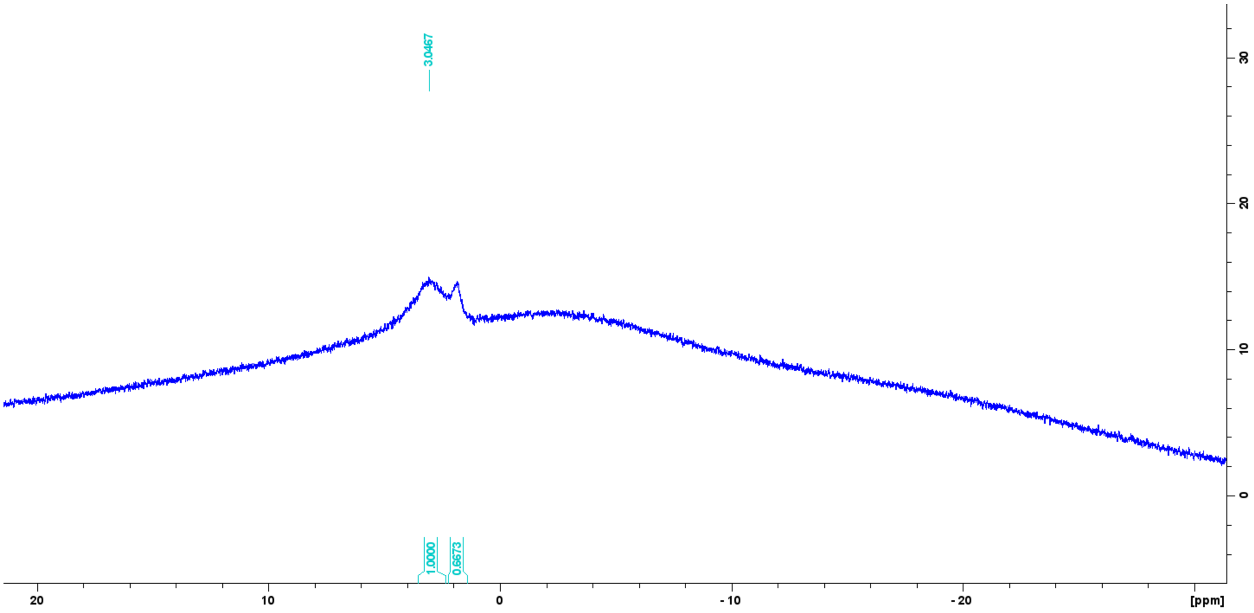

Figure S46. In Situ $^{11}B\{^{1}H\}$ NMR spectrum of the authentic BCF + PCy_3 in CD_2Cl_2. Two discrete species revealed by the $^{11}B\{^{1}H\}$ NMR spectrum, in accordance with the observed 1:1 [H-BCF][Cl]:[H-BCF][PCy_3] speciation.

In Situ $^{11}B\{^{1}H\}$ NMR spectrum of the authentic 0.5:1 BCF + GI-DPPF adduct.

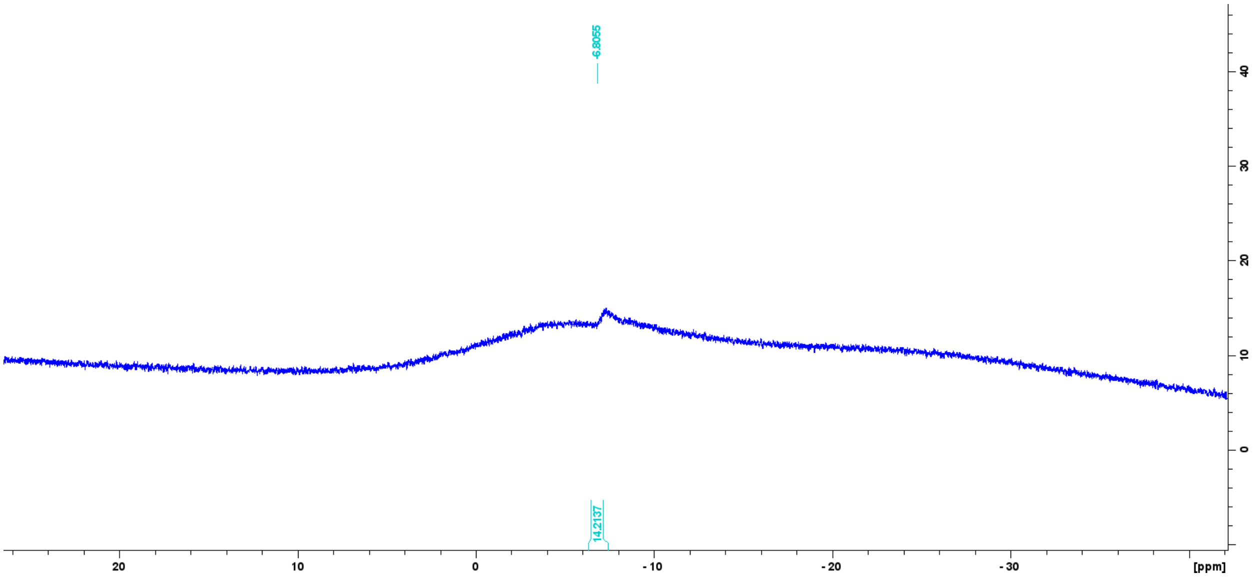

Figure S47. In Situ $^{11}B\{^{1}H\}$ NMR spectrum of the authentic 0.5 eq BCF + GI-DPPF in CD_2Cl_2. The one peak revealed by the $^{11}B\{^{1}H\}$ NMR spectrum is accordance with the Gi-BCF [BCF][Cl] speciation.

***In Situ* mass spectrometry of reaction mixture.**

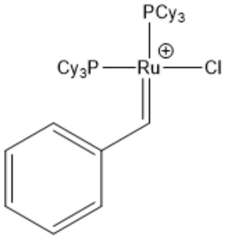

Chemical Formula: $C_{43}H_{72}ClP_2Ru$
Molecular Weight: 787.52
Minor Ion
Theoretical: 787.383579 m/z
Found: 787.383196 m/z
Δppm = 0.49

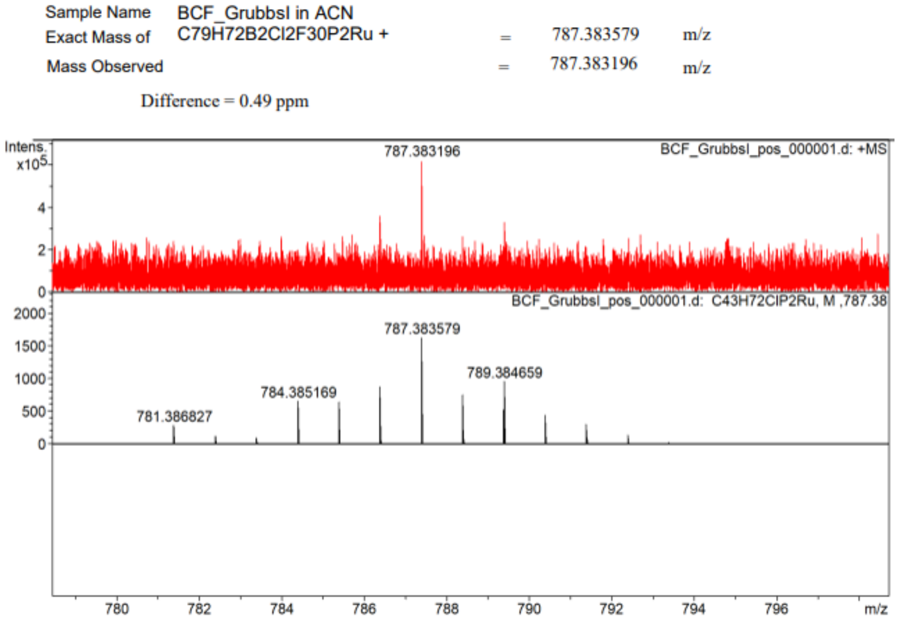

Figure S48. Mass spectrum of chloride abstracted monomeric Grubbs 1 species. High-resolution mass spectrometry data was obtained on a Bruker Daltonics 12T Apex Qe FTICR-MS using an Apollo II positive mode electrospray ionization (ESI) in picometer concentrations using MeCN as the solvent.

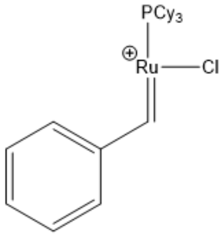

Chemical Formula: $C_{25}H_{39}ClPRu$
Molecular Weight: 507.08
Major Ion
Theoretical: 507.151591 m/z
Found: 507.151163 m/z
Δppm = 0.84

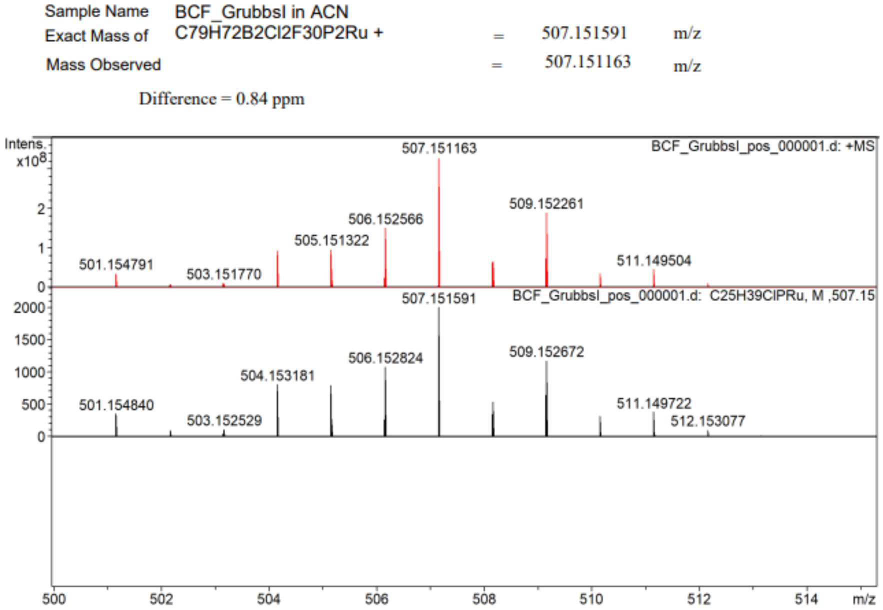

Figure S49. Mass spectrum of the chloride abstracted and phosphine dissociated monomeric Grubbs 1 species. High-resolution mass spectrometry data was obtained on a Bruker Daltonics 12T Apex Qe FTICR-MS using an Apollo II positive mode electrospray ionization (ESI) in picometer concentrations using MeCN as the solvent.

Conventional Metathesis Catalytic Cycle and Decomposition

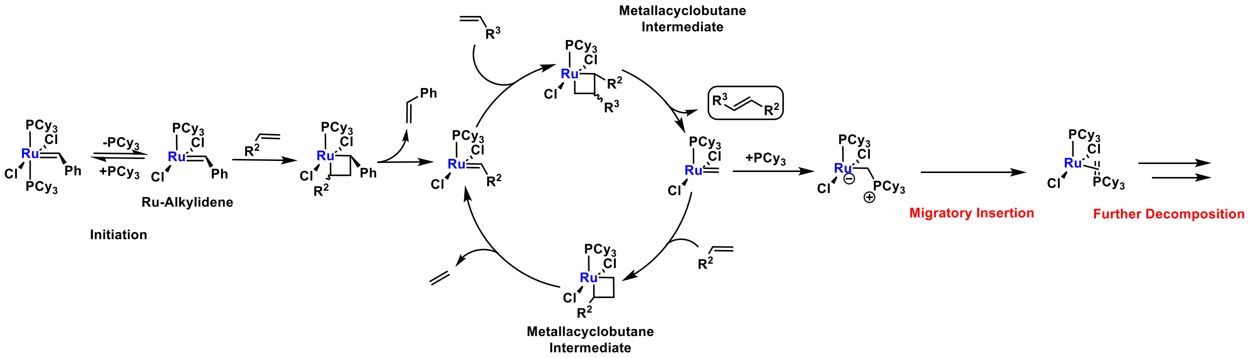

Figure S50. Conventional metathesis catalytic cycle facilitated by phosphine dissociation. Decomposition mediated by phosphine reinsertion into the pendant alkylidene.

Proposed Metathesis Catalytic Cycle via Chloride Abstraction

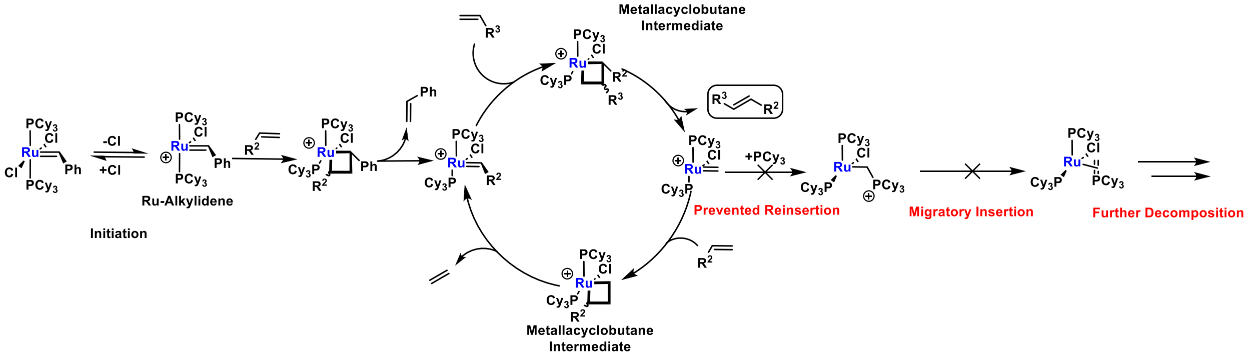

Figure S51. Modified metathesis catalytic cycle facilitated by chloride abstraction. Decomposition proposed to be reduced by disfavoring phosphine dissociation and thereby preventing the necessary reinsertion of it into the pendant alkylidene. Formal cationic ruthenium center proposed to improve olefin affinity. BCF chloride omitted for clarity but can be considered to stabilize the cationic Ru center as a weakly coordinating anion.

Alkylidene Consumption Studies

Zoomed Alkylidene Spectra

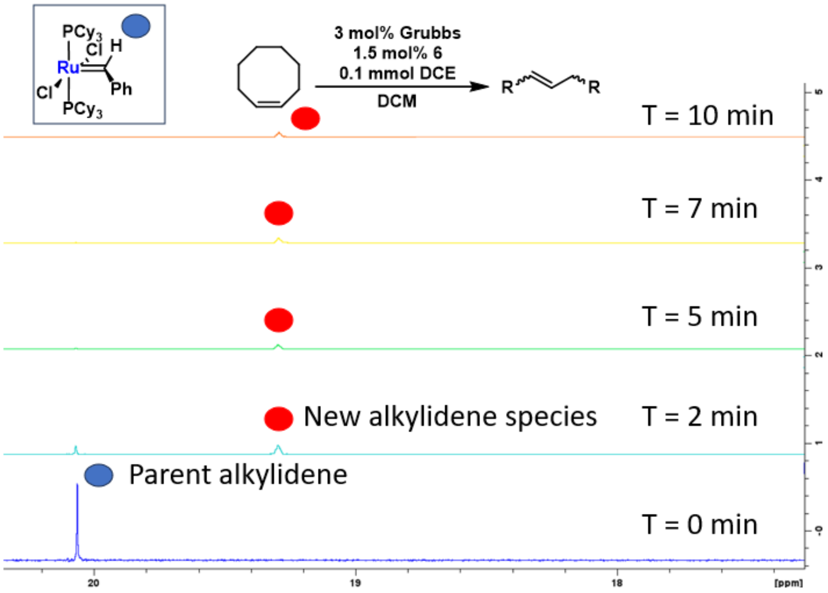

Figure S52. Experimental alkylidene consumption study to monitor catalyst resting state.

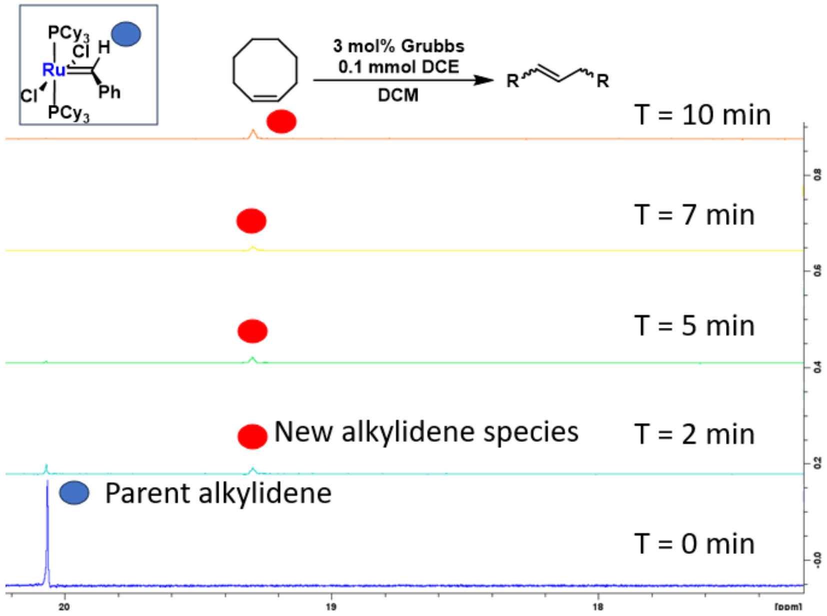

Figure S53. Control alkylidene consumption study to monitor catalyst resting state.

Alkylidene studies reveal near-immediate consumption of the parent G1 alkylidene proton resonance in both the control and the experimental reactions. This result indicated that both the

BCF-Grubbs adduct, and Grubbs 1 alone initiated at effectively the same rate. With initiation being the same, it brought us to hypothesize the increased reactivity was afforded to an increased rate of propagation of this reaction, necessarily inviting the conversion kinetic studies *vide infra*.

Conversion Studies

Kinetic Data Spectra

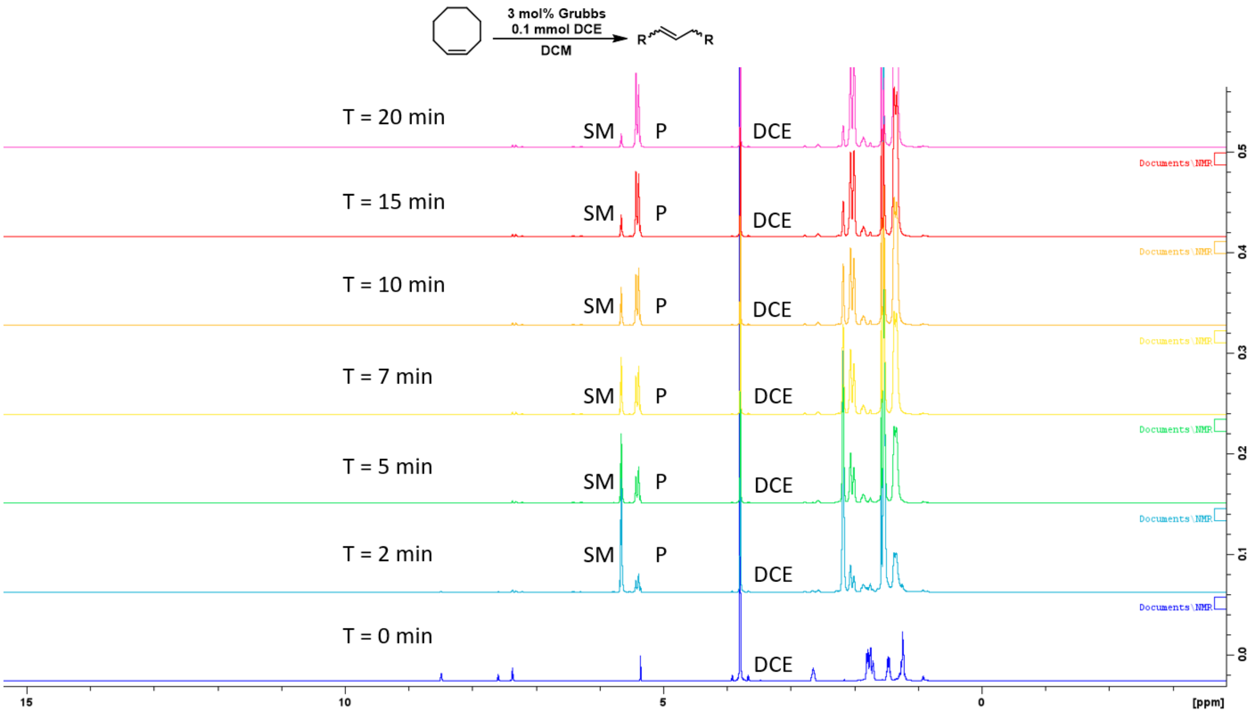

Figure S54. Control ROMP kinetic data stacked ^{1}H NMR spectra.

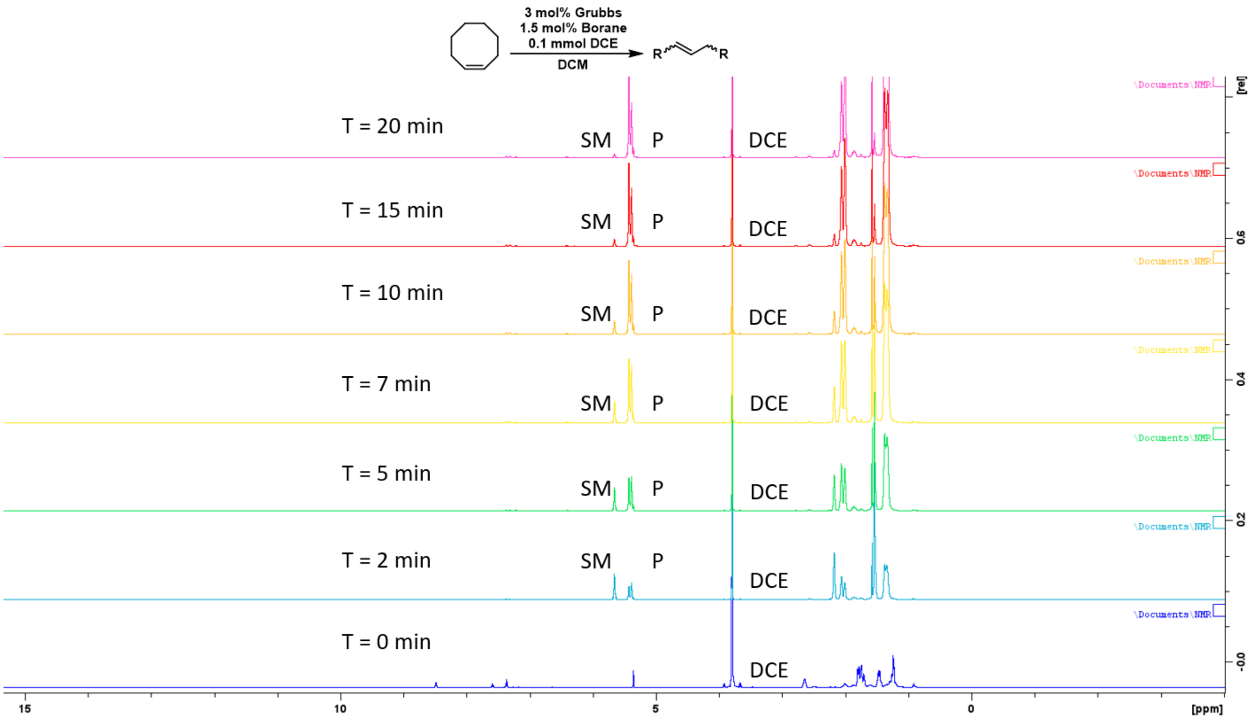

Figure S55. Catalytic ROMP kinetic data stacked ^{1}H NMR spectra.

Kinetic Data

Reaction	Time	SM Peak	Product Peak	Mass Balance	Standard Peak			LN(SM Peak)	Ln(Product Peak)
G1/BCF	0	2.1136	0	2.1136	0	G1/BCF	0	0.74839265	0
	2	0.8906	1.223	2.1136	2		2	-0.1158599	0.201306857
	5	0.5282	1.8764	2.4046	2		5	-0.6382803	0.629355047
	7	0.3093	2.0919	2.4012	2		7	-1.1734436	0.738072744
	10	0.1897	2.2462	2.4359	2		10	-1.6623114	0.8092399
	15	0.1001	2.3622	2.4623	2		15	-2.3015856	0.859593388
	20	0.057	2.4385	2.4955	2		20	-2.864704	0.891383096
G1	0	1.8169	0	1.8169	2	G1	0	0.59713175	0
	2	1.3998	0.4171	1.8169	2		2	0.33632937	-0.874429278
	5	1.0146	1.1129	2.1275	2		5	0.01449445	0.106969221
	7	0.774	1.4641	2.2381	2		7	-0.2561834	0.381240719
	10	0.5245	1.8708	2.3953	2		10	-0.6453099	0.626366147
	15	0.2929	2.1623	2.4552	2		15	-1.227924	0.77117247
	20	0.1766	2.3273	2.5039	2		20	-1.733868	0.844708797

Figure S56. Tabulated kinetic data for reaction of G1-BCF and of G1 alone. Mass balance, peaks, times, all present.

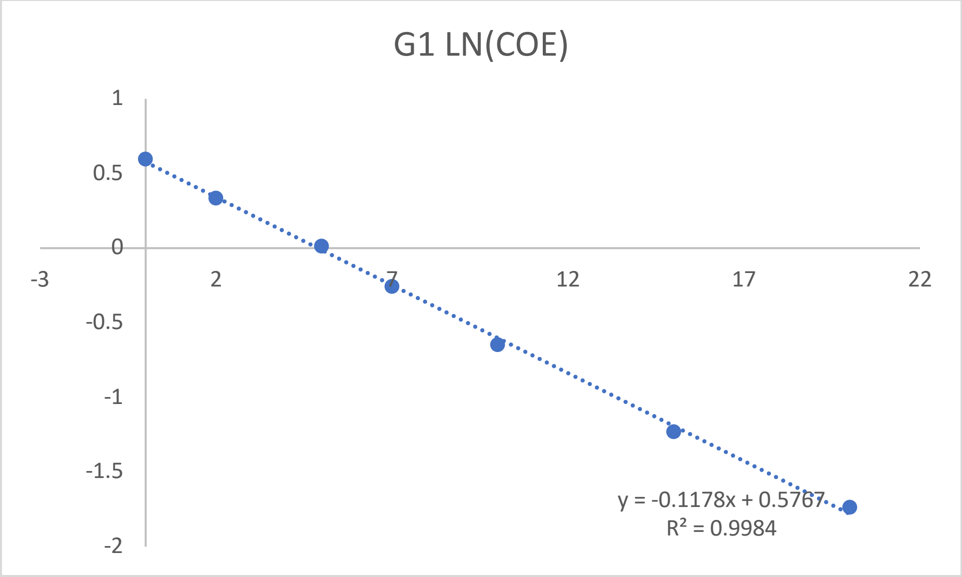

Figure S57. Plotted kinetic data of G1 alone in the ROMP of COE. R^2 = 0.9984.

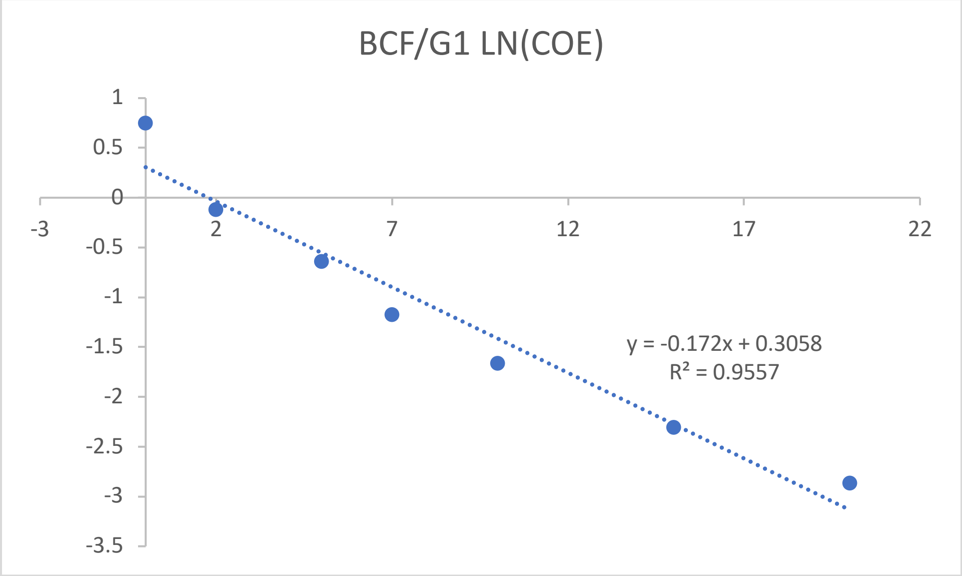

Figure S58. Plotted kinetic data of BCF-Grubbs system in the ROMP of COE. R^2 = 0.9557.

Experimental Rate Constants

G1

$\ln [A]_t / \ln [A]_o = -kt$

k = (-1.733868/0.59713175)/20

k = 0.11655

G1/BCF

$\ln [A] / \ln [A]_o = -kt$

k = (-2.864704/ 0.74839265)/20

k = 0.18065

Percent Increase in Rate = (**k**G1/BCF - **k**G1) / (**k**G1) * 100

= (0.18065 - 0.11655) / 0.11655 * 100

= 55% increase

TON

TON = mols substrate / mols catalyst

= 1.8 mmol COE / 0.003 mmol G1

TON = 600

TOF = TON/Time

$TOF_{G1/BCF}$ = TON / Time

= 600 / 20 min

$TOF_{G1/BCF}$ = 30 / min

TOF_{G1} = TON / Time

= 600 / 35 min

TOF_{G1} = 17.14 / min

GPC Data

Cyclopentene Control

Configuration

Concentration Source: RI
Flow Rate: 0.700 mL/min

Light Scattering Instrument:
Cell Type: Fused Silica
Wavelength: 658.6 nm
Calibration Constant: 2.9957×10^{-5} 1/(V cm)

RI Instrument:

Solvent: THF
Temperature Correction Enabled: yes
Refractive Index: 1.402

Processing

Collection Time: Thursday October 19, 2023 04:28:24 PM Eastern Daylight Time
Processing Time: Thursday October 19, 2023 04:53:26 PM Eastern Daylight Time

Peak settings:

**Peak Name**	Peak 1
**Peak Limits (min)**	13.357 - 16.172
**Light Scattering Model**	Zimm
**Fit Degree**	1
**dn/dc (mL/g)**	0.1850
**A2 (mol mL/g²)**	0.000

Results

Peak Results

	**Peak 1**
**Masses**	
**Injected Mass (µg)**	10.00
**Calculated Mass (µg)**	27.45
**Mass Recovery (%)**	274.5
**Mass Fraction (%)**	100.0
**Molar mass moments (g/mol)**	
**Mn**	6.402×10^{3} (±42.220%)
**Mp**	7.801×10^{3} (±27.121%)
**Mv**	n/a
**Mw**	8.814×10^{3} (±31.936%)
**Mz**	1.211×10^{4} (±71.526%)

Figure S59. Astra report of cyclopentene ROMP control.

Control

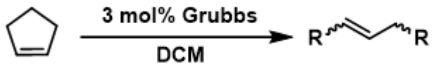

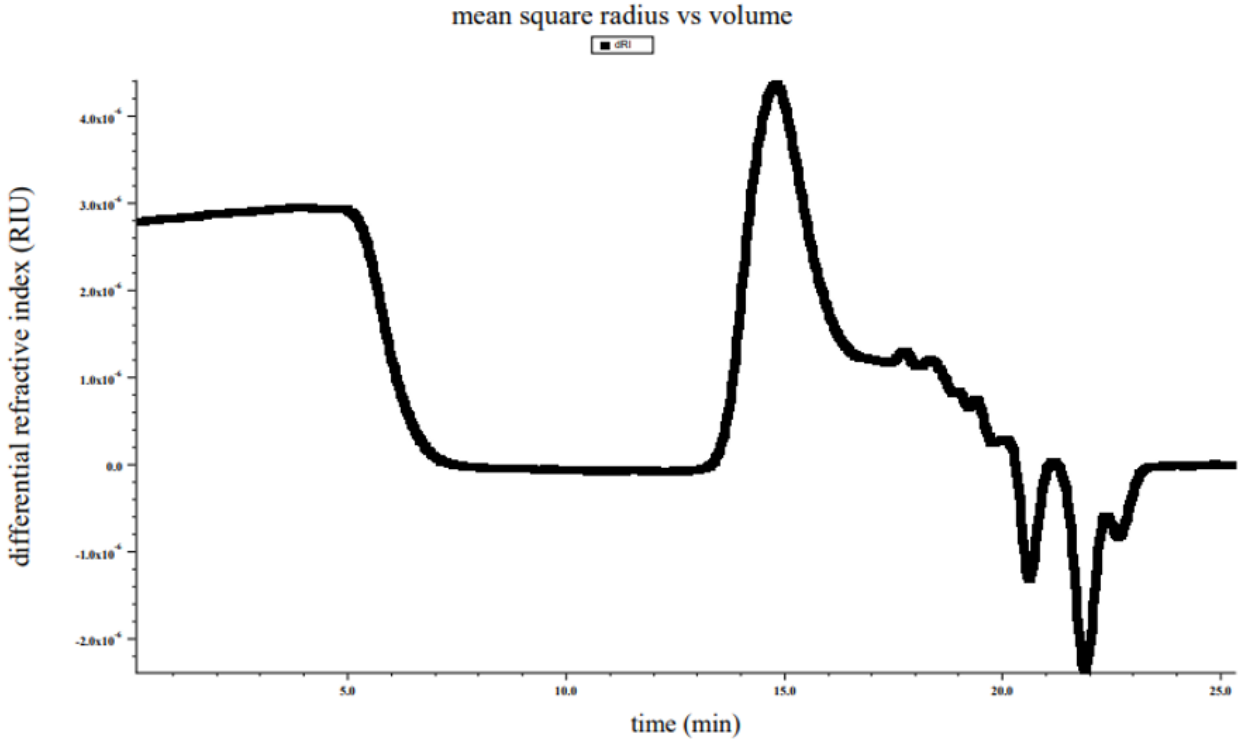

Figure S60. Refractive index (RI) of cyclopentene control reaction.

Cyclopentene BCF/G1

Configuration

Concentration Source: RI
Flow Rate: 0.700 mL/min

Light Scattering Instrument:
- **Cell Type:** Fused Silica
- **Wavelength:** 658.6 nm
- **Calibration Constant:** 2.9957×10^{-5} 1/(V cm)

RI Instrument:

Solvent: THF
- **Temperature Correction Enabled:** yes
- **Refractive Index:** 1.402

Processing

Collection Time: Thursday October 19, 2023 04:57:23 PM Eastern Daylight Time
Processing Time: Thursday October 19, 2023 05:23:14 PM Eastern Daylight Time

Peak settings:

**Peak Name**	Peak 1
**Peak Limits (min)**	11.483 - 16.056
**Light Scattering Model**	Zimm
**Fit Degree**	1
**dn/dc (mL/g)**	0.1850
**A2 (mol mL/g²)**	0.000

Results

Peak Results

	**Peak 1**
**Masses**	
**Injected Mass (μg)**	10.00
**Calculated Mass (μg)**	82.05
**Mass Recovery (%)**	820.5
**Mass Fraction (%)**	100.0
**Molar mass moments (g/mol)**	
**Mn**	3.724×10^{4} (±17.432%)
**Mp**	5.884×10^{4} (±1.967%)
**Mv**	n/a
**Mw**	5.966×10^{4} (±3.732%)
**Mz**	7.956×10^{4} (±7.543%)

Figure S61. Astra report of G1-BCF ROMP of cyclopentene.

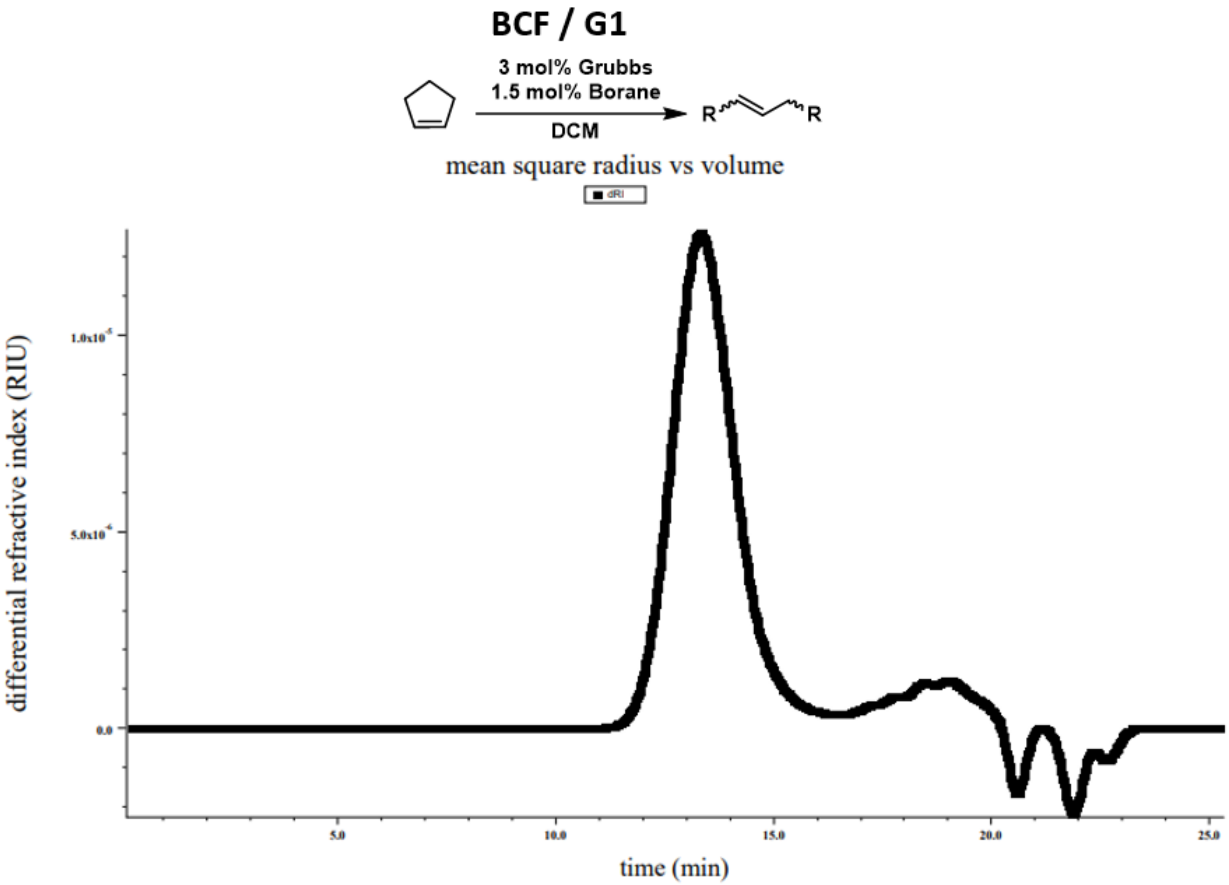

Figure S62. Refractive index (RI) of BCF-G1 catalyzed ROMP of cyclopentene.

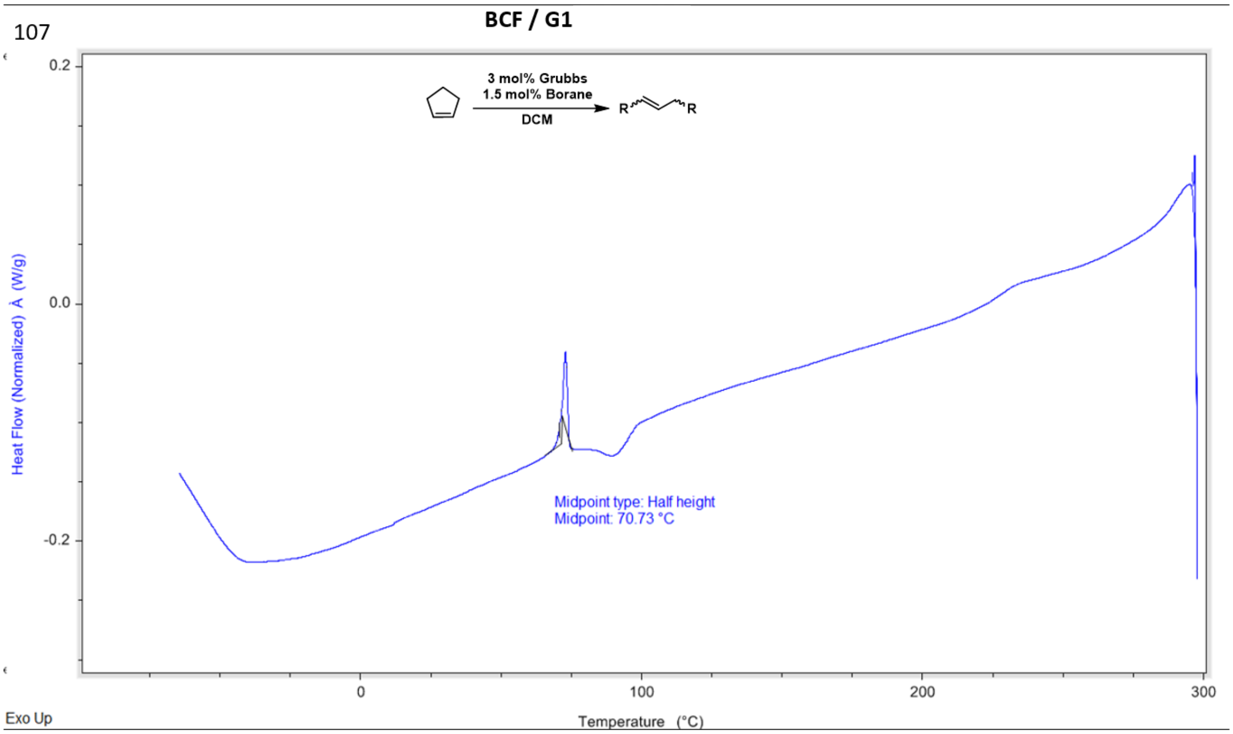

Figure S63. Glass transition point of G1-BCF catalyzed ROMP of cyclopentene obtained by differential scanning calorimetry (DSC).

Cyclopentene Cp-BCF / G1

Configuration

Concentration Source: RI
Flow Rate: 0.700 mL/min

Light Scattering Instrument:
- **Cell Type:** Fused Silica
- **Wavelength:** 658.6 nm
- **Calibration Constant:** 2.9957×10^{-5} 1/(V cm)

RI Instrument:

Solvent: THF
- **Temperature Correction Enabled:** yes
- **Refractive Index:** 1.402

Processing

Collection Time: Thursday November 02, 2023 12:59:31 PM Eastern Daylight Time
Processing Time: Thursday November 02, 2023 01:24:32 PM Eastern Daylight Time

Peak settings:

**Peak Name**	Peak 1
**Peak Limits (min)**	12.050 - 15.153
**Light Scattering Model**	Zimm
**Fit Degree**	1
**dn/dc (mL/g)**	0.1850
**A2 (mol mL/g²)**	0.000

Results

Peak Results

	**Peak 1**
**Masses**	
**Injected Mass (µg)**	10.00
**Calculated Mass (µg)**	91.34
**Mass Recovery (%)**	913.4
**Mass Fraction (%)**	100.0
**Molar mass moments (g/mol)**	
**Mn**	3.282×10^{4} (±15.862%)
**Mp**	4.118×10^{4} (±3.009%)
**Mv**	n/a
**Mw**	4.641×10^{4} (±5.694%)
**Mz**	6.243×10^{4} (±11.978%)

Figure S64. Astra report of cp-BCF-G1 catalyzed ROMP of cyclopentene.

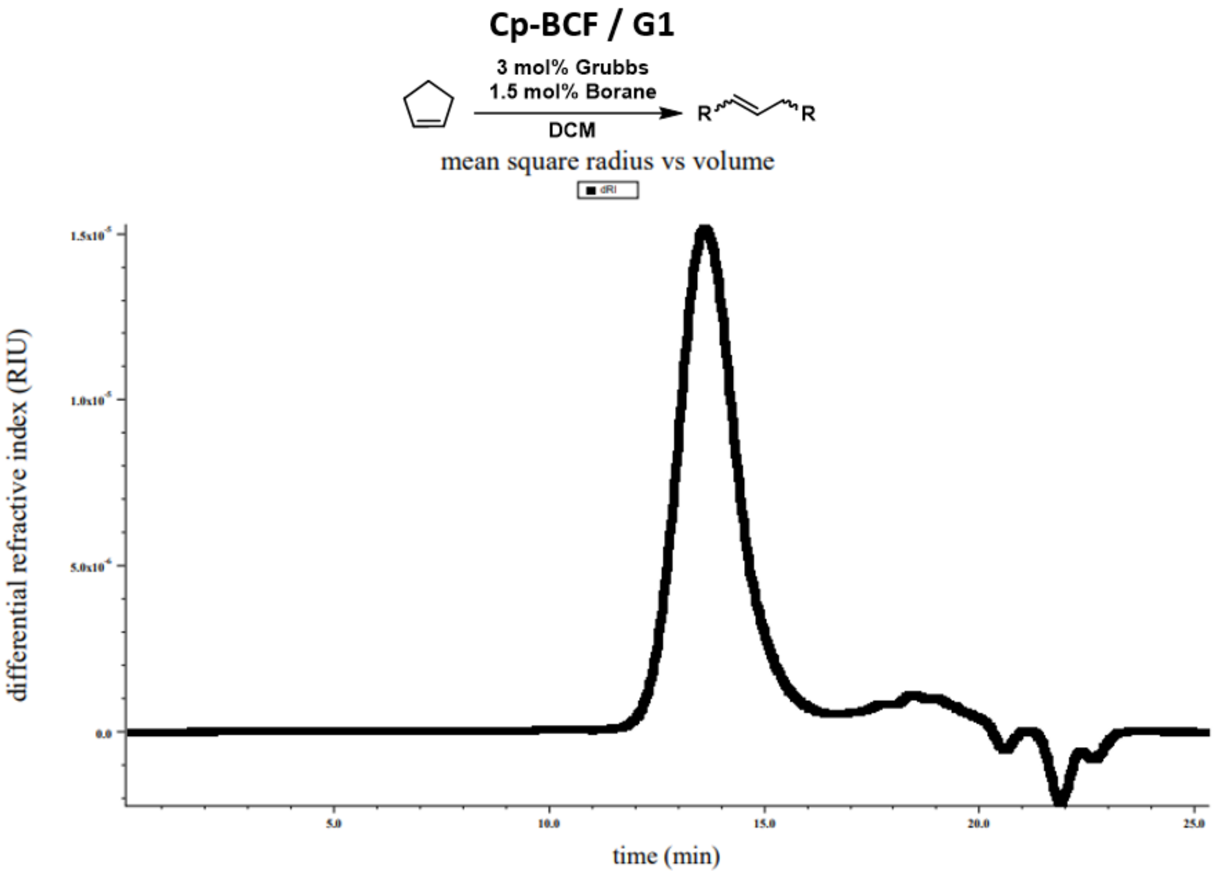

Figure S65. Refractive index of cp-BCF-G1 catalyzed ROMP of cyclopentene.

119

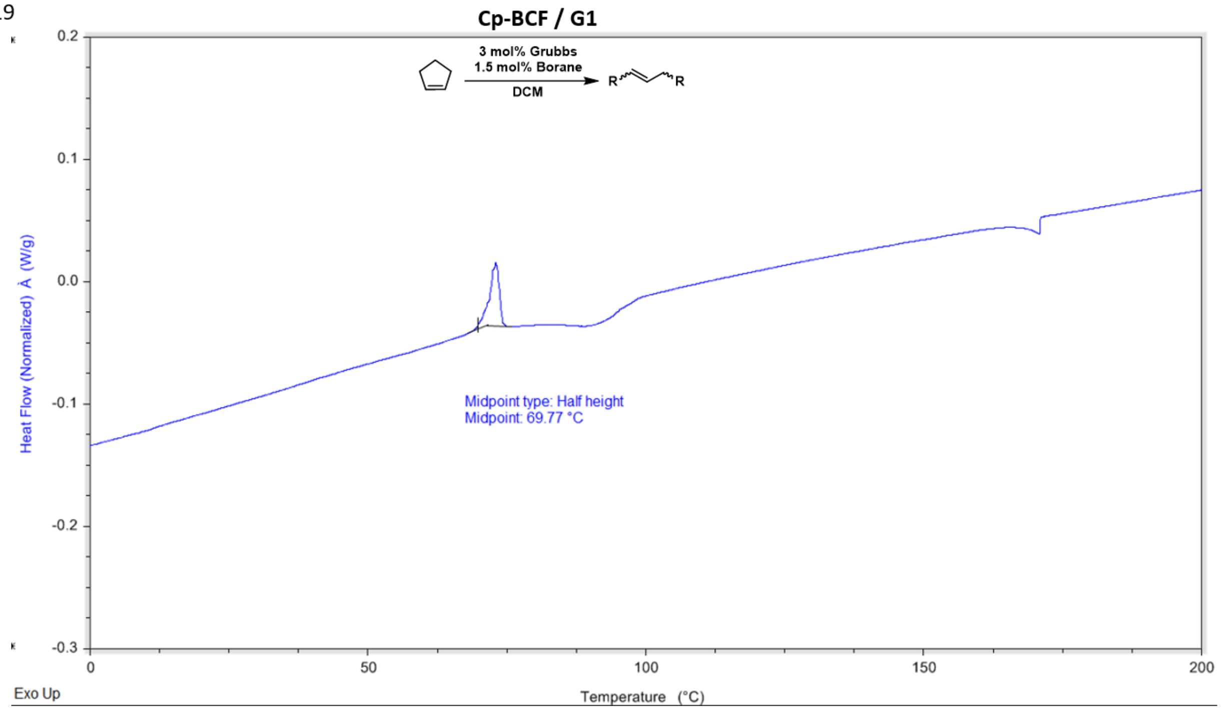

Figure S66.Glass transition point of Cp-BCF-G1 catalyzed ROMP of cyclopentene obtained by differential scanning calorimetry (DSC).

Cyclopentene H-BCF/G1

Configuration

Concentration Source: RI
Flow Rate: 0.700 mL/min

Light Scattering Instrument:
Cell Type: Fused Silica
Wavelength: 658.6 nm
Calibration Constant: 2.9957×10^{-5} 1/(V cm)

RI Instrument:

Solvent: THF
Temperature Correction Enabled: yes
Refractive Index: 1.402

Processing

Collection Time: Thursday November 02, 2023 12:28:52 PM Eastern Daylight Time
Processing Time: Thursday November 02, 2023 12:53:54 PM Eastern Daylight Time

Peak settings:

Peak Name	Peak 1
Peak Limits (min)	10.967 - 14.843
Light Scattering Model	Zimm
Fit Degree	1
dn/dc (mL/g)	0.1850
A2 (mol mL/g²)	0.000

Results

Peak Results

	Peak 1
**Masses**	
Injected Mass (µg)	10.00
Calculated Mass (µg)	21.47
Mass Recovery (%)	214.7
Mass Fraction (%)	100.0
**Molar mass moments (g/mol)**	
Mn	5.163×10^{4} (±32.262%)
Mp	4.520×10^{4} (±20.574%)
Mv	n/a
Mw	6.251×10^{4} (±28.693%)
Mz	7.570×10^{4} (±63.517%)

Figure S67. Astra report of H-BCF-G1 catalyzed ROMP of cyclopentene.

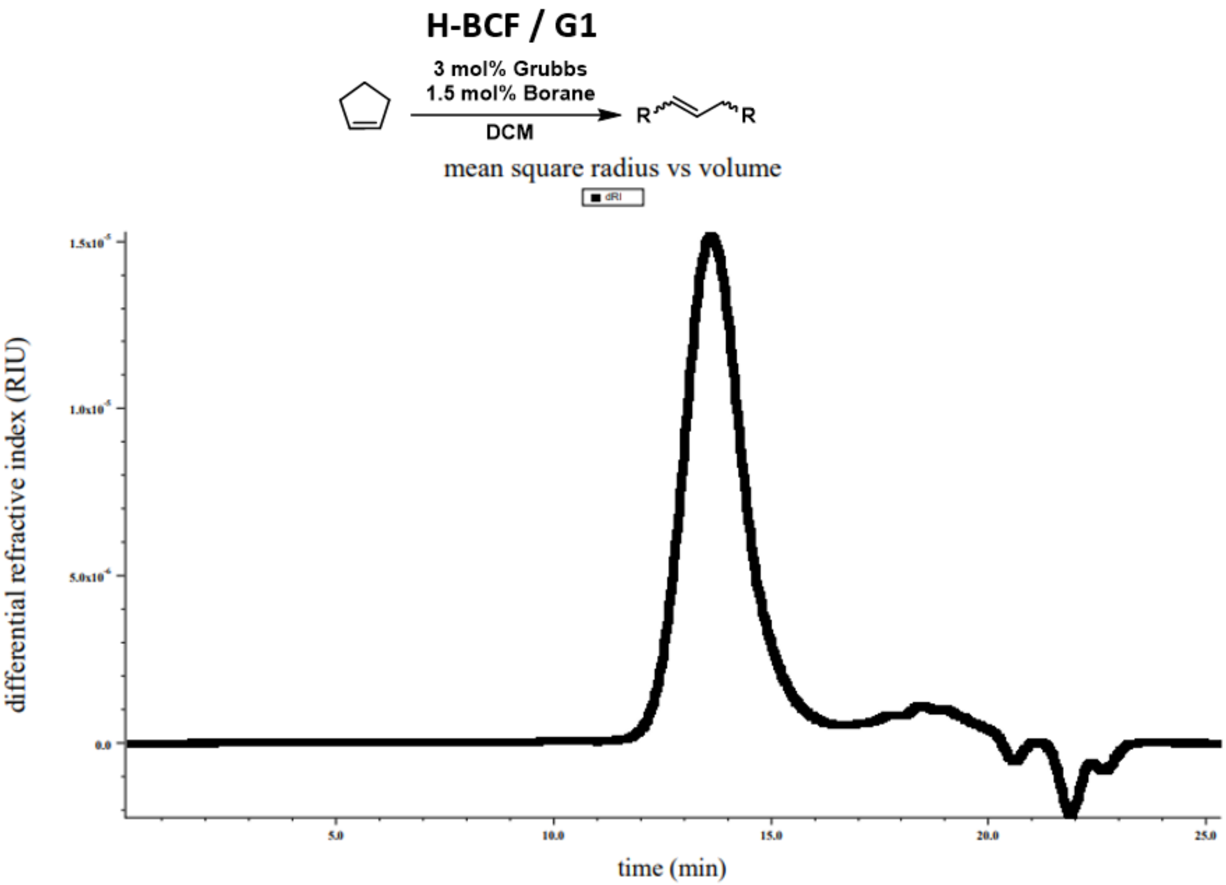

Figure S68. Refractive index of H-BCF-G1 catalyzed ROMP of cyclopentene.

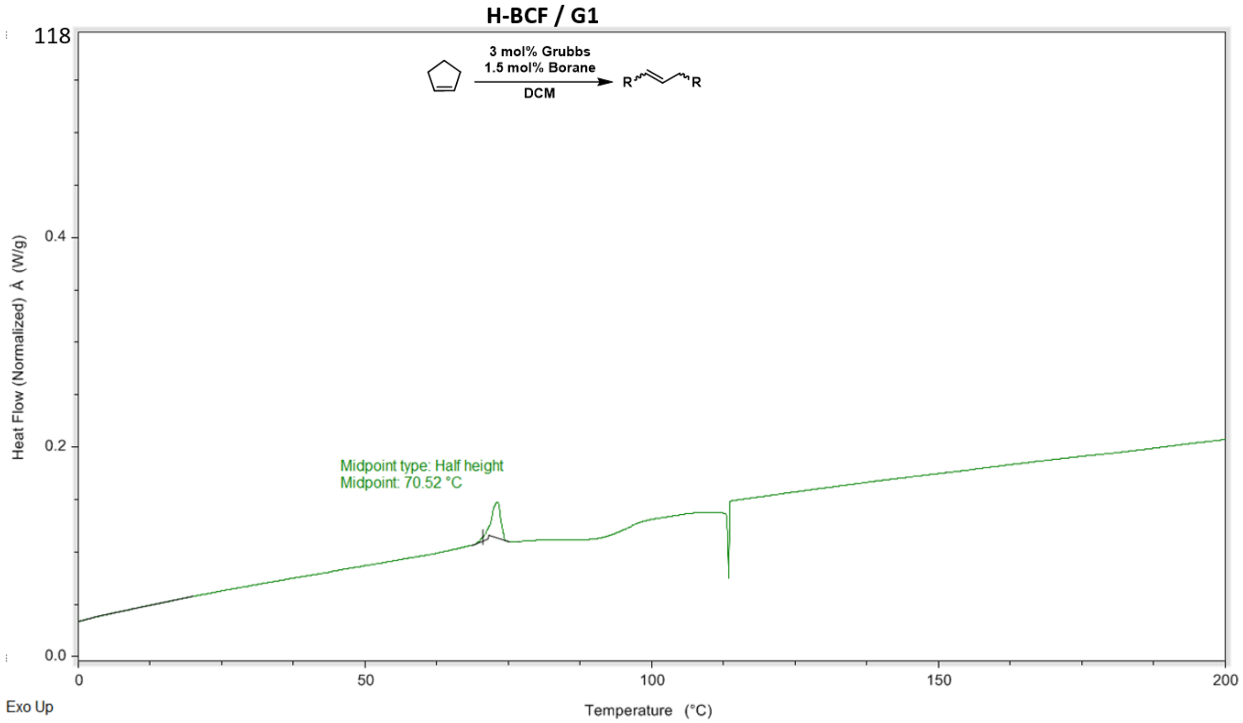

Figure S69.Glass transition point of H-BCF-G1 catalyzed ROMP of cyclopentene obtained by differential scanning calorimetry (DSC).

Cyclopentene NaBArF/G1

Configuration

Concentration Source: RI
Flow Rate: 0.700 mL/min

Light Scattering Instrument:
- **Cell Type:** Fused Silica
- **Wavelength:** 658.6 nm
- **Calibration Constant:** 2.9957×10^{-5} 1/(V cm)

RI Instrument:

Solvent: THF
- **Temperature Correction Enabled:** yes
- **Refractive Index:** 1.402

Processing

Collection Time: Friday February 23, 2024 11:15:00 AM Eastern Standard Time
Processing Time: Friday February 23, 2024 11:40:01 AM Eastern Standard Time

Peak settings:

**Peak Name**	Peak 1
**Peak Limits (min)**	11.654 - 17.847
**Light Scattering Model**	Zimm
**Fit Degree**	1
**dn/dc (mL/g)**	0.1850
**A2 (mol mL/g²)**	0.000

Results

Peak Results

	**Peak 1**
**Masses**	
**Injected Mass (μg)**	10.00
**Calculated Mass (μg)**	129.99
**Mass Recovery (%)**	1299.9
**Mass Fraction (%)**	100.0
**Molar mass moments (g/mol)**	
**Mn**	9.905×10^{3} (±37.403%)
**Mp**	7.837×10^{3} (±19.296%)
**Mv**	n/a
**Mw**	1.662×10^{4} (±21.092%)
**Mz**	6.634×10^{4} (±49.616%)

(Ctrl)

Figure S70. Astra report of NaBArF-G1 catalyzed ROMP of cyclopentene.

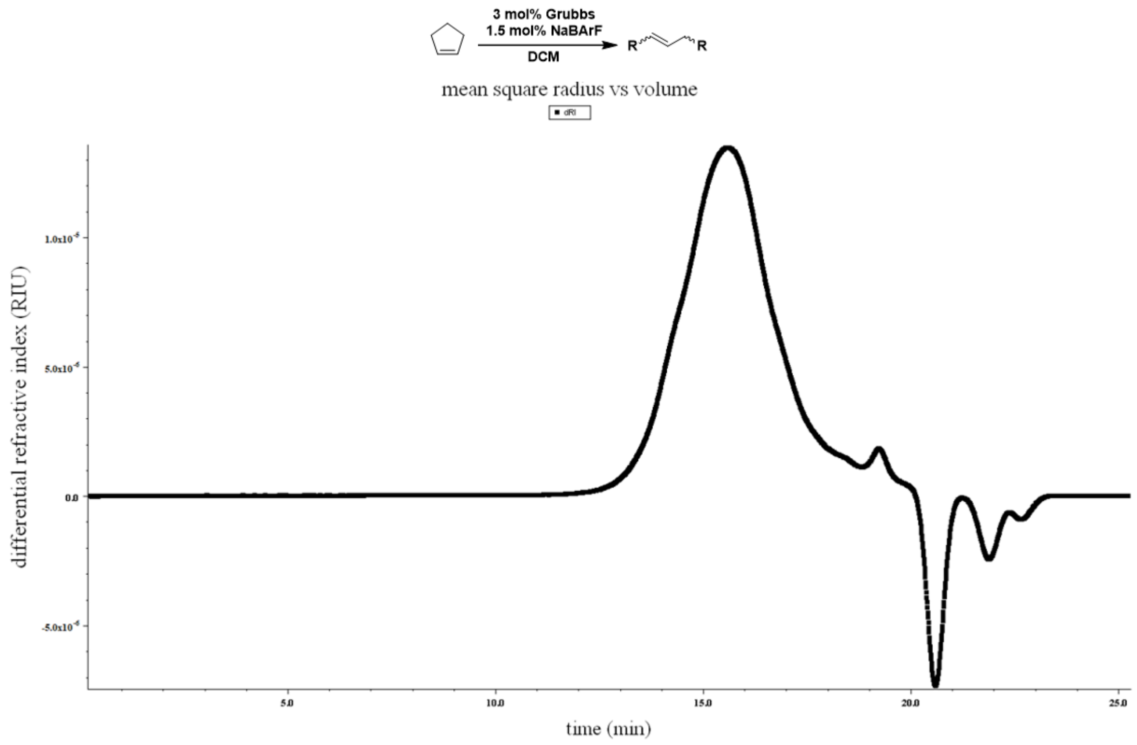

Figure S71. Refractive index (RI) of NaBArF-G1 catalyzed ROMP of cyclopentene.

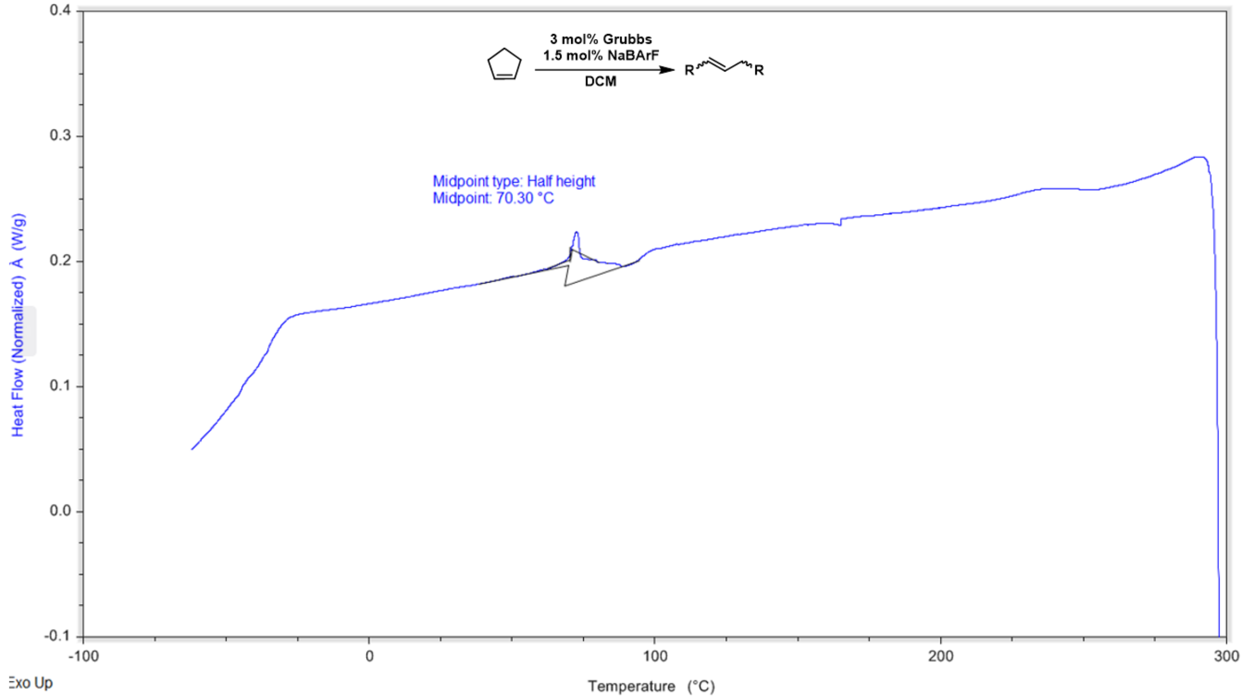

Figure S72. Glass transition point of G1-NaBArF catalyzed ROMP of cyclopentene obtained by differential scanning calorimetry (DSC).

Cyclooctene Control

Configuration

Concentration Source: RI
Flow Rate: 0.700 mL/min

Light Scattering Instrument:

Cell Type: Fused Silica
Wavelength: 658.6 nm
Calibration Constant: 2.9957×10^{-5} 1/(V cm)

RI Instrument:

Solvent: THF

Temperature Correction Enabled: yes
Refractive Index: 1.402

Processing

Collection Time: Tuesday October 03, 2023 01:18:32 PM Eastern Daylight Time
Processing Time: Tuesday October 03, 2023 01:43:33 PM Eastern Daylight Time

Peak settings:

**Peak Name**	Peak 1
**Peak Limits (min)**	11.980 - 15.383
**Light Scattering Model**	Zimm
**Fit Degree**	1
**dn/dc (mL/g)**	0.1850
**A2 (mol mL/g²)**	0.000

Results

Peak Results

	**Peak 1**
**Masses**	
**Injected Mass (µg)**	10.00
**Calculated Mass (µg)**	172.06
**Mass Recovery (%)**	1720.6
**Mass Fraction (%)**	100.0
**Molar mass moments (g/mol)**	
**Mn**	2.639×10^{4} (±8.214%)
**Mp**	3.172×10^{4} (±1.846%)
**Mv**	n/a
**Mw**	3.541×10^{4} (±3.754%)
**Mz**	4.727×10^{4} (±9.716%)

Figure S73. Astra report of G1 catalyzed ROMP of cyclooctene.

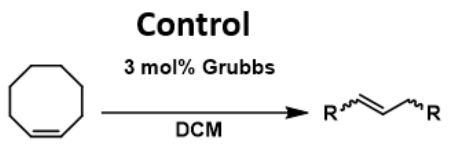

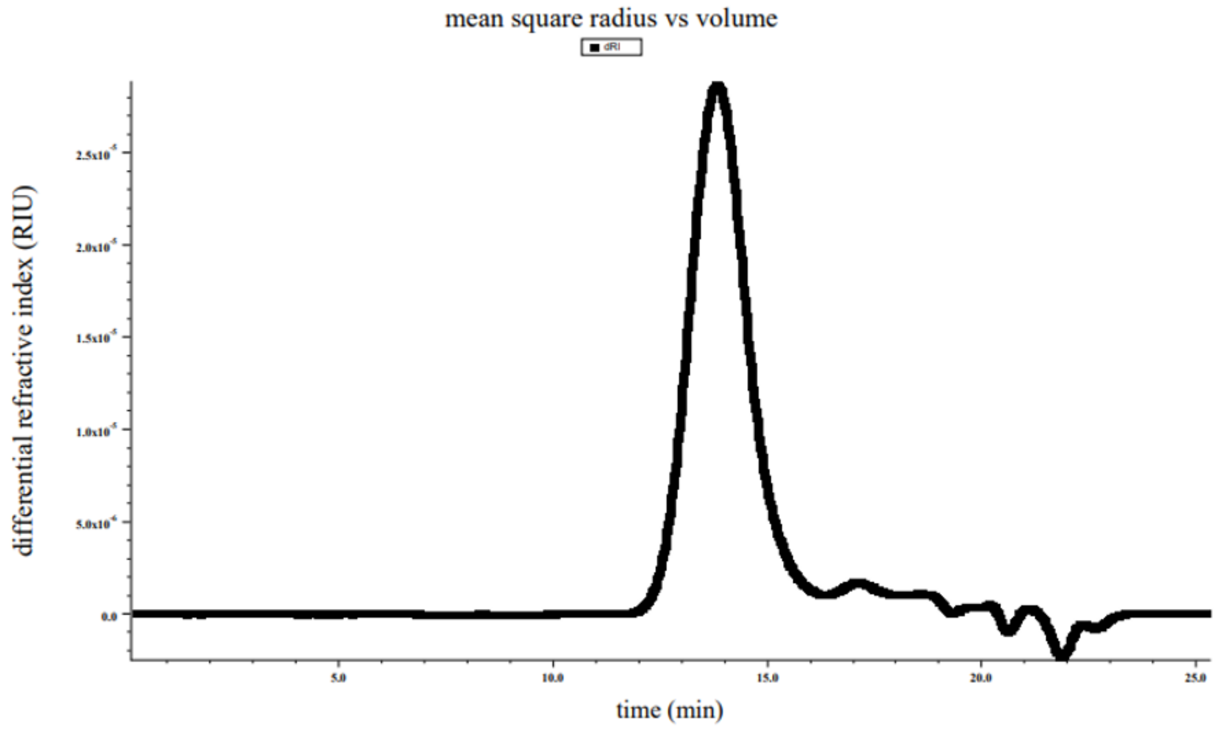

Figure S74. Refractive index of G1 catalyzed ROMP of COE.

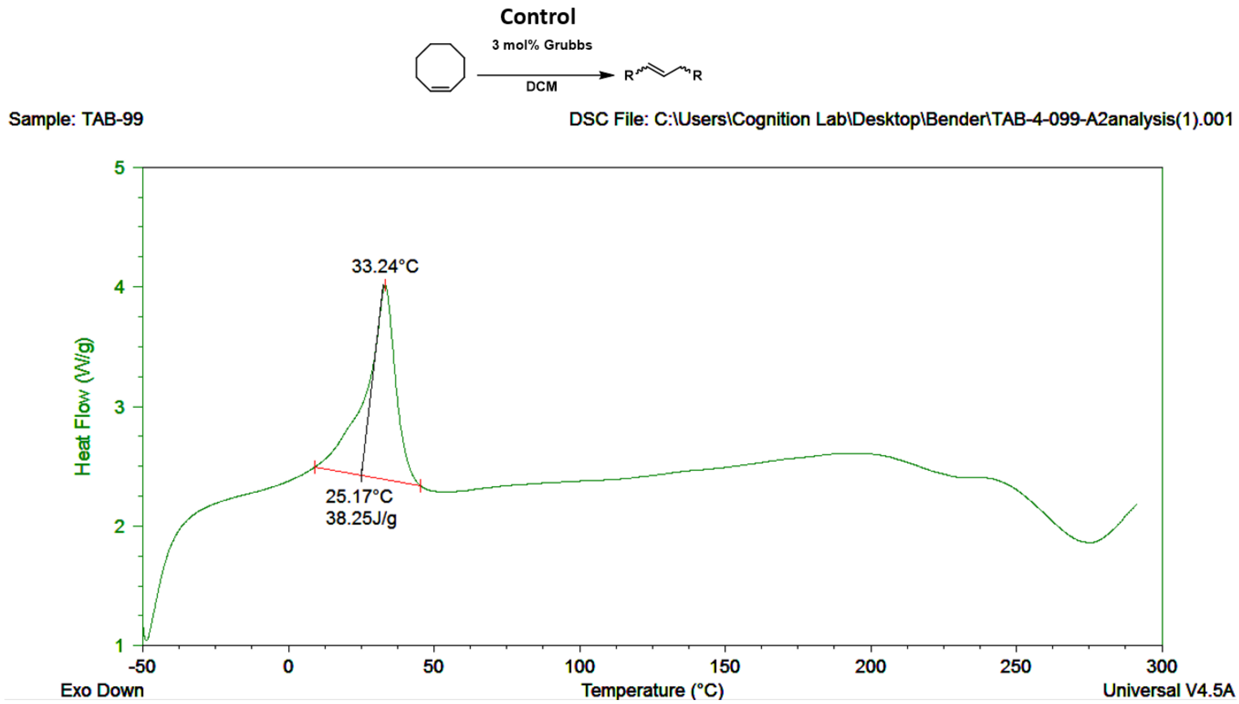

Figure S75. Glass transition point of G1-BCF catalyzed ROMP of COE obtained by differential scanning calorimetry (DSC).

Cyclooctene BCF/G1

Configuration

Concentration Source: RI
Flow Rate: 0.700 mL/min

Light Scattering Instrument:
- **Cell Type:** Fused Silica
- **Wavelength:** 658.6 nm
- **Calibration Constant:** 2.9957×10^{-5} 1/(V cm)

RI Instrument:

Solvent: THF
- **Temperature Correction Enabled:** yes
- **Refractive Index:** 1.402

Processing

Collection Time: Thursday October 05, 2023 04:33:10 PM Eastern Daylight Time
Processing Time: Thursday October 05, 2023 04:58:11 PM Eastern Daylight Time

Peak settings:

**Peak Name**	Peak 1
**Peak Limits (min)**	12.079 - 15.106
**Light Scattering Model**	Zimm
**Fit Degree**	1
**dn/dc (mL/g)**	0.1850
**A2 (mol mL/g²)**	0.000

Results

Peak Results

	**Peak 1**
**Masses**	
**Injected Mass (μg)**	10.00
**Calculated Mass (μg)**	45.22
**Mass Recovery (%)**	452.2
**Mass Fraction (%)**	100.0
**Molar mass moments (g/mol)**	
**Mn**	2.652×10^4 (±21.482%)
**Mp**	3.077×10^4 (±7.971%)
**Mv**	n/a
**Mw**	3.309×10^4 (±13.193%)
**Mz**	4.024×10^4 (±28.571%)

Figure S76. Astra report of BCF/G1 catalyzed ROMP of COE.

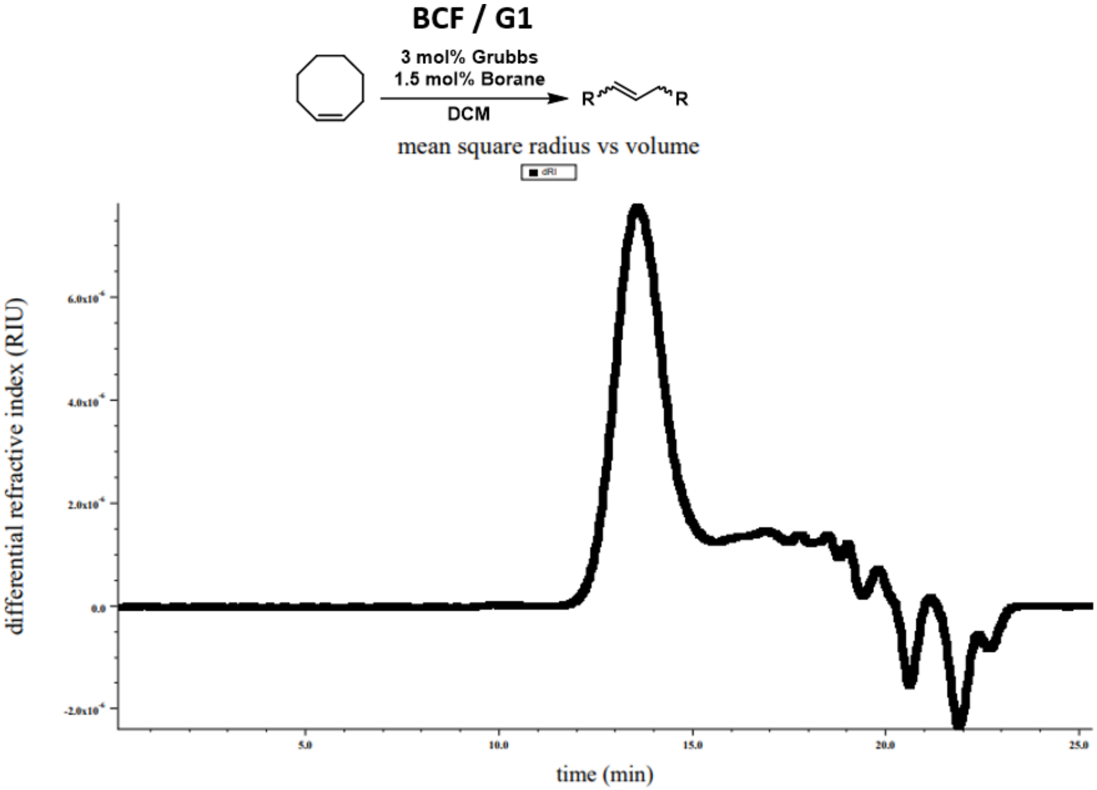

Figure S77. Refractive index of BCF/G1 catalyzed ROMP of COE.

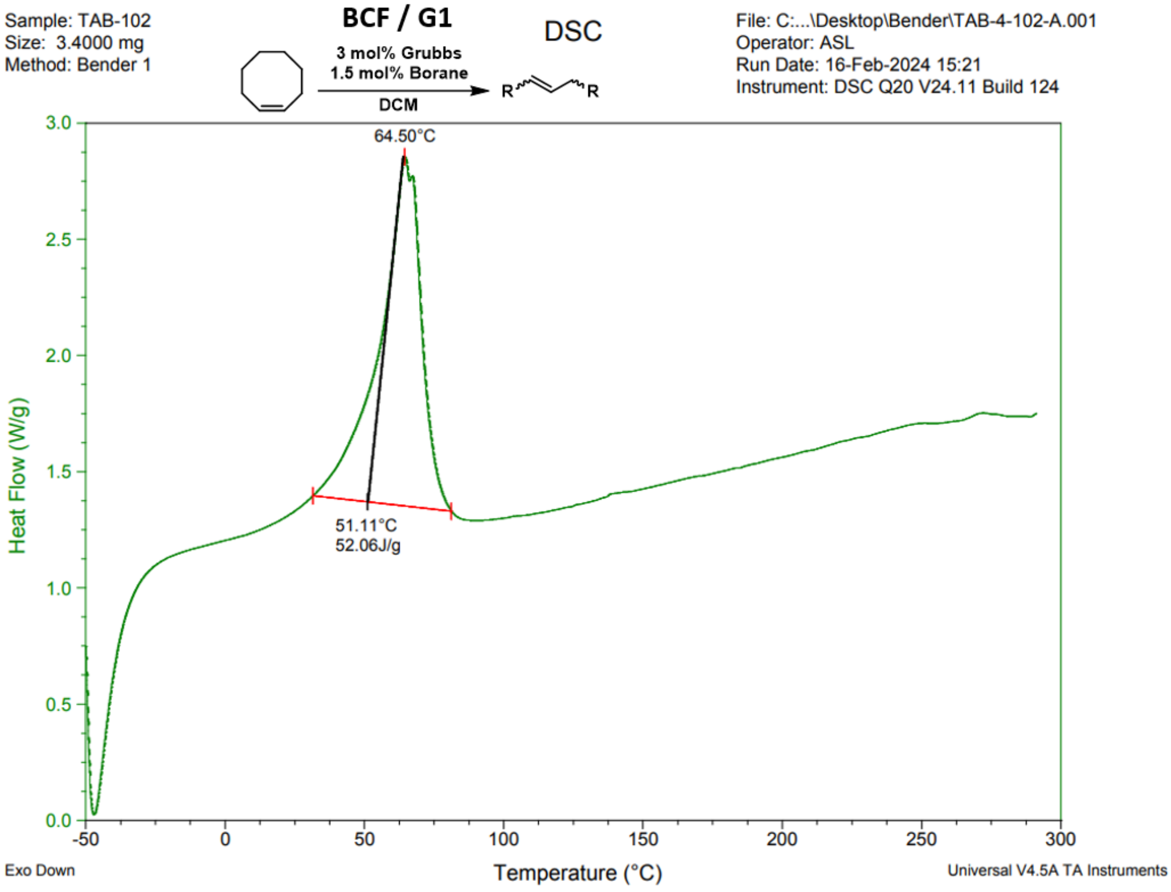

Figure S78. Glass transition point of G1-BCF catalyzed ROMP of COE obtained by differential scanning calorimetry (DSC).

Cyclooctene Cp-BCF/G1

Configuration

Concentration Source: RI
Flow Rate: 0.700 mL/min

Light Scattering Instrument:
- **Cell Type:** Fused Silica
- **Wavelength:** 658.6 nm
- **Calibration Constant:** 2.9957×10^{-5} 1/(V cm)

RI Instrument:

Solvent: THF
- **Temperature Correction Enabled:** yes
- **Refractive Index:** 1.402

Processing

Collection Time: Tuesday October 03, 2023 11:35:25 AM Eastern Daylight Time
Processing Time: Tuesday October 03, 2023 12:00:26 PM Eastern Daylight Time

Peak settings:

**Peak Name**	Peak 1
**Peak Limits (min)**	12.267 - 15.619
**Light Scattering Model**	Zimm
**Fit Degree**	1
**dn/dc (mL/g)**	0.1850
**A2 (mol mL/g²)**	0.000

Results

Peak Results

	**Peak 1**
**Masses**	
**Injected Mass (µg)**	10.00
**Calculated Mass (µg)**	92.53
**Mass Recovery (%)**	925.3
**Mass Fraction (%)**	100.0
**Molar mass moments (g/mol)**	
**Mn**	2.170×10^{4} (±15.759%)
**Mp**	2.464×10^{4} (±4.483%)
**Mv**	n/a
**Mw**	2.854×10^{4} (±9.424%)
**Mz**	3.876×10^{4} (±23.071%)

Figure S79. Astra report of Cp-BCF-G1 catalyzed ROMP of COE.

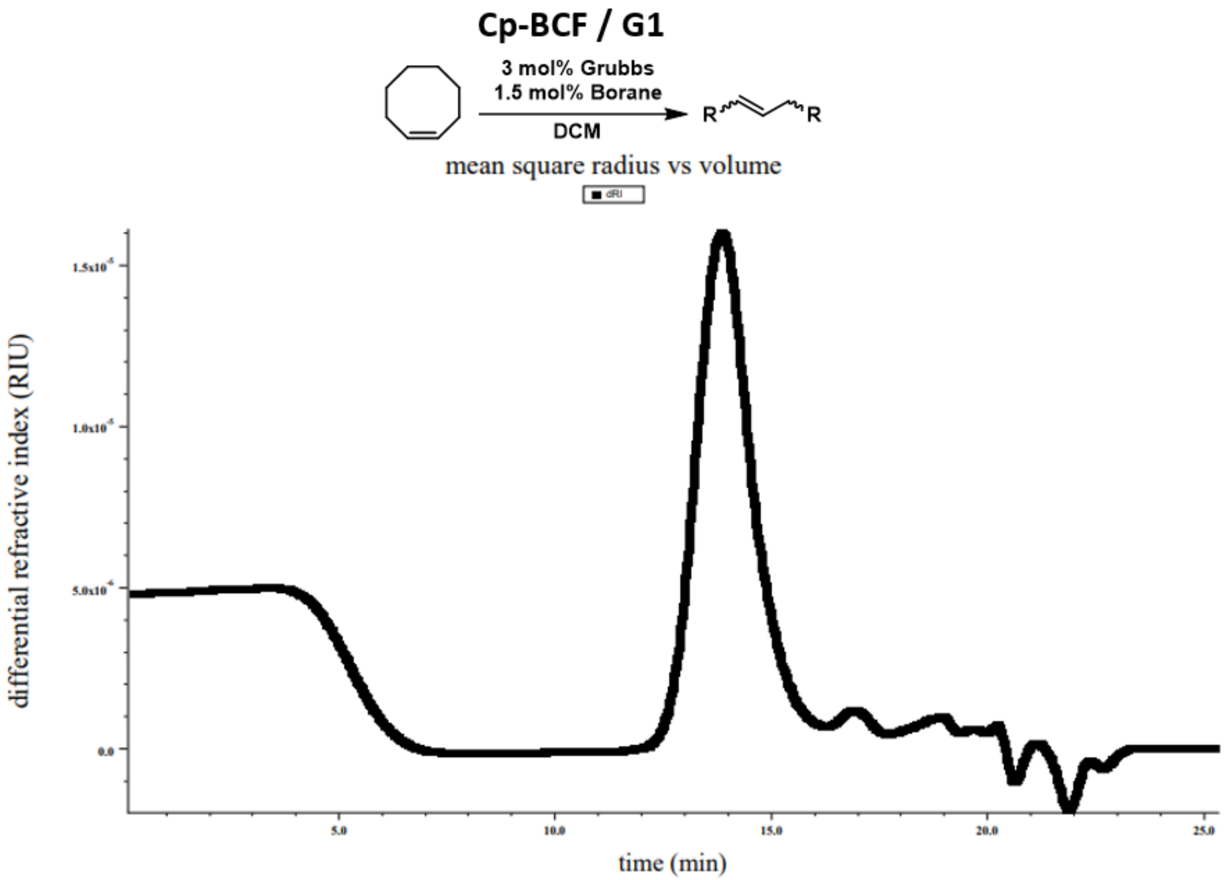

Figure S80. Refractive index of Cp-BCF-G1 catalyzed ROMP of COE.

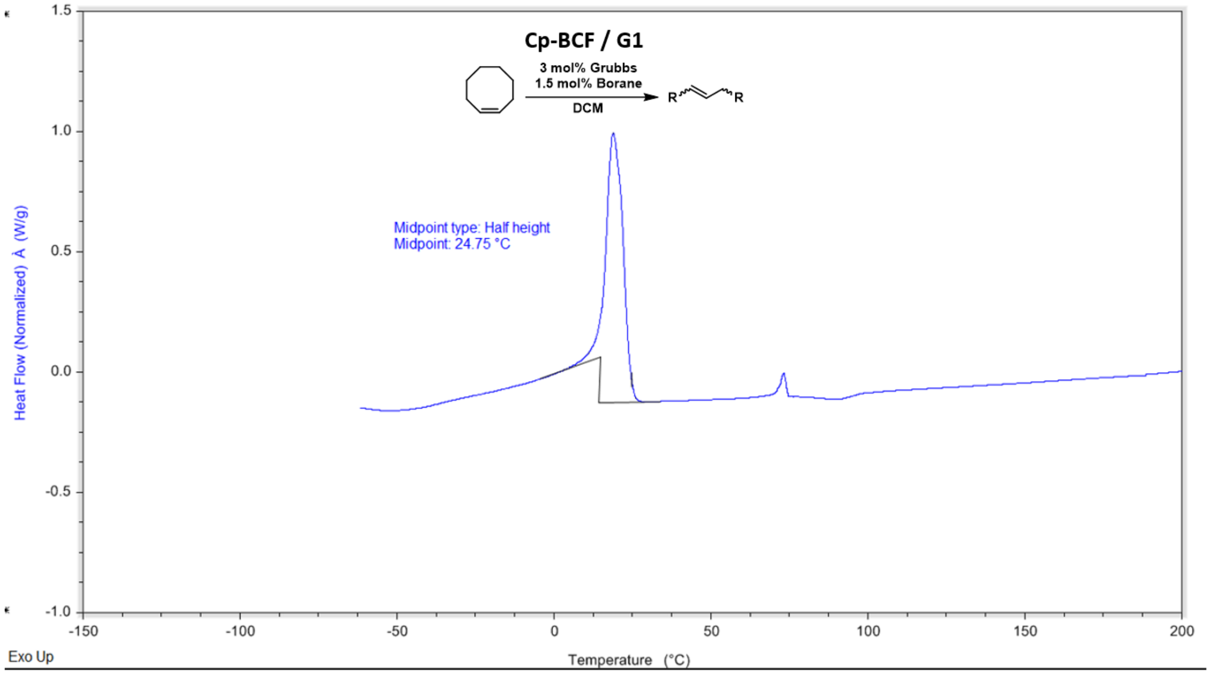

Figure S81. Glass transition point of Cp-BCF-G1 catalyzed ROMP of COE obtained by differential scanning calorimetry (DSC).

Cyclooctene H-BCF/G1

Configuration

Concentration Source: RI
Flow Rate: 0.700 mL/min

Light Scattering Instrument:
Cell Type: Fused Silica
Wavelength: 658.6 nm
Calibration Constant: 2.9957×10^{-5} 1/(V cm)

RI Instrument:

Solvent: THF
Temperature Correction Enabled: yes
Refractive Index: 1.402

Processing

Collection Time: Thursday October 05, 2023 03:35:29 PM Eastern Daylight Time
Processing Time: Thursday October 05, 2023 04:01:36 PM Eastern Daylight Time

Peak settings:

**Peak Name**	Peak 1
**Peak Limits (min)**	12.092 - 15.182
**Light Scattering Model**	Zimm
**Fit Degree**	1
**dn/dc (mL/g)**	0.1850
**A2 (mol mL/g²)**	0.000

Results

Peak Results

	**Peak 1**
**Masses**	
**Injected Mass (μg)**	10.00
**Calculated Mass (μg)**	40.44
**Mass Recovery (%)**	404.4
**Mass Fraction (%)**	100.0
**Molar mass moments (g/mol)**	
**Mn**	3.302×10^{4} (±22.030%)
**Mp**	3.428×10^{4} (±8.078%)
**Mv**	n/a
**Mw**	4.300×10^{4} (±16.702%)
**Mz**	5.989×10^{4} (±40.833%)

Figure S82. Astra report of H-BCF-G1 catalyzed ROMP of COE.

H-BCF

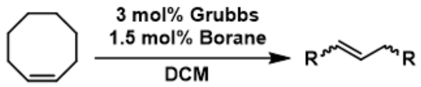

TAB4-100-A: Graph (mean square radius vs volume)

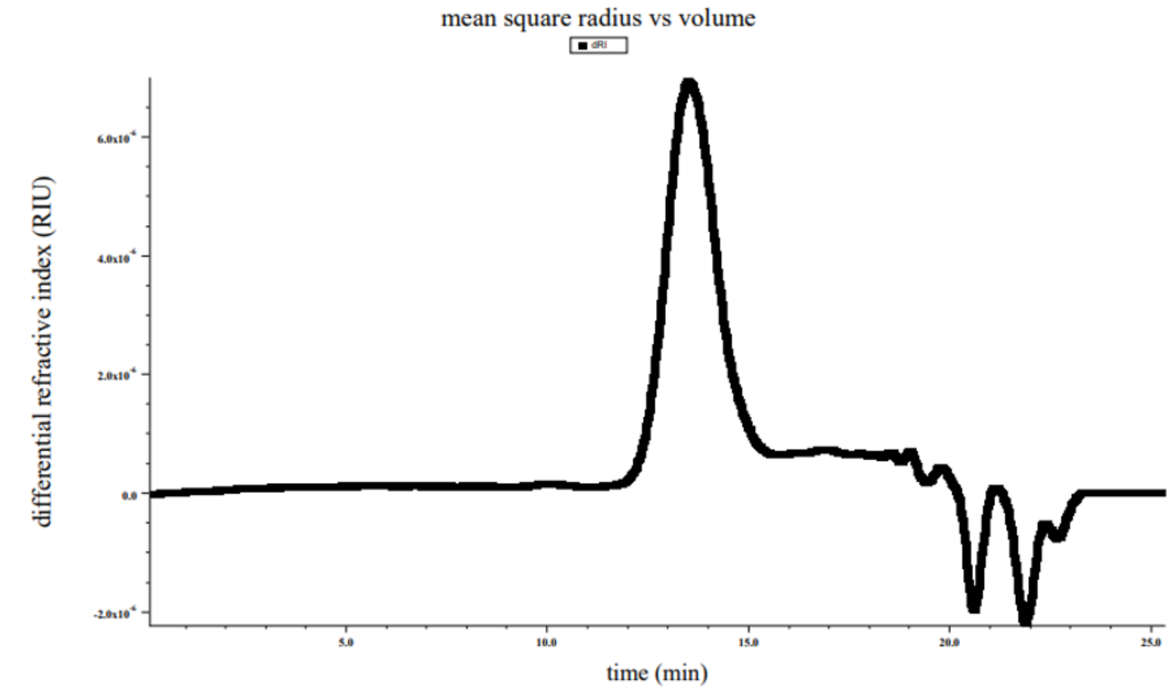

Figure S83. Refractive index of H-BCF-G1 catalyzed ROMP of COE.

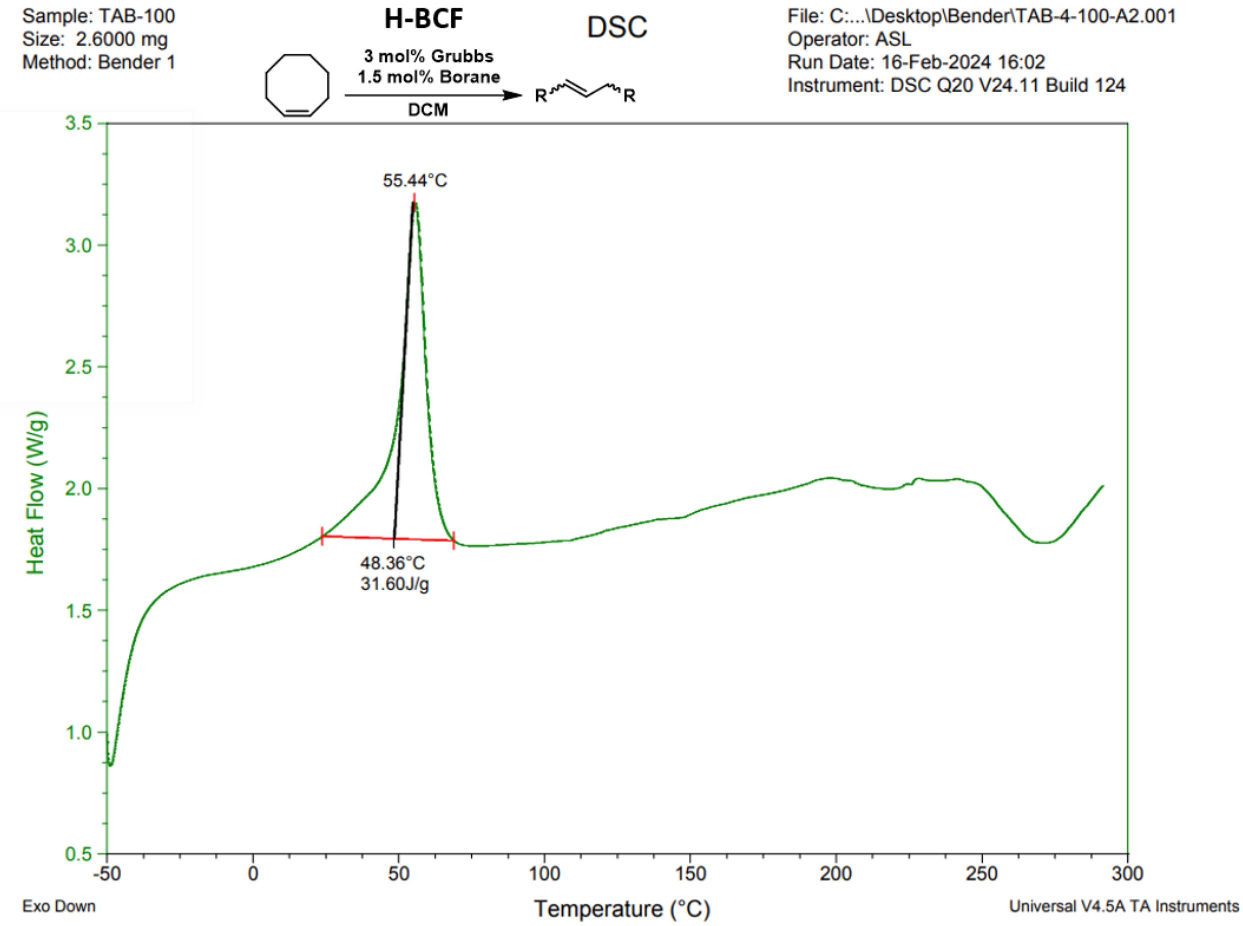

Figure S84. Glass transition point of H-BCF-G1 catalyzed ROMP of COE obtained by differential scanning calorimetry (DSC).

Cyclooctene NaBArF/G1

Configuration

Concentration Source: RI
Flow Rate: 0.700 mL/min

Light Scattering Instrument:
Cell Type: Fused Silica
Wavelength: 658.6 nm
Calibration Constant: 2.9957×10^{-5} 1/(V cm)

RI Instrument:

Solvent: THF
Temperature Correction Enabled: yes
Refractive Index: 1.402

Processing

Collection Time: Tuesday November 28, 2023 04:08:27 PM Eastern Standard Time
Processing Time: Tuesday November 28, 2023 04:33:28 PM Eastern Standard Time

Peak settings:

Peak Name	Peak 1
Peak Limits (min)	11.118 - 15.810
Light Scattering Model	Zimm
Fit Degree	1
dn/dc (mL/g)	0.1850
A2 (mol mL/g²)	0.000

Results

Peak Results

	Peak 1
**Masses**	
Injected Mass (µg)	10.00
Calculated Mass (µg)	56.27
Mass Recovery (%)	562.7
Mass Fraction (%)	100.0
**Molar mass moments (g/mol)**	
Mn	4.116×10^{4} (±26.581%)
Mp	7.625×10^{4} (±3.530%)
Mv	n/a
Mw	8.535×10^{4} (±5.539%)
Mz	1.409×10^{5} (±10.933%)

Figure S85. Astra report of NaBArF-G1 catalyzed ROMP of COE.

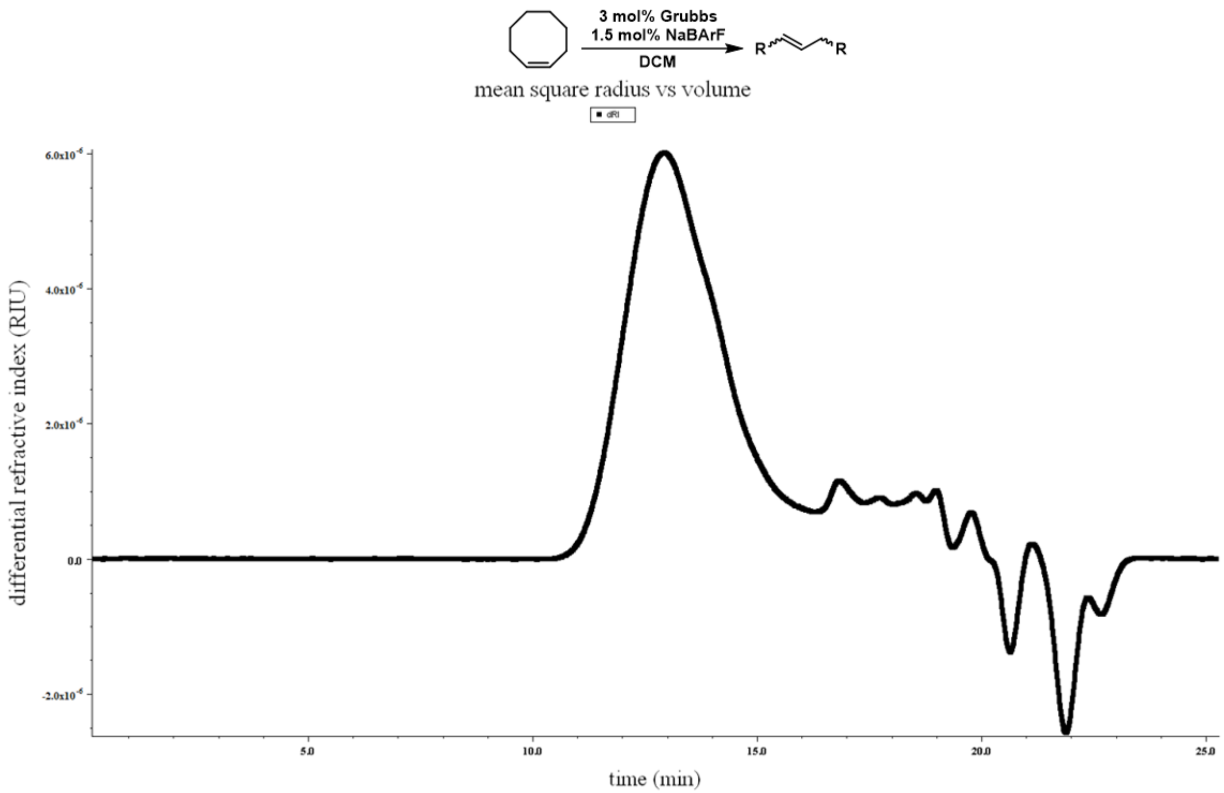

Figure S86. Refractive index of NaBArF-G1 catalyzed ROMP of COE.

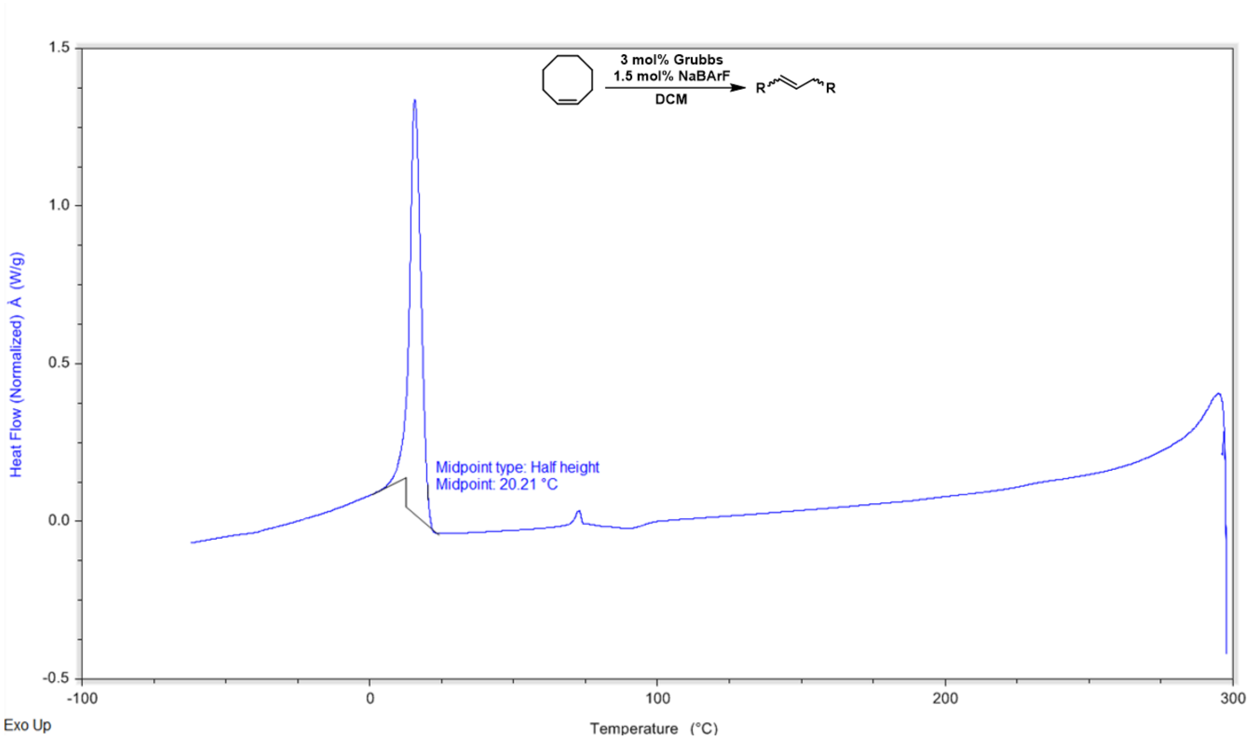

Figure S87. Glass transition point of NaBArF-G1 catalyzed ROMP of COE obtained by differential scanning calorimetry (DSC).

Calibration Curve

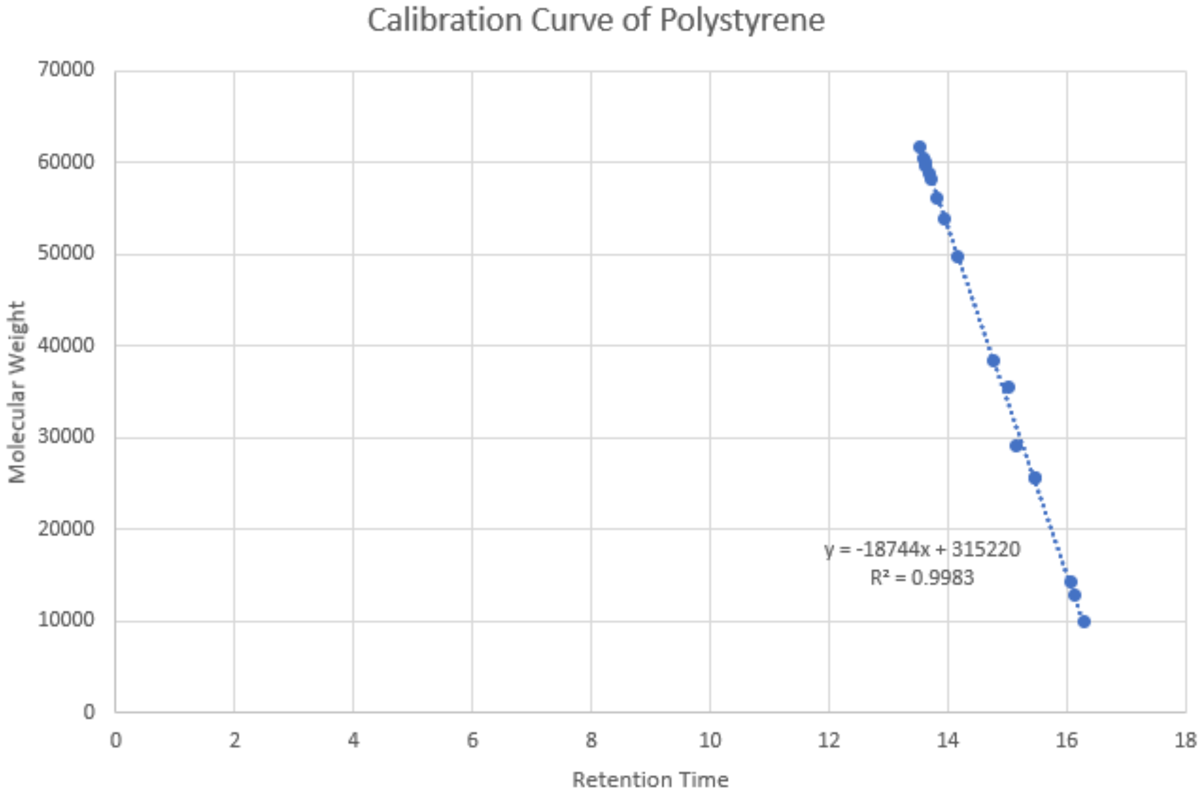

Polystyrene Size	Retention Time Low	Retention Time High	Average Retention Time	Molecular weight
200 kDa	13.129	13.729	13.429	185300
30 kDa	13.827	16.445	15.136	29230
20 kdA	13.727	16.329	15.028	35610
10 kda	14.967	17.329	16.148	12770

Figure S88. Calibration curve of polystyrene.

Polymer ¹H and ¹³C NMR spectra

1. *Cyclopentene Control ¹H NMR spectrum*

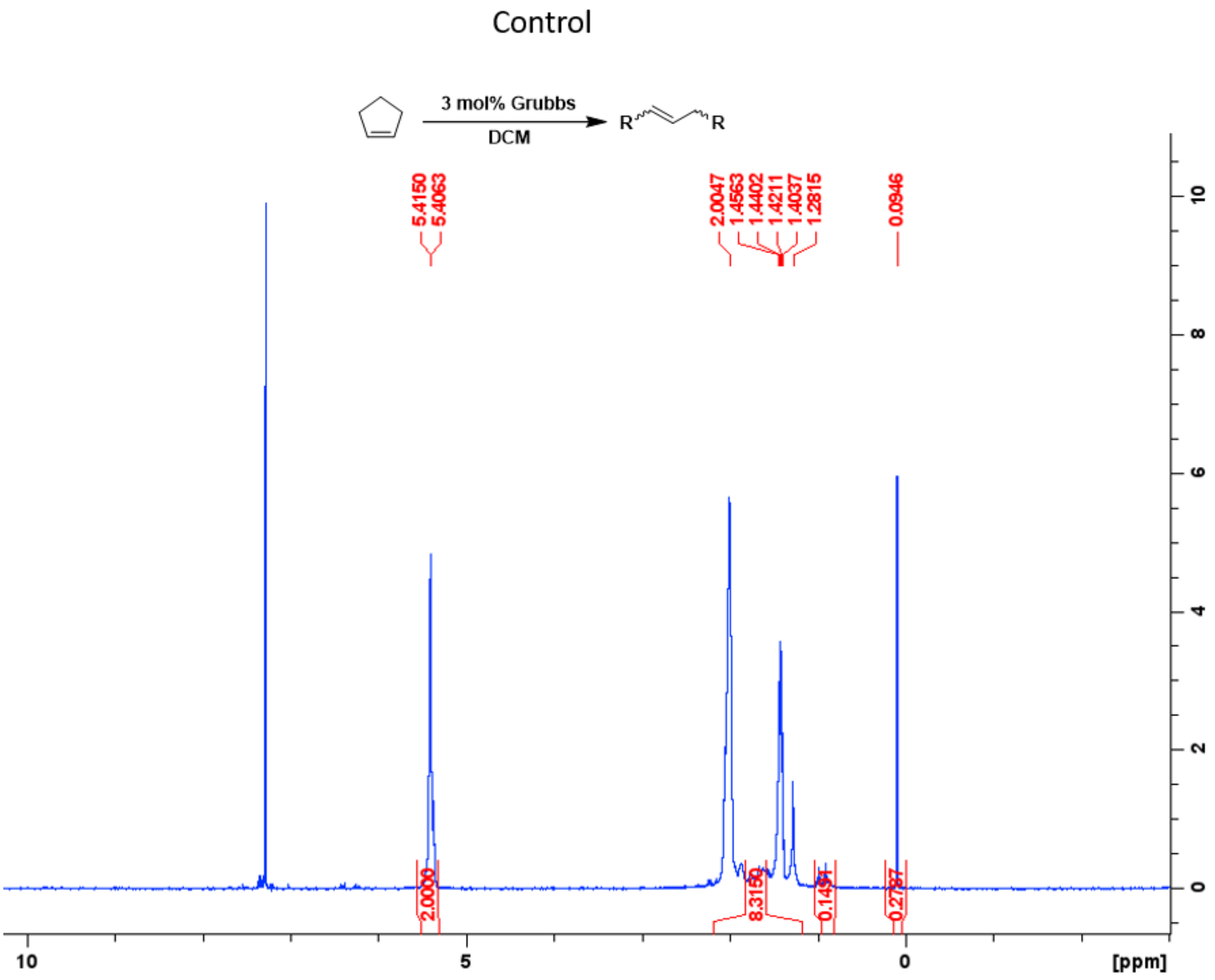

Figure S89. Cyclopentene Control ¹H NMR spectrum in $CDCl_3$.

Cyclopentene Control ^{13}C NMR spectrum

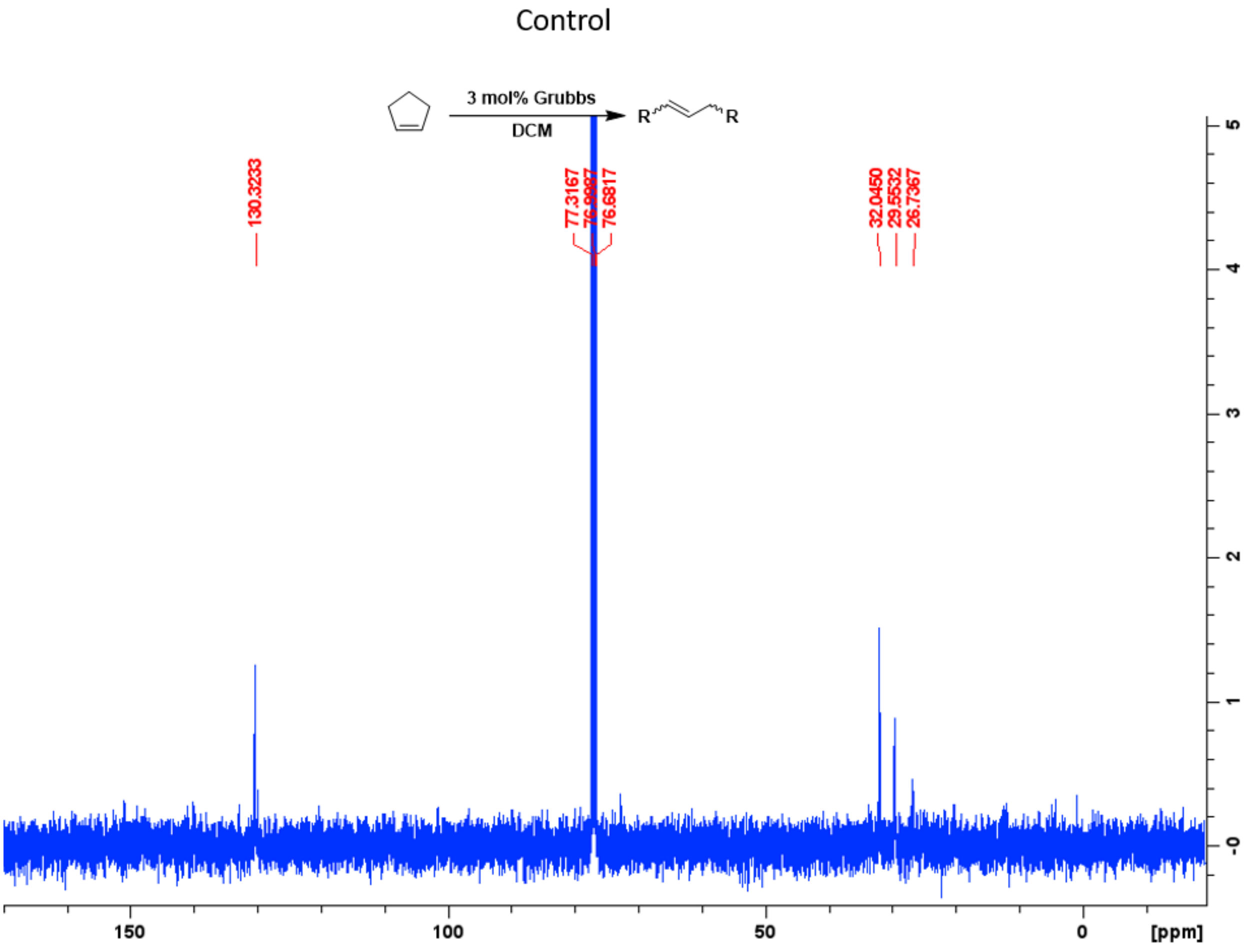

Figure S90. Cyclopentene Control ^{13}C NMR spectrum in $CDCl_3$.

1. *Cyclopentene + BCF ^{1}H NMR Spectrum*

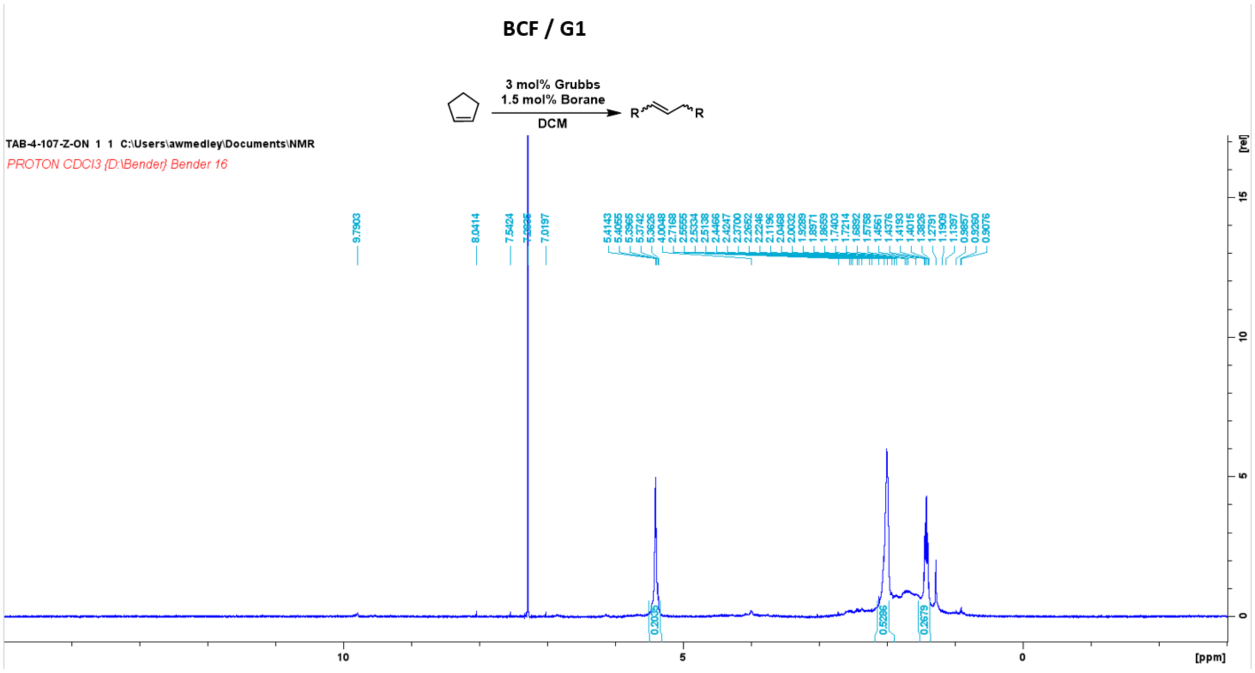

Figure S91. ^{1}H NMR spectrum of BCF-G1 catalyzed ROMP of cyclopentene in $CDCl_3$.

Cyclopentene + BCF ^{13}C NMR Spectrum

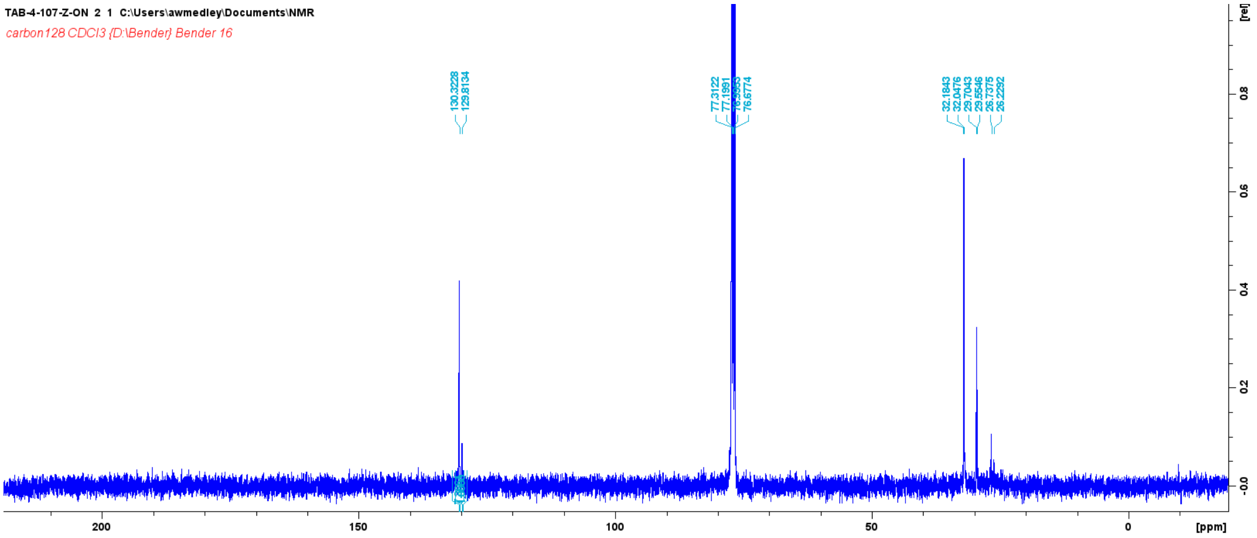

Figure S92. ^{13}C NMR spectrum of BCF-G1 catalyzed ROMP of cyclopentene in $CDCl_3$.

1. *Cyclopentene + H-BCF ^{1}H NMR Spectrum*

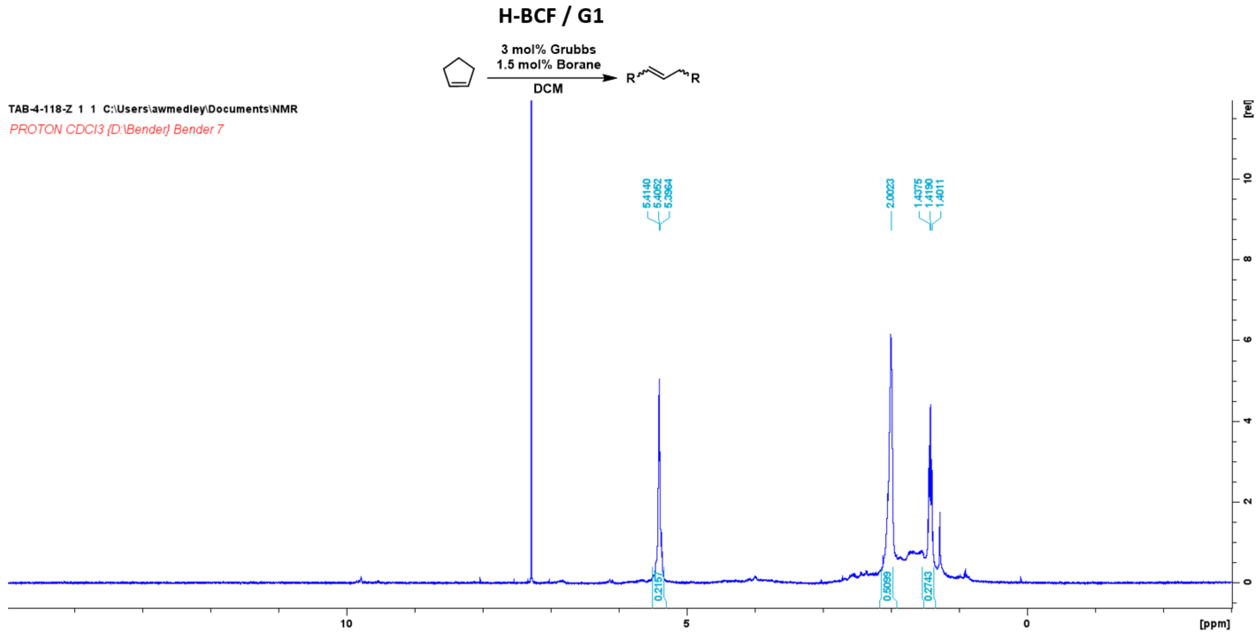

Figure S93. ^{1}H NMR spectrum of H-BCF-G1 catalyzed ROMP of cyclopentene in $CDCl_3$.

Cyclopentene + H-BCF ^{13}C NMR Spectrum

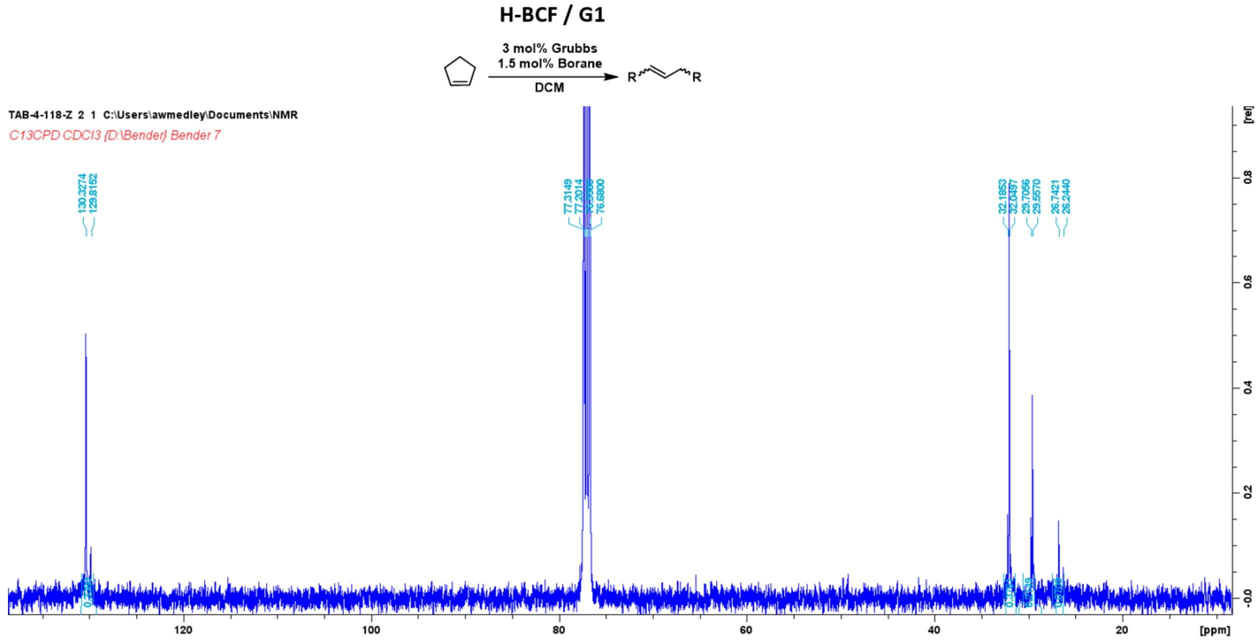

Figure S94. ^{13}C NMR spectrum of H-BCF-G1 catalyzed ROMP of cyclopentene in $CDCl_3$.

Cyclopentene + Cp-BCF ^{1}H NMR Spectrum

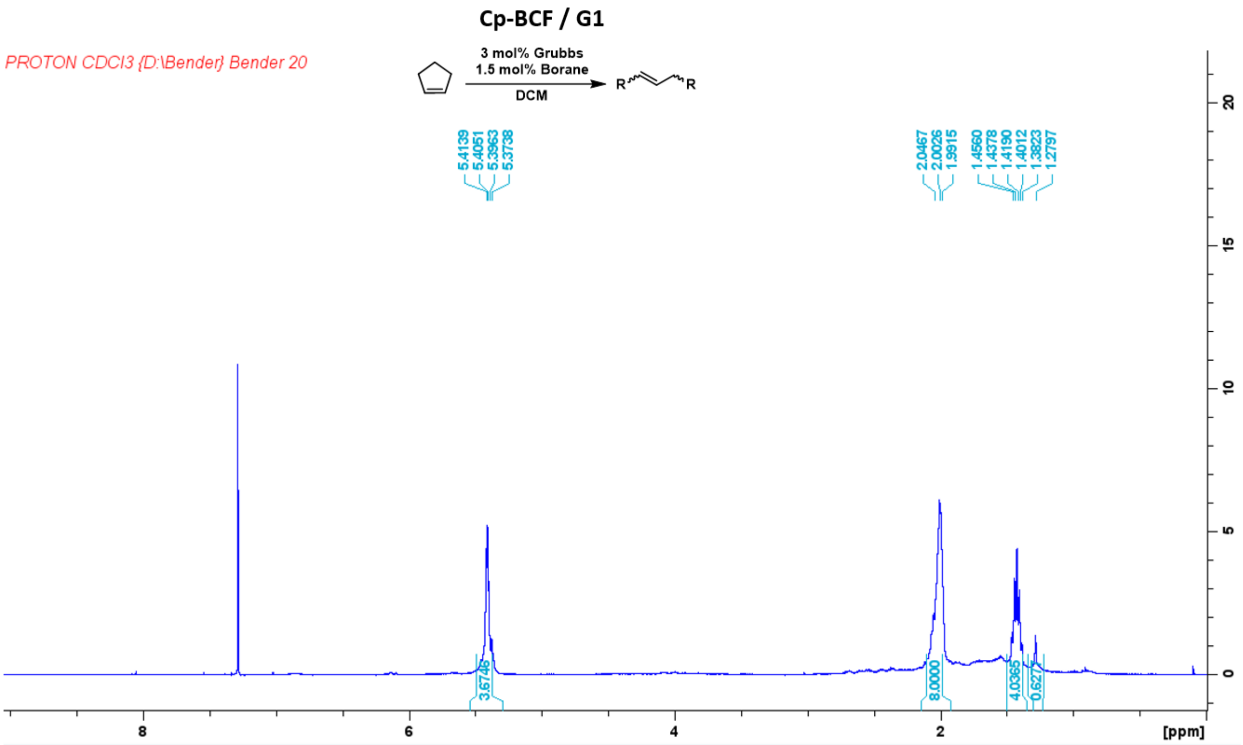

Figure S95. ^{1}H NMR spectrum of Cp-BCF-G1 catalyzed ROMP of cyclopentene in $CDCl_3$.

Cyclopentene + Cp-BCF ^{13}C NMR spectrum

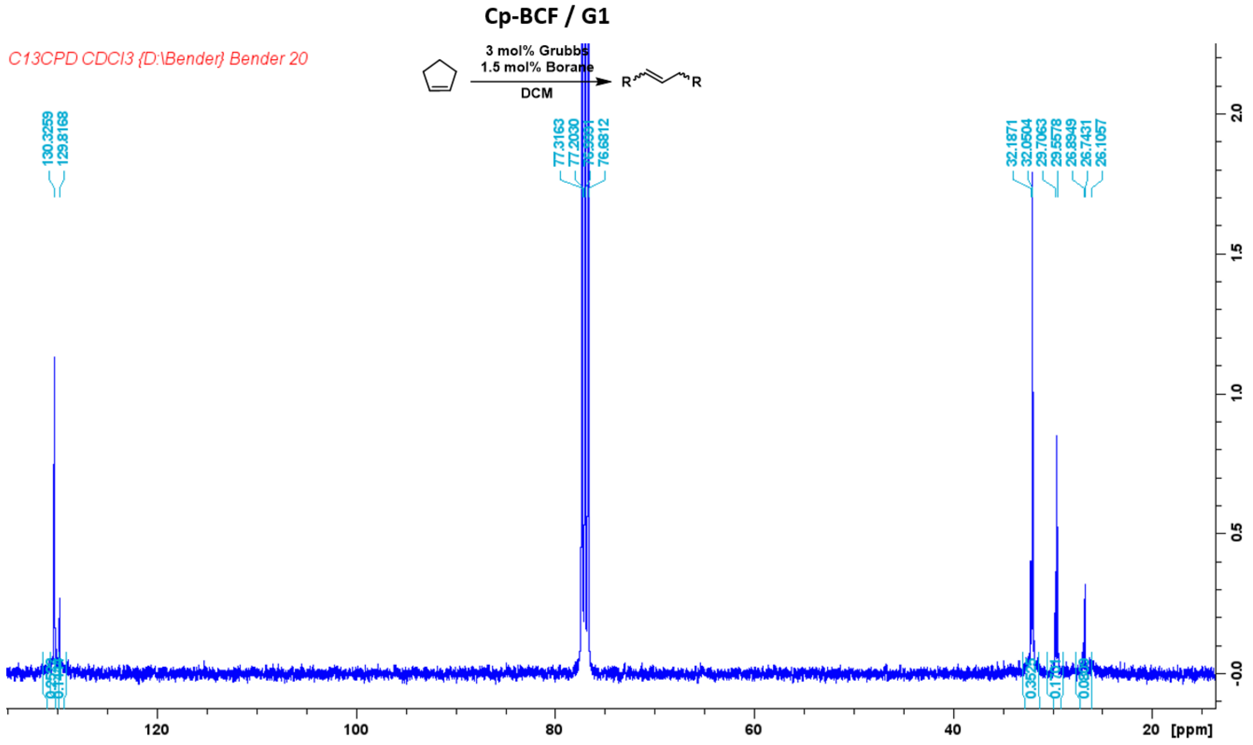

Figure S96. ^{13}C NMR spectrum of Cp-BCF-G1 catalyzed ROMP of cyclopentene in $CDCl_3$.

Cyclopentene + NaBArF ^1H NMR spectrum

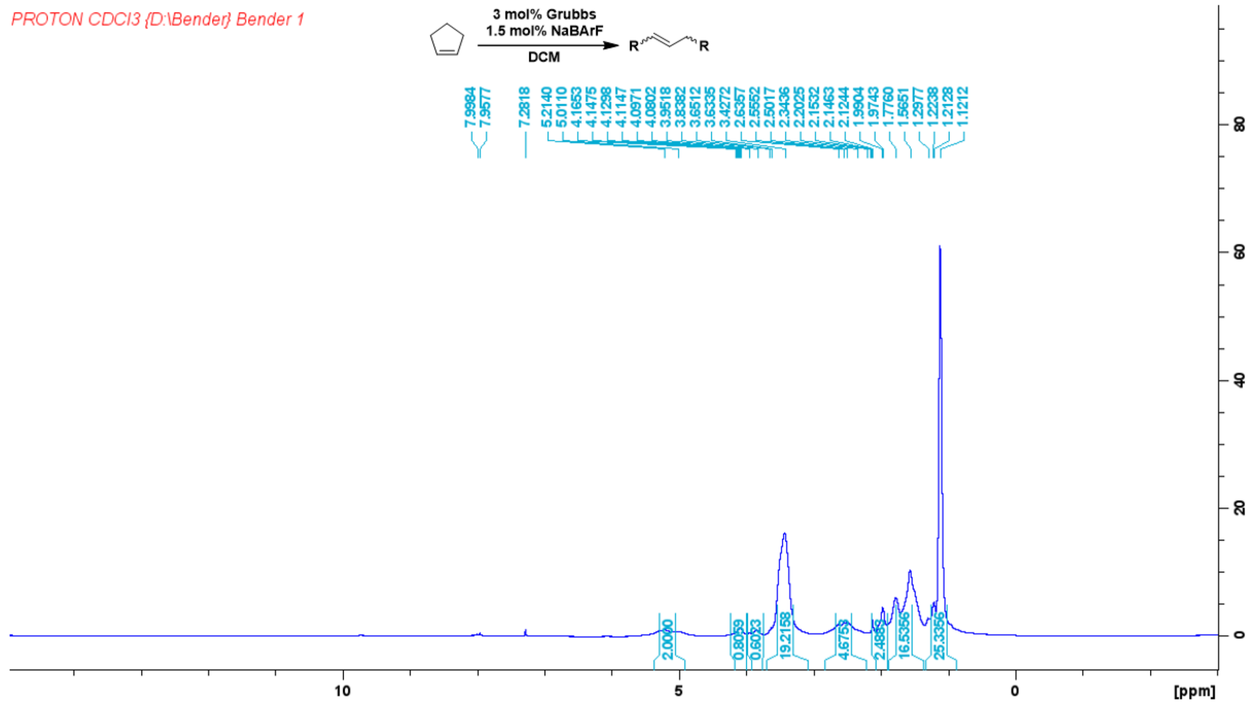

Figure S97. ^1H NMR spectrum of NaBArF-G1 catalyzed ROMP of cyclopentene in $CDCl_3$.

Cyclopentene + NaBArF ¹³C NMR spectrum

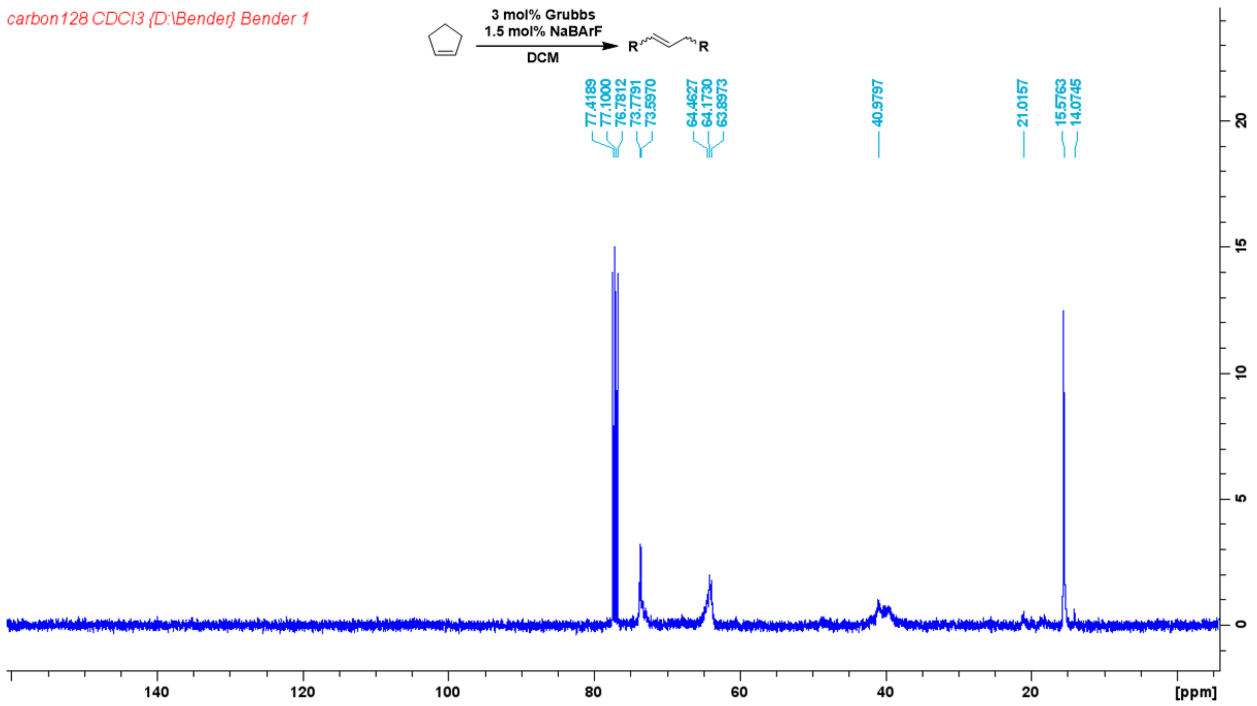

Figure S98. ¹³C NMR spectrum of NaBArF-G1 catalyzed ROMP of cyclopentene in $CDCl_3$.

Cyclooctene Control ¹H NMR spectrum

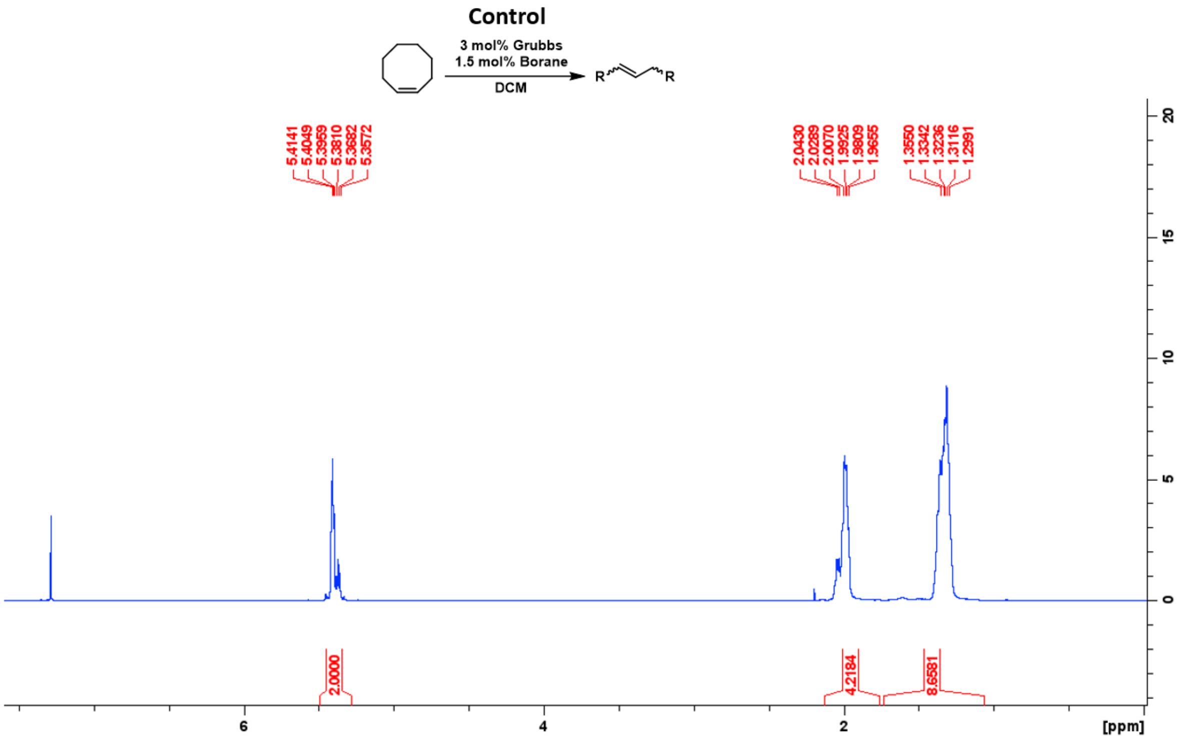

Figure S99. ¹H NMR spectrum of G1 catalyzed ROMP of COE in $CDCl_3$.

Cyclooctene Control ¹³C NMR spectrum

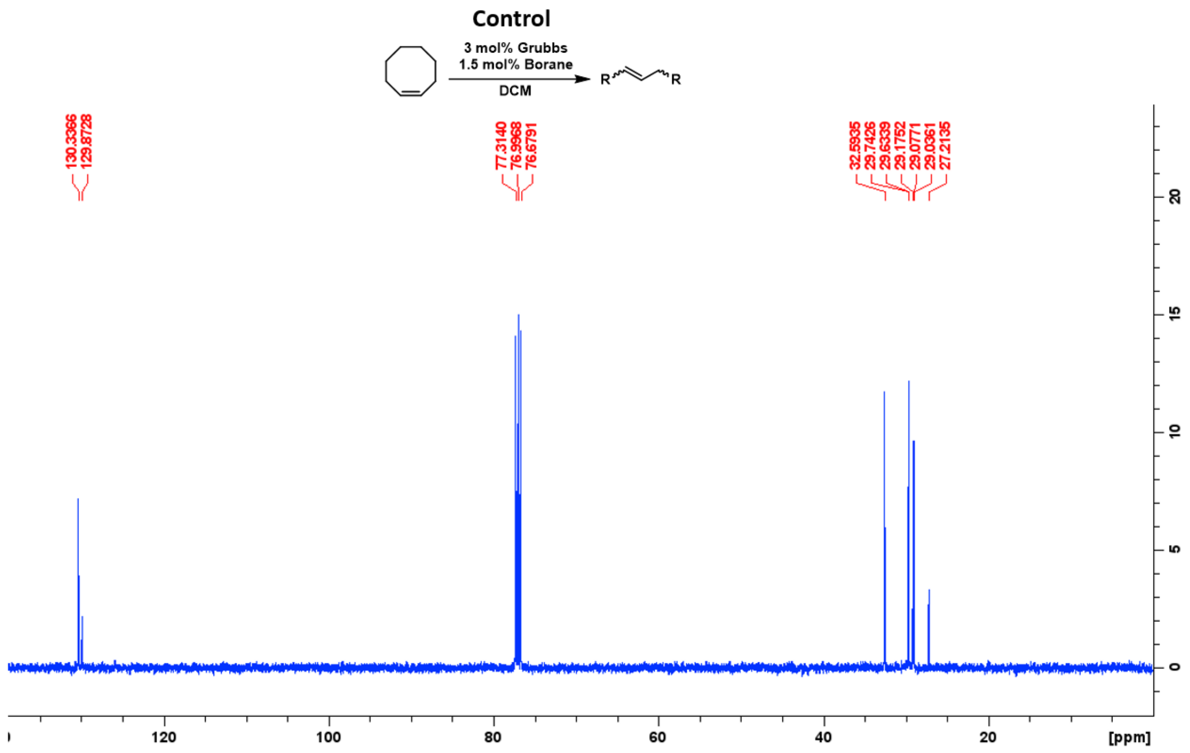

Figure S100. ^{13}C NMR spectrum of G1 catalyzed ROMP of COE in $CDCl_3$.

Cyclooctene + BCF Spectra

Cyclooctene + BCF ^1H NMR Spectrum

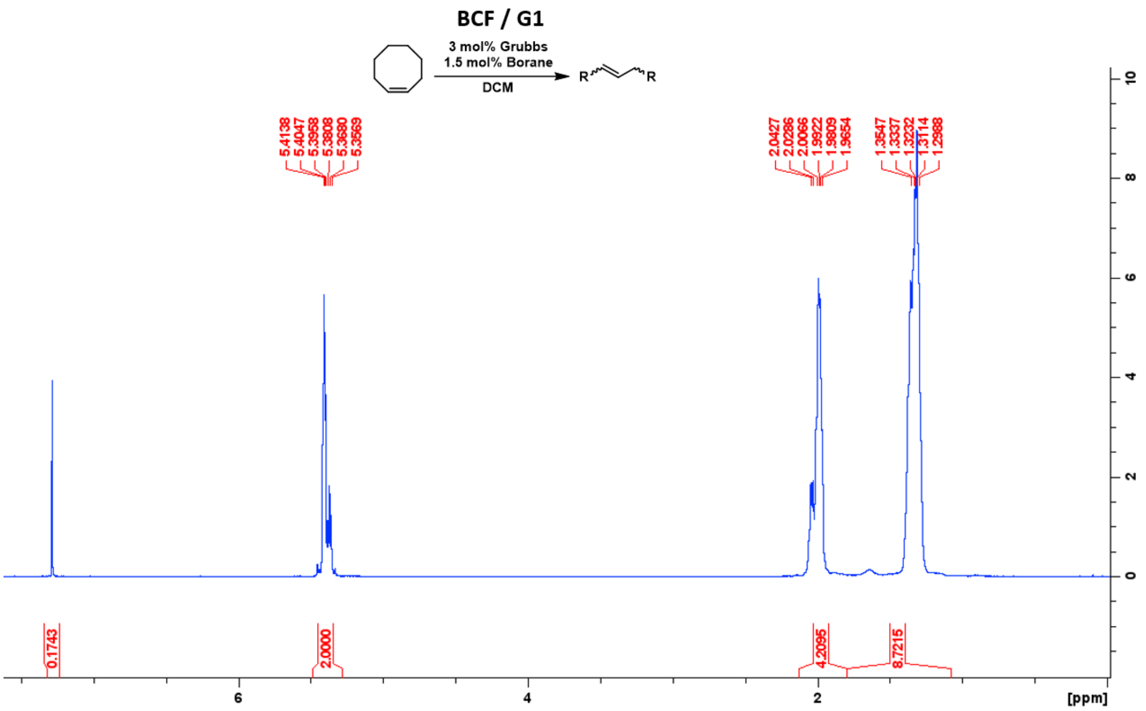

Figure S101. ¹H NMR spectrum of BCF-G1 catalyzed ROMP of COE in $CDCl_3$.

Cyclooctene + BCF ¹³C NMR spectrum

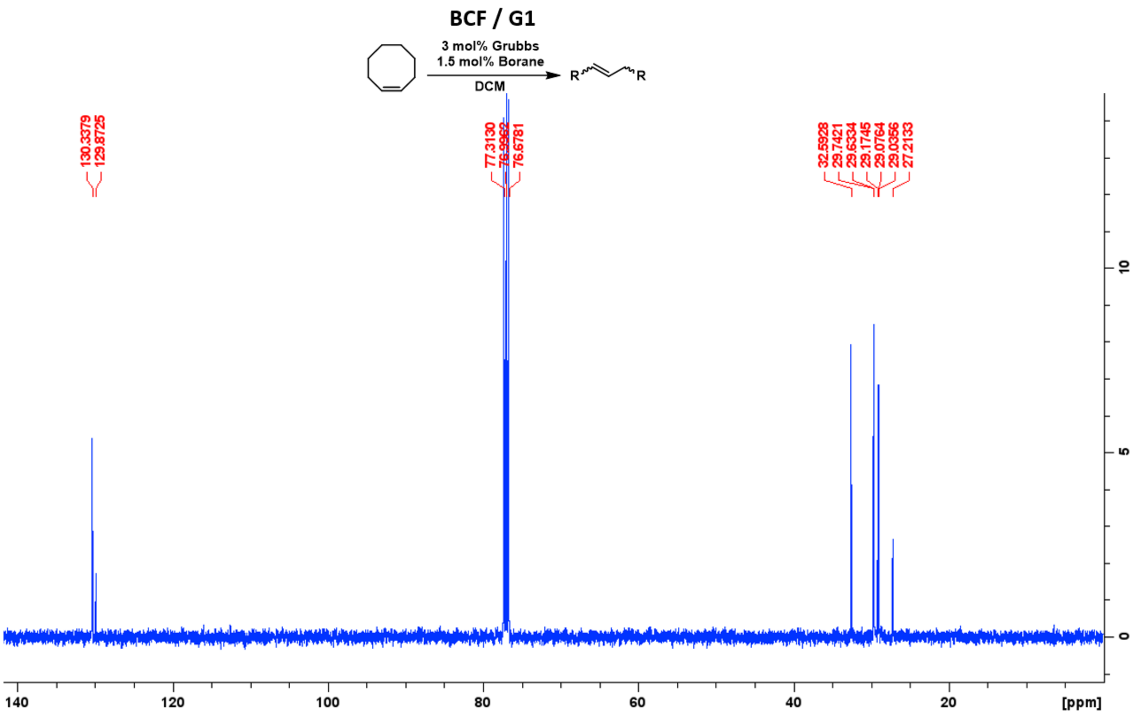

Figure S102. ¹³C NMR spectrum of BCF-G1 catalyzed ROMP of COE in $CDCl_3$.

Cyclooctene + H-BCF Spectra

Cyclooctene + H-BCF ¹H NMR spectrum

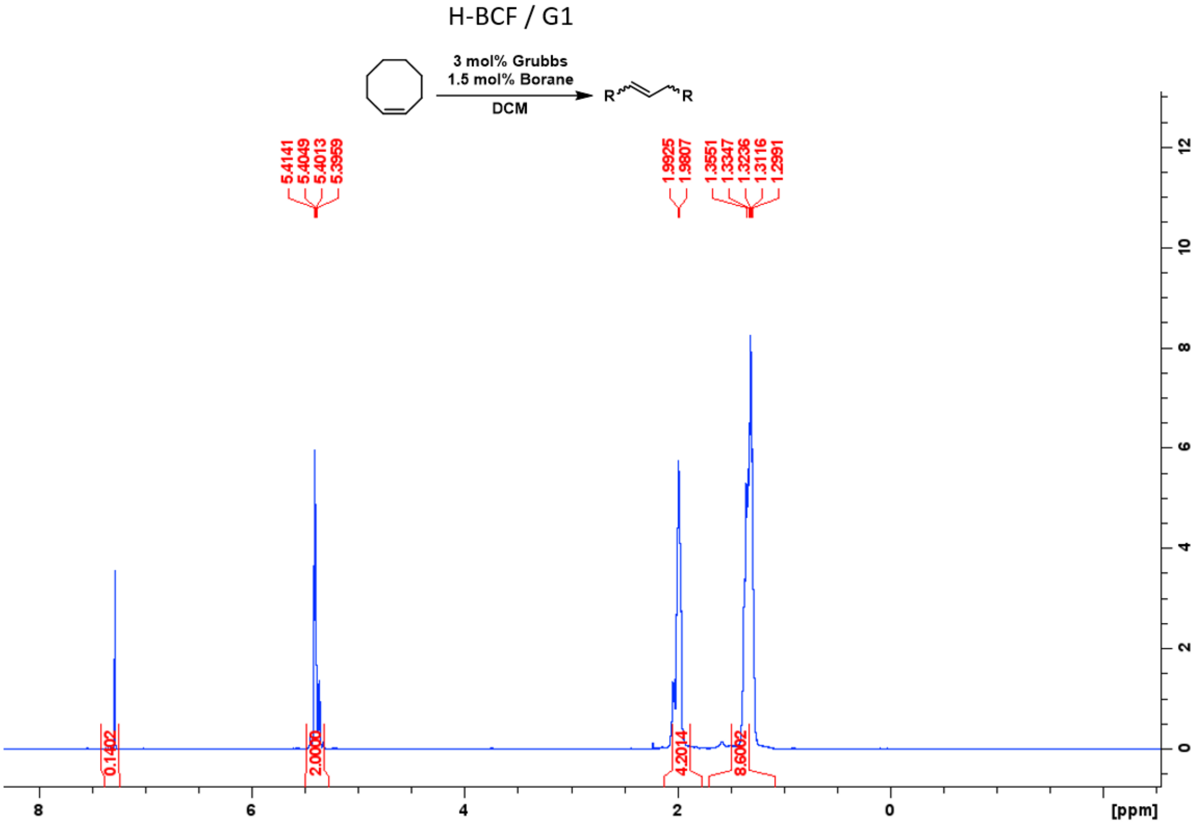

Figure S103. ¹H NMR spectrum of H-BCF-G1 catalyzed ROMP of COE in $CDCl_3$.

Cyclooctene + H-BCF ¹³C NMR spectrum

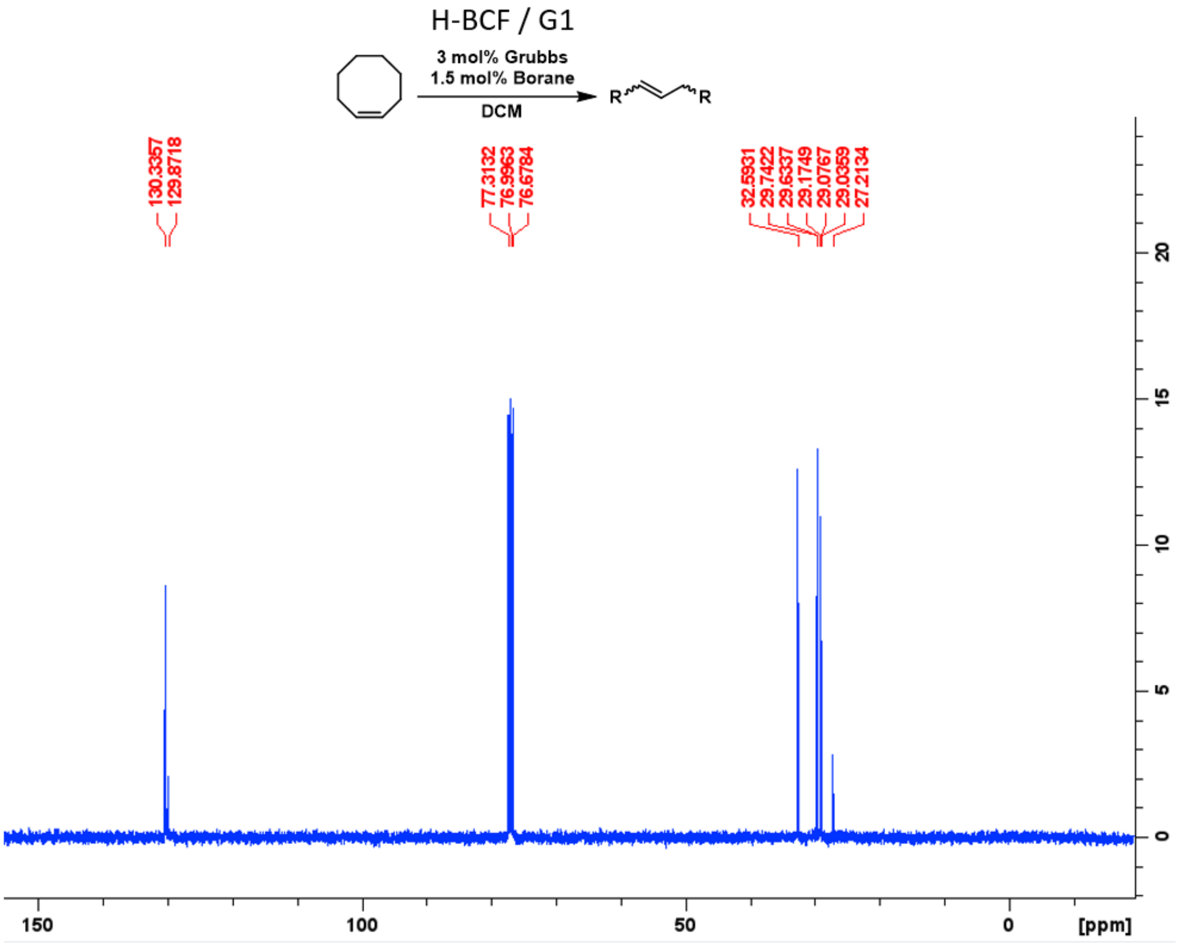

Figure S104. ^{13}C NMR spectrum of H-BCF-G1 catalyzed ROMP of COE in $CDCl_3$.

Cyclooctene + Cp-BCF spectra

Cyclooctene + Cp-BCF ¹H NMR spectrum

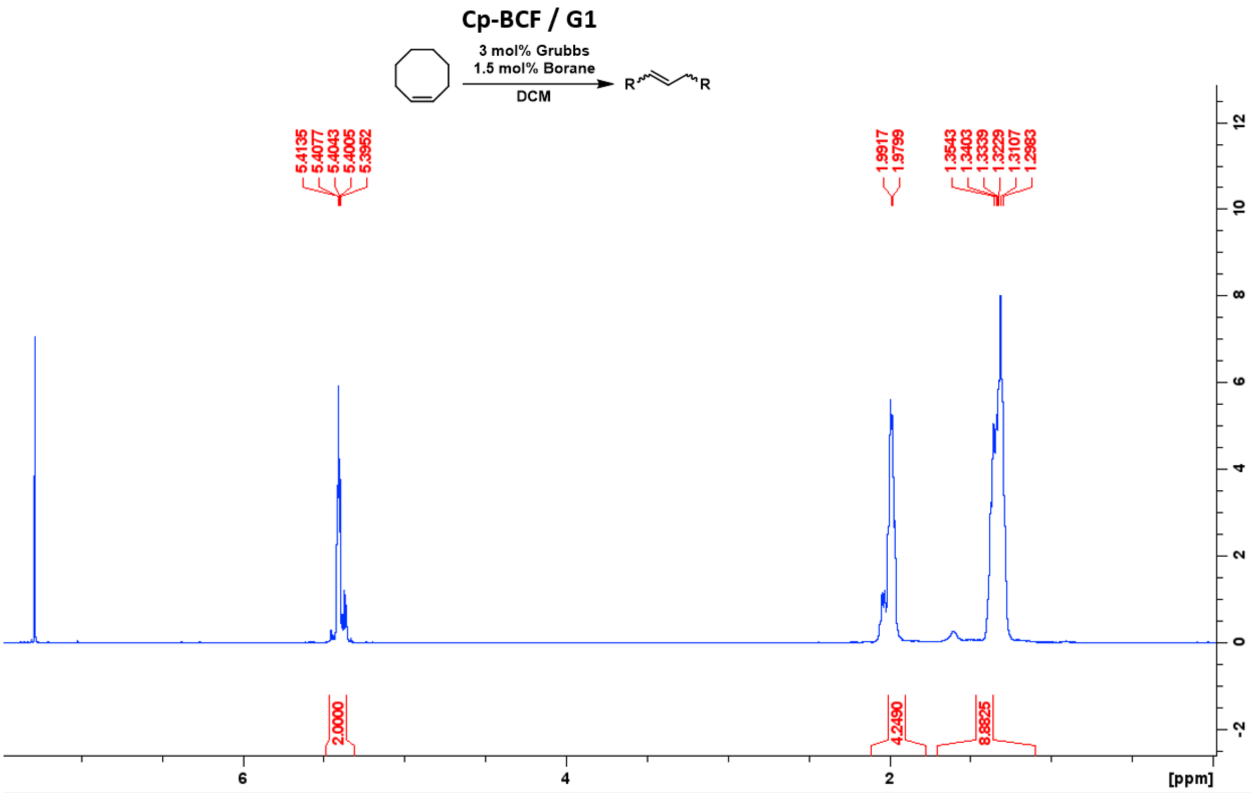

Figure S105. ¹H NMR spectrum of Cp-BCF-G1 catalyzed ROMP of COE in $CDCl_3$.

Cyclooctene + Cp-BCF ^{13}C NMR spectrum

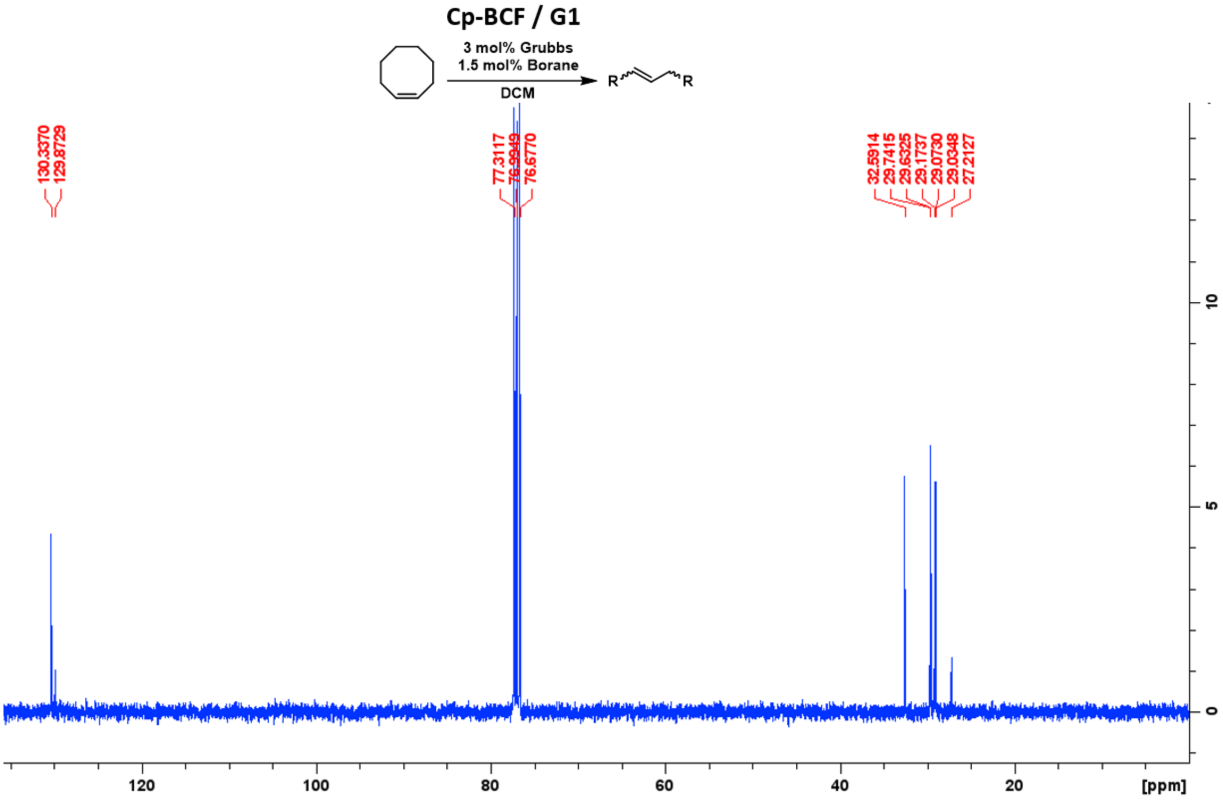

Figure S106. ^{13}C NMR spectrum of Cp-BCF-G1 catalyzed ROMP of COE in $CDCl_3$.

Cyclooctene + NaBArF spectra

Cyclooctene + NaBArF ¹H NMR spectrum

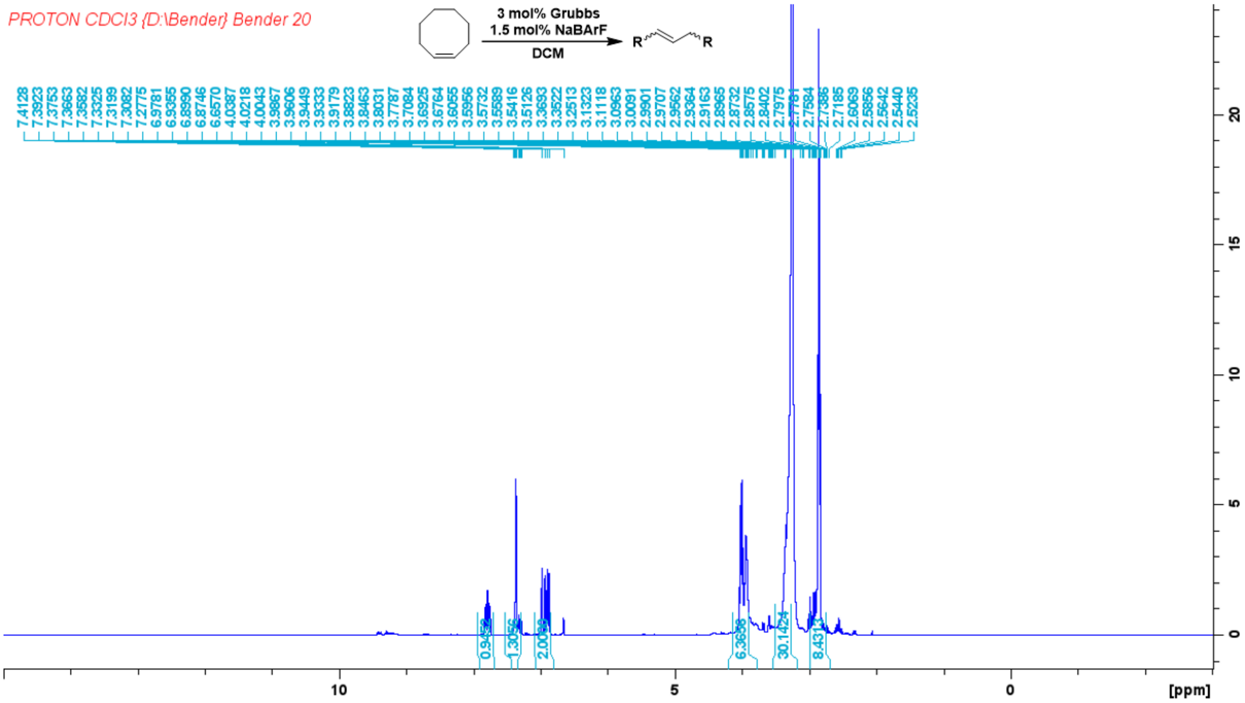

Figure S107. ¹H NMR spectrum of NaBArF-G1 catalyzed ROMP of COE in $CDCl_3$.

Cyclooctene + NaBArF ¹³C NMR spectrum

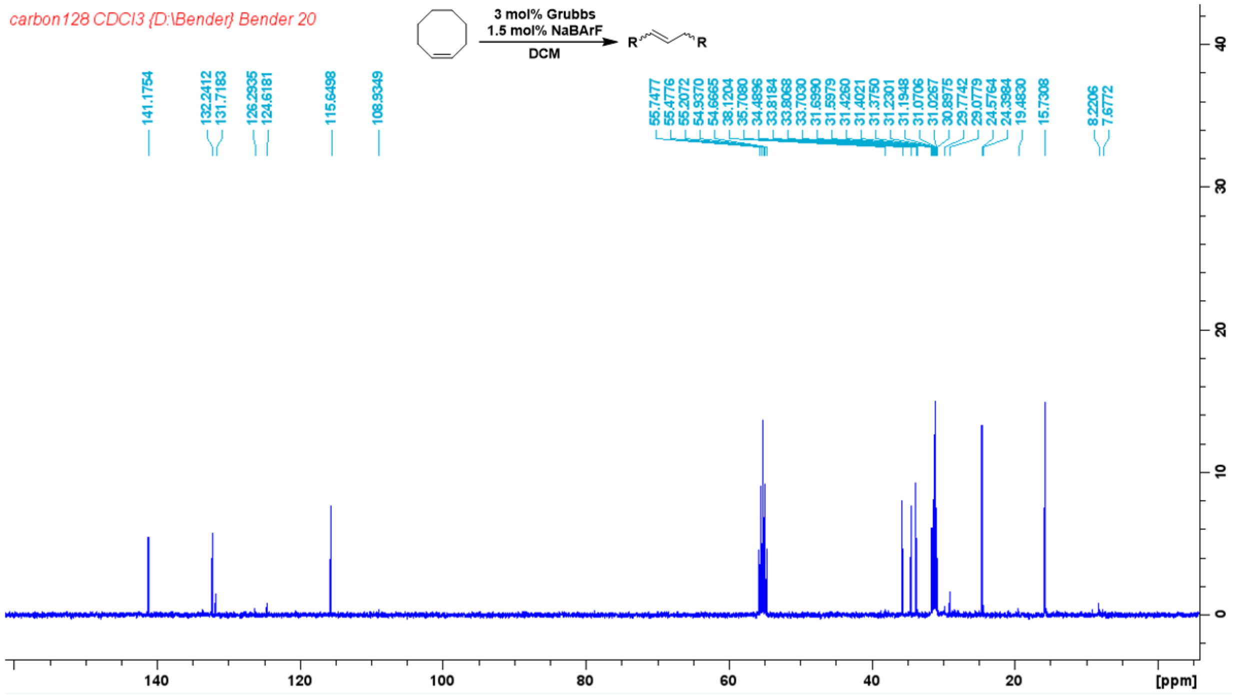

Figure S108. ¹³C NMR spectrum of NaBArF-G1 catalyzed ROMP of COE in $CDCl_3$.

References:

1. Fulmer, G. R.; Miller, A. J. M.; Sherden, N. H.; Gottlieb, H. E.; Nudelman, A.; Stoltz, B. M.; Bercaw, J. E.; Goldberg, K. I. *Organometallics* **2010**, 29, 2176–2179.

2. Parks, D. J.; Piers, W. E.; Yap, G. P. *Organometallics* **1998**, 17, 5492–5503.

3. Normand, A. T.; Richard, P.; Balan C.; Daniluc C. G.; Kehr, G.; Erker, G.; Gendre, P. L. *Organometallics* **2015**, 34, 2000–2011.

4. Nieczypor, P.; W. N. M. van Leuuwen, P.; Mol, J. C.; Lutz, M.; Spek, A. *J. Organomet. Chem.* **2001**, 625, 58-66.
